# The Protozoan *Trichomonas vaginalis* Targets Bacteria with Laterally Acquired NlpC/P60 Peptidoglycan Hydrolases

**DOI:** 10.1128/mBio.01784-18

**Published:** 2018-12-11

**Authors:** Jully Pinheiro, Jacob Biboy, Waldemar Vollmer, Robert P. Hirt, Jeremy R. Keown, Anastasiia Artuyants, Moyra M. Black, David C. Goldstone, Augusto Simoes-Barbosa

**Affiliations:** a School of Biological Sciences, University of Auckland, Auckland, New Zealand; b Centre for Bacterial Cell Biology, Institute for Cell and Molecular Biosciences, Newcastle University, Newcastle upon Tyne, United Kingdom; c Institute for Cell and Molecular Biosciences, Newcastle University, Newcastle upon Tyne, United Kingdom; d Maurice Wilkins Centre for Molecular Biodiscovery, Auckland, New Zealand; University of California, Los Angeles

**Keywords:** lateral gene transfer, NlpC/P60, peptidoglycan, *Trichomonas vaginalis*, peptidoglycan hydrolases

## Abstract

The human eukaryotic pathogen Trichomonas vaginalis causes trichomoniasis, a prevalent sexually transmitted infection. This extracellular protozoan is intimately associated with the human vaginal mucosa and microbiota, but key aspects of the complex interactions between the parasite and the vaginal bacteria remain elusive. We report that T. vaginalis has acquired, by lateral gene transfer from bacteria, genes encoding peptidoglycan hydrolases of the NlpC/P60 family. Two of the T. vaginalis enzymes were active against bacterial peptidoglycan, retaining the active-site fold and specificity as dl-endopeptidases. The endogenous NlpC/P60 genes are transcriptionally upregulated in T. vaginalis in the presence of bacteria. The overexpression of an exogenous copy enables the parasite to outcompete bacteria from mixed cultures, consistent with the biochemical activity of the enzyme. Our study results highlight the relevance of the interactions of this eukaryotic pathogen with bacteria, a poorly understood aspect of the biology of this important human parasite.

## Abstract (precis)

Trichomonas vaginalis is a parasitic protozoan of the human urogenital tract that causes trichomoniasis, a very common sexually transmitted disease. Despite residing extracellularly and in close association with the vaginal bacteria (i.e., the microbiota), very little is known about the nature of the parasite-bacterium interactions. Our study showed that this parasite had acquired genes from bacteria which retained their original function. They produce active enzymes capable of degrading peptidoglycan, a unique polymer of the bacterial cell envelope, helping the parasite to outcompete bacteria in mixed cultures. This study was the first to show that a laterally acquired group of genes enables a eukaryotic mucosal pathogen to control bacterial population. We highlight the importance of understanding the interactions between pathogens and microbiota, as the outcomes of these interactions are increasingly understood to have important implications on health and disease.

## INTRODUCTION

Trichomonas vaginalis is a flagellated protozoan parasite that causes human trichomoniasis, the most common nonviral sexually transmitted infection worldwide (1). It leads to vaginitis and gyneco-obstetric complications (2–7). Also, trichomoniasis facilitates the transmission of the human immunodeficiency virus (8). This extracellular parasite displays an intimate association with the vaginal microbiota, but the molecular and cellular basis of this microbial interaction is poorly understood. Since trichomoniasis is apparently associated with microbial dysbiosis (9), *Trichomonas*-microbiota interactions might significantly affect the disease profile (10–12).

T. vaginalis might have evolved from an enteric to a genitourinary mucosal environment (13), experiencing a recent genome expansion through a combination of gene duplications, transposable element transfers, and lateral gene transfers (LGTs). Its unusually large genome (∼170 Mbp) displays an extraordinary coding capacity of ∼60,000 predicted protein-coding genes (14, 15), among which ∼30,000 are evidently expressed (16, 17). The coevolution of *Trichomonas* and microbiota may have significantly shaped the genome of this parasite by providing new functionalities and selective advantages for colonization of this habitat (15, 18, 19).

A considerable number of genes have been acquired by eukaryotes from prokaryotes by LGT (15, 20–22), including an increasing number of genes encoding enzymes that degrade or remodel peptidoglycan (PG), an essential component of the bacterial cell envelope (19, 22–26). Two recent reports demonstrated that these LGT-acquired genes provided a new function that is beneficial to the recipient eukaryotic organisms: the control of the presence or abundance of bacteria (22, 25, 26). In the ecological context of the *Trichomonas* microbiota, acquisition of PG-degrading enzymes may explain why infections are preferentially accompanied by certain species of vaginal bacteria (9). Indeed, NlpC/P60-like genes are strong LGT candidates in T. vaginalis genome (14, 18). NlpC/P60 proteins were originally described as bacterial cell wall endopeptidases cleaving the d-γ-glutamyl-*meso*-diaminopimelate linkage in PG (27, 28). They display the conserved catalytic triad of papain-like thiol peptidases and may carry additional domains specifying substrate binding, a signal peptide (SP), or transmembrane regions for proper subcellular localization (28).

This study aimed to (i) understand the structural diversity and evolutionary origins of T. vaginalis NlpC/P60-like genes, (ii) characterize the structure-function relationship for a selection of these enzymes by determining their crystal structures and activities against PG, and (iii) examine the potential role of NlpC/P60 enzymes in parasite-bacterium interactions. To the best of our knowledge, this was the first study to report the preservation of fully functional LGT-derived PG-degrading enzymes in a eukaryotic pathogen of the mucosa.

## RESULTS

### Primary structural diversity and phylogeny of Trichomonas vaginalis NlpC genes.

**FIG 1:**
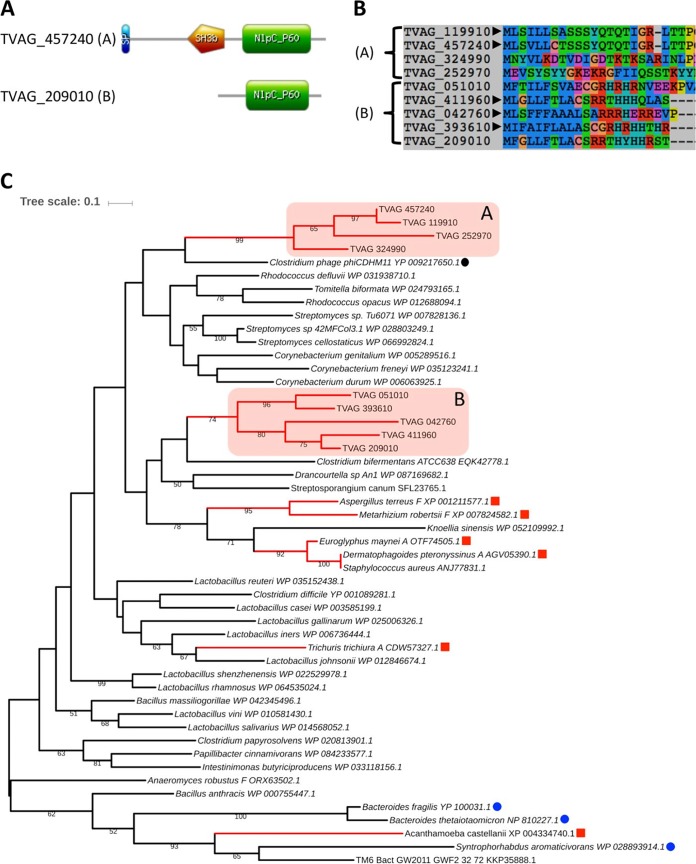
Bioinformatic analyses of the T. vaginalis NlpC/P60 proteins. (A) Structural organization for one representative each (locus tags are indicated) of the two clusters (clusters A and B) of T. vaginalis NlpC/P60 proteins (see Table S1 and main text). Two TvNlpC/P60 proteins (cluster A), named NlpC_A1 (TVAG_119910) and NlpC_A2 (TVAG_457240 [illustrated]), possess a bacterial SH3 domain (orange pentagon) identified in addition to the NlpC/P60 domain (green rectangle). (B) N-terminal protein alignment for all nine T. vaginalis NlpC/P60 proteins. Locus tags are indicated, and the arrowheads indicate the five entries with inferred signal peptide (Table S1). Members of cluster A are more similar to each other then they are to members of cluster B and vice versa. (C) Phylogenetic relationship inferred by maximum likelihood from a protein alignment of 104 residues with the model LG + G4 + I. Bootstrap support values above 50% are indicated, and the scale bar shows the inferred number of substitutions per site. The two TvNlpC/P60 protein clusters were recovered as distinct clans (red boxes A and B). Eukaryotic sequences from non-*Trichomonas* species are indicated by red squares and branches. Blue and black circles are from three Gram-negative bacteria and from a phage, respectively. The RefSeq accession numbers of the sequences are indicated. Among eukaryotes, the letters “A” and “F” following the species names refer to animal (metazoan) and fungal, respectively.

The genome of T. vaginalis (14) encodes nine NlpC/P60 proteins with a PF00866 domain (T. vaginalis NlpC/P60 [TvNlpC/P60]) split into two orthologous groups (29), NlpC_A1-4 and NlpC_B1-5. A bacterial-type SH3 domain (PF08239; SH3b) and signal peptide (SP) were inferred in NlpC_A1/A2 and NlpC_B1/B3/B5 (Fig. 1A; see also Table S1 and Fig. S1 in the supplemental material). However, the N termini within each group were similar, suggesting that all nine proteins may have functional SPs despite not all being recognized by the current tools (Fig. 1B; see also Fig. S1). The employment of the members of the two orthologous groups as search queries produced distinct taxonomic reports for the top 100 BLASTP hits against the nonredundant protein NCBI database (Tables S2 and S3), with these hits being mainly derived from bacterial members of the *Firmicutes* and *Actinobacteria*. The high alien index (AI) values calculated from BLAST hit lists (Table S1) (30) for all nine proteins (AI value range, 13.8 to 29.6) were consistent with the hypothesis that TvNlpC/P60 genes were acquired from bacteria by LGT (14, 18). A few proteins from *Clostridium*-infecting phages were also observed in the BLASTP list (Table S2 and S3). Sensitive Delta-BLAST searches restricted to nonredundant eukaryotic proteins led to results showing a small cohort of hits from protists, fungi, and animals (Table S4). No hits were observed for the trichomonad Tritrichomonas foetus (31), suggesting that the LGT(s) in T. vaginalis was experienced by an ancestor of T. vaginalis following the speciation event that separated the two lineages (13).

10.1128/mBio.01784-18.1 FIG S1 Sequence comparison of T. vaginalis NlpC/P60 proteins. (A) Multiple-sequence alignment of T. vaginalis NplC/P60 proteins with selected homologues. The alignment is focused on the conserved NlpC/P60 domain. The positions of residues for the protein encoded by TVAG_119910 are indicated at the beginning of each block of the alignment. The residues of the catalytic Cys-His-His triad are boxed and show complete conservation among aligned sequences. The position of indels (dashes) for the four T. vaginalis sequences from clan A are distinct from those of the five sequences from clan B and are also differentially shared with the sequence from the phage infecting Clostridium difficile (accession no. YP_009217650.1) and with the sequences from three bacterial species (*Drancourtella* sp. [accession no. WP_087169682.1], Streptosporangium canum [accession no. SFL23765.1], and Clostridium bifermentans [accession no. EQK42778.1]) that also cluster in the phylogenetic tree with T. vaginalis sequences from clan A and clan B, respectively (see Fig. 1). This further supports the notion of two distinct LGT events occurring with the *Trichomonas* lineage for these NlpC/P60-encoding genes, which were followed by gene duplications events that led to the occurrence of a total of nine TvNlpC/P60 genes. (B) Comparison of the structural organizations of the nine T. vaginalis NlpC/P60 proteins and the other six eukaryotic NlpC/P60 proteins included in the phylogenetic analyses (Fig. 1C; see also Table S1). Download FIG S1, PDF file, 0.1 MB. Copyright © 2018 Pinheiro et al. 2018 Pinheiro et al. This content is distributed under the terms of the Creative Commons Attribution 4.0 International license.

10.1128/mBio.01784-18.4 TABLE S1 (A) An overview of T. vaginalis NlpC/P60 genes, expression data, and protein features and (B) the corresponding alien index calculations. TvNlpC/P60 genes/proteins were sorted by clan A (in blue) and clan B (in green) per the phylogeny data. Download Table S1, PDF file, 0.1 MB. Copyright © 2018 Pinheiro et al. 2018 Pinheiro et al. This content is distributed under the terms of the Creative Commons Attribution 4.0 International license.

10.1128/mBio.01784-18.5 TABLE S2 Taxonomic BlastP report for query NlpC_A1/TVAG_119910/XP_001276902 against the NCBI nr protein database. Download Table S2, PDF file, 1.0 MB. Copyright © 2018 Pinheiro et al. 2018 Pinheiro et al. This content is distributed under the terms of the Creative Commons Attribution 4.0 International license.

10.1128/mBio.01784-18.6 TABLE S3 Taxonomic BlastP report for query NlpC_B1/TVAG_393610/XP_001326856 against the NCBI nr protein database. Download Table S3, PDF file, 1.1 MB. Copyright © 2018 Pinheiro et al. 2018 Pinheiro et al. This content is distributed under the terms of the Creative Commons Attribution 4.0 International license.

10.1128/mBio.01784-18.7 TABLE S4 Taxonomic Delta-Blast report for query NlpC_A1/TVAG_119910/XP_001276902 against the NCBI nr protein database restricted to eukaryotes. Download Table S4, PDF file, 0.2 MB. Copyright © 2018 Pinheiro et al. 2018 Pinheiro et al. This content is distributed under the terms of the Creative Commons Attribution 4.0 International license.

To investigate the phylogenetic relationship between the TvNlpC/P60 proteins and their homologues, all nine proteins were aligned with a selection of 50 BLASTP-identified sequences, including searches against annotated proteins derived from metagenome data sets. To provide a greater phylogenetic framework than was previously reported (14, 18), published NlpC/P60-candidate LGTs in eukaryotes were considered, including those corresponding to genes from fungi and mites (32) and the free-living protist Acanthamoeba castellanii (33). A conservative alignment, restricted to the well-conserved NlpC/P60 domain (28), was subjected to a combination of complementary phylogenetic analyses, including the use of the simpler empirical homogenous rate matrix-based models reminiscent of published phylogenies (28, 32) (Fig. 1C; see also Table S5). The TvNlpC/P60 proteins did not cluster with those belonging to members of the vaginal microbiota (in contrast to a recent case of LGT reported in T. vaginalis [15, 34]) but formed two distinct clans (Fig. 1C) in all unconstrained analyses (Table S5). Despite weak bootstrap support for some branches (values of <50%), all unconstrained maximum likelihood phylogenetic analyses and the dissimilar indels support the idea that TvNlpC/P60 proteins form two separate groups of sequences (clans A and B) originating from two distinct LGT events (Fig. 1C; see also Fig. S1 and Tables S1 and S5). Two additional observations support this claim. First, analysis of a complex composition mixture model (C20-based model) led to the rejection of the hypothesis of a single LGT event for the origin of TvNlpC/P60 proteins (Table S5). Second, all the tested hypotheses according to which T. vaginalis might have shared the NlpC/P60 LGT event with one eukaryotic lineage or another were rejected per the results of analyses of all models considered (Table S5). In summary, our analyses together indicate that TvNlpC/P60 genes were acquired laterally from bacteria, possibly from two LGT events followed by gene duplications, and independently from the other sampled eukaryotes.

10.1128/mBio.01784-18.8 TABLE S5 Approximate unbiased (AU) tree topological test for hypotheses of TvNlpC/P60 phylogenetic relationships. Tree topologies derived from unconstrained analyses using different amino acid evolutionary models were tested against each other and against tree topologies derived from four constrained analyses testing specific relationships for the TvNlpC/P60 entries, as described in the main text. Download Table S5, PDF file, 0.1 MB. Copyright © 2018 Pinheiro et al. 2018 Pinheiro et al. This content is distributed under the terms of the Creative Commons Attribution 4.0 International license.

### Structures of Trichomonas vaginalis NlpC_A1 and NlpC_A2.

The structures of NlpC_A1 T. vaginalis AG_119910 (TVAG_119910) and NlpC_A2 (TVAG_457240) were determined by X-ray crystallography. Their crystals diffracted to 1.2-Å and 2.3-Å resolution and belonged to the space groups P21221 and P1, respectively (Table S6). The NlpC_A1 structure (PDB identifiers [ID] 6BIM and 6BIO), determined by single isomorphous replacement using a selenomethionine (SeMet)-substituted protein, shows a single molecule in the asymmetric unit with all residues visible in the electron density map. The NlpC_A2 structure (PDB ID 6BIQ), subsequently determined by molecular replacement using NlpC_A1 as a search model, shows four copies in the asymmetric unit with residues 11 to 275 visible in the electron density maps.

10.1128/mBio.01784-18.9 TABLE S6 Data collection and refinement statistics. Values in parentheses represent the highest-resolution shell. Download Table S6, PDF file, 0.2 MB. Copyright © 2018 Pinheiro et al. 2018 Pinheiro et al. This content is distributed under the terms of the Creative Commons Attribution 4.0 International license.

**FIG 2:**
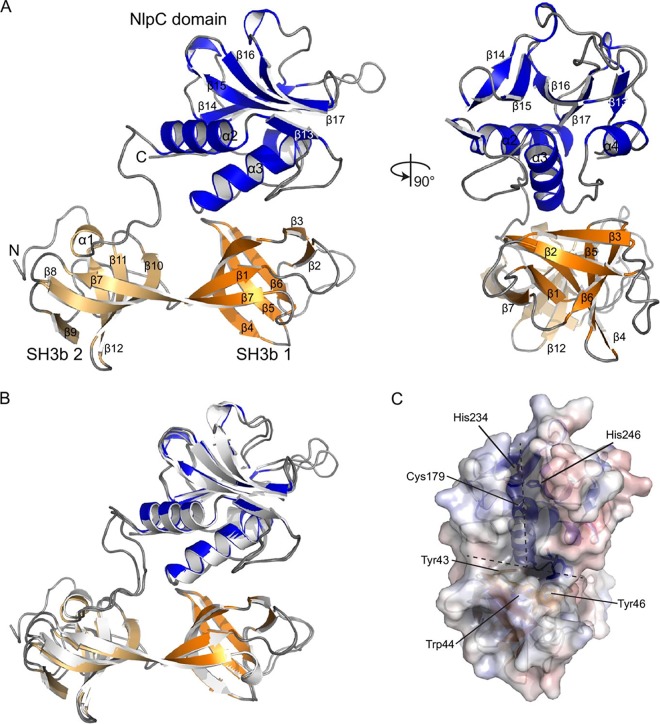
Structures of NlpC_A1 and NlpC_A2. (A) Orthogonal views of a cartoon representation of the structure of NlpC_A1. Secondary structure elements are labeled; N and C termini are indicated. The NlpC domain is colored in blue, with the two SH3b domains shaded in orange. (B) Superposition of the NlpC_A1 and NlpC_A2 structures. The two structures superimpose with an RMSD value of 0.491 Å across all equivalent atoms. (C) Electrostatic surface representation of the NlpC_A1 active-site groove, including a description of the catalytic triad Cys179-His234-His246. The location of the “T”-shaped groove is marked with a dashed line.

NlpC_A1 and NlpC_A2 consist of the C-terminal NlpC/P60 domain preceded by two bacterial SH3 domains (Fig. 2A). As expected, due to the high sequence identity and similarity (90.2% and 96.4%, respectively), the two structures are essentially identical (Fig. 2B), with a root mean square deviation (RMSD) value for all atoms of 0.491 Å (1,379 equivalent atoms). For brevity, the NlpC_A1 structure is detailed and differences with NlpC_A2 are highlighted. The SH3b domains consist of 6 β-strands arranged in a beta barrel formation. They are joined by a shared β-strand (β7) and pack against the NlpC domain in a triangular arrangement. A short linker of ∼10 residues joins the second SH3b domain to the NlpC domain.

The NlpC/P60 domain adopts the classical papain-like fold (35), with a central β-sheet of 5 strands displaying an antiparallel arrangement. Three helices (α2, α3, and α4) are packed against one side of the sheet, forming the interface to the SH3 domains. The similarity to other NlpC proteins allows us to assign the active-site residues of Cys179-His234-His246, present in all TvNlpC/P60 sequences (Fig. S1). The active-site cysteine is located at the N terminus of helix α3, while the two histidines are in strands β15 and β16. The active-site residues sit in a “T”-shaped groove that is bounded by strands β14 and β15 in the NlpC domain, with the cross of the T formed by the β3 strand in the first SH3b domain (Fig. 2C). The groove is open and lacks the obvious regulatory elements that are often present in bacterial endogenous peptidases (36), suggesting that both NlpC_A1 and NlpC_A2 may be unregulated “toxin-like” hydrolases of bacterial PG.

A surface electrostatic calculation (37) demonstrated that the slightly positively charged active-site groove carries two pronounced areas of strong positive charge. The first of the two areas is adjacent to the catalytic cysteine, and the second is at the base of a small pocket at the interface between the NlpC domain and the first SH3b domain bounded by the residues His195, Tyr43, Trp44, Tyr46, Phe193, and Gln184 (Fig. 2C). A single-residue difference in NlpC_A2 is located near the active site where Leu169 in NlpC_A1 corresponds to Trp169 in NlpC_A2. The tryptophan residue narrows the groove around the active-site cysteine in NlpC_A2. The remaining sequence differences, spread across the protein surface, show no obvious cluster of residues that might influence function.

The presence of multiple SH3b domains, while not unusual for NlpC proteins (28), is intriguing. NlpC domains are often accompanied by accessory domains that alter substrate specificity. Previous studies of multidomain NlpC/P60 proteins have shown that the accessory domains can exist in a flexible conformation for recognition of PG (38). Consequently, small-angle X-ray scattering (SAXS) was used to confirm the arrangement of domains within the crystal and that NlpC_A1 and NlpC_A2 are monomeric in solution (Fig. S2). The theoretical scattering from the structural models with SAXS curves was consistent with the arrangement of domains present in the crystal, suggesting that the NlpC domain and SH3b domains pack in a concerted arrangement in solution (Fig. S2) (Table S7).

10.1128/mBio.01784-18.2 FIG S2 Small-angle X-ray scattering analysis of NlpC_A1 and NlpC_A2. (A and B) Scattering data for (A) NlpC_A1 and (B) NlpC_A2. A dilution series was collected for each protein, and the resultant data show no concentration dependence in the scattering profile. Results of Guinier analysis performed for each concentration are shown in the inset. (C and D) The pair distribution function *p*(*r*) for (C) NlpC_A1 and (D) NlpC_A2. (E and F) Results of comparisons of the observed scattering profile with the theoretical scattering from the structures of (E) NlpC_A1 and (F) NlpC_A2. Download FIG S2, PDF file, 0.1 MB. Copyright © 2018 Pinheiro et al. 2018 Pinheiro et al. This content is distributed under the terms of the Creative Commons Attribution 4.0 International license.

10.1128/mBio.01784-18.10 TABLE S7 Small-angle X-ray scattering data analysis. Download Table S7, PDF file, 0.1 MB. Copyright © 2018 Pinheiro et al. 2018 Pinheiro et al. This content is distributed under the terms of the Creative Commons Attribution 4.0 International license.

To further investigate the role of these SH3b domains, we undertook a series of structural similarity searches using the SSM algorithm (39). Searches failed to identify other structures with a similar global domain arrangement. Using the NlpC domain, searches identified only the members of the NlpC superfamily that contain SH3b domains. The Bacillus cereus NlpC/P60 protein (YkfC; PDB ID 3H41) shares the same domain composition, i.e., two SH3b domains at the N terminus followed by the C-terminal NlpC domain. However, alignment of the structures based on the NlpC domains (258 atoms; RMSD value of 0.548 Å) revealed an alternative arrangement of the SH3b domains relative to the NlpC domain. Additionally, the first SH3b domain of YfkC has a large insertion consisting of three α-helices (∼40 residues) which results in the active site of YkfC being more closed (typical of a recycling enzyme [40]) than those of NlpC_A1 and NlpC_A2.

### NlpC_A1 and NlpC_A2 degrade peptidoglycan.

**FIG 3:**
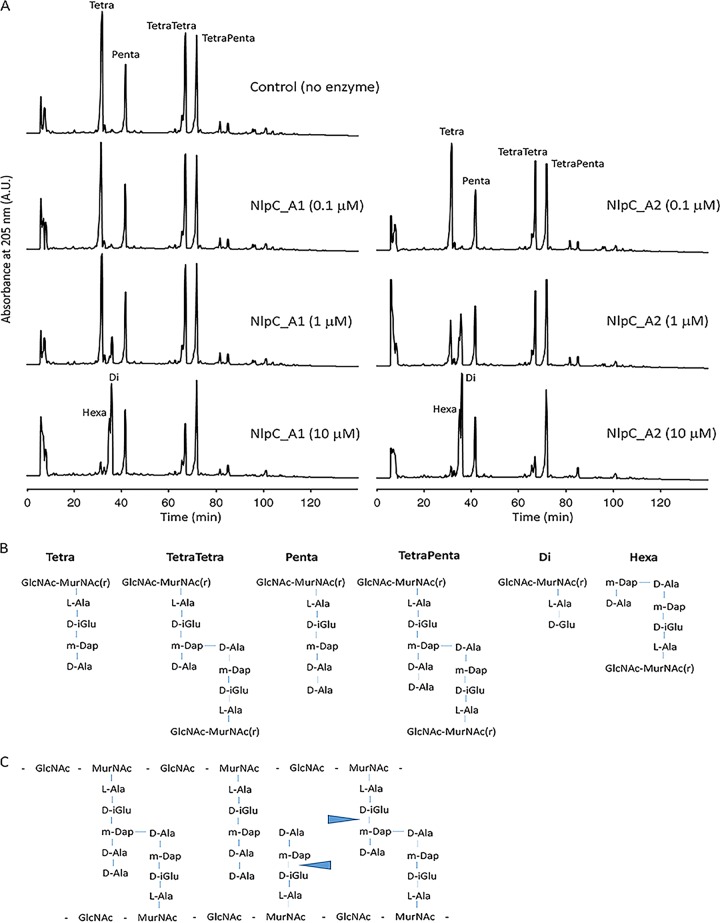
NlpC_A1 and NlpC_A2 are dl-endopeptidases. (A) HPLC chromatograms of E. coli peptidoglycan cleavage assays performed with a control (no enzyme) and with increasing concentrations of NlpC_A1 or NlpC_A2. Peaks of the major muropeptides were assigned by comparison with published literature and are labeled Tetra, Penta, TetraTetra, and TetraPenta. NlpC_A1 and NlpC_A2 produce the muropeptides Di and Hexa. A.U., absorbance units. (B) Structure of the muropeptides labeled on the chromatograms. MurNAc(r), reduced N-acetylmuramic acid; GlcNAc, *N*-acetylglucosamine; L-Ala, l-alanine; d-iGlu, iso–d-glutamic acid; m-Dap, meso-diaminopimelic acid; D-Ala, d-alanine. (C) Schematic diagram of the undigested E. coli peptidoglycan with the indication of the cleavage sites of NlpC_A1 and NlpC_A2 (blue arrows).

We next examined the activity of the recombinant enzymes against PG from Escherichia coli as a model substrate. A mixture consisting of equal amounts of PG from strains MC1061 (rich in tetrapeptides) and CS703-1 (carboxypeptidase mutant, rich in pentapeptides) (41, 42) was incubated with NlpC_A1 or NlpC_A2 or the corresponding predicted inactive versions (catalytic Cys residue replaced by either Ala or Ser), followed by digestion with the muramidase cellosyl and high-performance liquid chromatography (HPLC) analysis of the resulting muropeptides (Fig. 3; see also Fig. S3).

10.1128/mBio.01784-18.3 FIG S3 Mutant NlpC proteins are inactive against E. coli PG. HPLC chromatograms from peptidoglycan cleavage assays performed with NlpC mutant proteins (10 μM) and control assays (no enzyme) are indicated. Peaks were assigned by comparison with published literature. No detectable activity was observed for any of the mutant proteins. Download FIG S3, PDF file, 0.1 MB. Copyright © 2018 Pinheiro et al. 2018 Pinheiro et al. This content is distributed under the terms of the Creative Commons Attribution 4.0 International license.

The control sample showed the expected major monomeric (Tetra and Penta) and dimeric (TetraTetra and TetraPenta) muropeptides (Fig. 3A and B). In the presence of increasing concentrations of NlpC_A1 or NlpC_A2, the Tetra peak decreased and new peaks corresponding to cleavage products appeared, indicating activity against PG (Fig. 3A). These new peaks resulted from the cleavage of the bond between d-isoGlu and *m-*DAP residues (Fig. 3C) and classified both NlpC_A1 and NlpC_A2 as dl-endopeptidases. At the highest concentration of the enzymes, the dimeric TetraTetra was digested whereas the muropeptides with pentapeptides (Penta and TetraPenta) were largely inert. Hence, we concluded that NlpC_A1 and NlpC_A2 are dl-endopeptidases with specificity for the tetrapeptides in peptidoglycan and with greater activity toward monomeric muropeptides. As expected, mutation of the catalytic Cys179 to either Ser or Ala in NlpC_A1 and NlpC_A2 completely abolished their activity toward PG (Fig. S3).

### Expression of endogenous NlpC_A1 and NlpC_A2 genes in Trichomonas vaginalis.

The structures and activities of NlpC_A1 and NlpC_A2 suggest that they have been functionally preserved after LGT, as found in the recent examples of PG-degrading enzymes acquired by other eukaryotes (25, 26). To examine the potential role of these enzymes, we tested if the parasite alters the expression levels of these NlpC genes in the presence of bacteria and if bacterial survival is affected by coculture with the parasite. Bacteria and parasites were coincubated at a 1:10 ratio in a minimal defined medium at 37°C. Samples were taken at 2-, 4-, and 8-h time points for counting of CFUs (colony-forming units) and for reverse transcription and quantitative PCR (RT-qPCR) analysis. The counts of CFUs were used to calculate the bacterial survival as the ratio of CFU values from mixed versus bacterium-alone control cultures.

**FIG 4:**
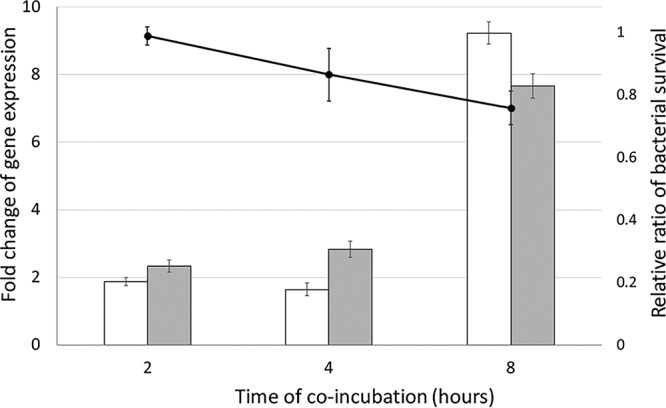
A reduction in E. coli numbers is accompanied by transcriptional upregulation of NlpC_A1 and NlpC_A2 genes in T. vaginalis. E. coli was incubated in the absence or in the presence of T. vaginalis at a ratio of 1 bacterium to 10 protozoan cells for up to 8 h. The CFU counts were used to calculate bacterial survival (line graph following the *y* axis to the right). Bacterial survival was expressed as a ratio of CFU levels in the presence versus absence of T. vaginalis. Simultaneously, T. vaginalis in the absence of bacteria served as the baseline for the RT-qPCR analysis (bar graph following the *y* axis to the left). Relative quantification of NlpC_A1 and NlpC_A2 mRNA abundances (white and gray bars, respectively) was achieved by comparing T. vaginalis levels in the presence versus absence of bacteria for each time point. The HSP70 housekeeping gene was used as a reference, and the *C_T_* method was applied for quantification of relative gene expression levels (see Materials and Methods). The values represent means ± standard deviations (SD) of results from three independent experiments.

We observed that T. vaginalis upregulated expression of both NlpC_A1 and NlpC_A2 in the presence of bacteria. At the latest time point (8 h), transcription of both genes was upregulated 8-fold to 9-fold (Fig. 4). Interestingly, the increased expression of the NlpC genes was accompanied by an ∼20% to 30% reduction in the number of bacteria compared to level seen with the control without parasites. These results show that bacteria triggered the upregulation of *nlpC* genes in T. vaginalis.

### Subcellular localization of NlpC_A1 in Trichomonas vaginalis.

**FIG 5:**
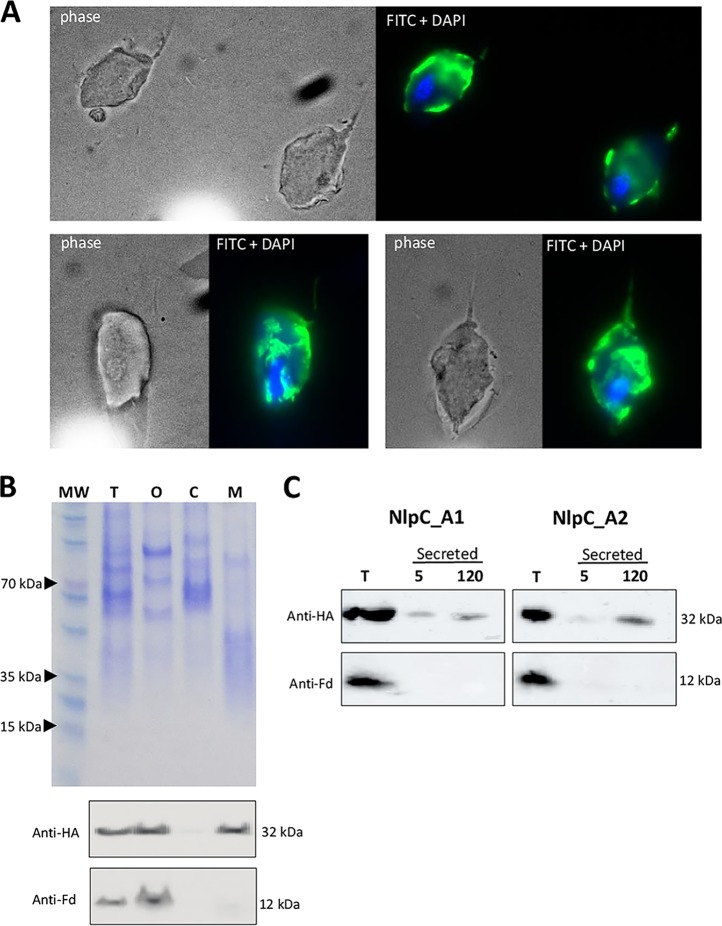
T. vaginalis NlpC_A1 is exported to the cell surface and secreted. The parasite was stably transfected with plasmids expressing HA-tagged NlpC_A1 protein. (A) Images from immunofluorescence microscopy, performed using primary HA-specific and secondary fluorescein isothiocyanate (FITC)-conjugated antibodies, show NlpC_A1 present on the membrane of the protozoan cells. The nucleus was stained with DAPI for reference. Three independent images (total magnification of ×1,000) are shown. (B) Western blot of cell fractionated protein samples. Cell fractions were obtained as T (total, i.e., whole cell with no fractionation), O (organelles, including nuclei), C (cytosol), or M (membrane). Samples were loaded on SDS-PAGE gels at equivalent volumes, along with a molecular weight (MW) marker, for Coomassie staining (top) and Western blotting (bottom). Western blots were probed with primary anti-HA and anti-ferredoxin (anti-Fd) antibodies, as indicated. Although some HA signal was seen on fraction O (indicating unbroken cells), the HA-tagged NlpC_A1 was detected on fraction M, which indicates membrane localization. This fraction is clean with respect to unbroken cells, since anti-Fd antibody gave no signal. (C) Western blots of soluble secreted proteins. Secreted proteins were prepared as previously described (43), and fractions were collected at 5 and 120 min postincubation, as indicated, and increased to volumes equivalent to that of the total cell extract (T). Western blots were probed with primary anti-HA and anti-ferredoxin (anti-Fd) antibodies, as indicated. Contrastingly to the anti-Fd signal, which was detected only from total cells, NplC_A1 (left) was detected in the secreted fraction comparably to NlpC_A2 (right).

While bacterial NlpC/P60 proteins often contain either a SP or a transmembrane region (28), it was unclear if such signatures have been maintained or ameliorated in T. vaginalis NlpC versions (Table S1). NlpC_A2 was recently detected from the T. vaginalis secretome (43). Hence, we next aimed to localize NlpC_A1 in T. vaginalis cells (Fig. 5). A C-terminal hemagglutinin (HA)-tagged NlpC_A1 was expressed in T. vaginalis from a strong constitutive promoter and was detected by immunofluorescence microscopy and Western blotting.

Immunofluorescence microscopy showed strong and consistent surface staining of transfected T. vaginalis cells, suggesting that HA-tagged NlpC_A1 may be located on the plasma membrane (Fig. 5A). The immunostaining of the cell surface was patchy, reaching areas of the flagellar membrane. This was consistent with the cell fractionation assay showing the presence of the HA-tagged NlpC_A1 in the membrane pellet fraction of the Western blot (Fig. 5B). Finally, following a time-dependent secretion assay previously described for T. vaginalis (43), NlpC_A1 was found to be secreted similarly to NlpC_A2. Together, these findings provide experimental evidence that NlpC_A1 is likely to be exported to the cell surface of T. vaginalis and secreted.

### Profound phenotypic changes upon exogenous overexpression of the NlpC_A1 gene.

**FIG 6:**
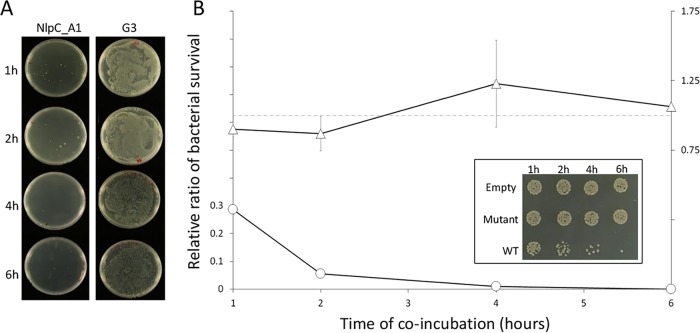
T. vaginalis promotes clearance of E. coli DH5α from mixed cultures in overexpressing NlpC_A1 constitutively. T. vaginalis and E. coli were coincubated in minimal media at different cell ratios. After incubation at 37°C for up to 6 h, cultures were plated on LB agar. (A) T. vaginalis G3 (nontransfected) and T. vaginalis transfected with the NlpC_A1 wild type were incubated with E. coli at a 10:1 ratio. Plates of undiluted cocultures revealed a severe reduction of bacterial growth at as early as 1 h. (B) Stably transfected T. vaginalis expressing no NlpC_A1 protein (Empty), the NlpC_A1 strain mutated at the catalytic residue C179S (Mutant), or the NlpC_A1 wild-type (WT) strain was coincubated with E. coli. A 1:10 protozoan/bacterium cell ratio was used instead. E. coli CFU counts were used to determine bacterial survival in the presence of T. vaginalis expressing mutant (triangles) or WT (circles) NlpC_A1. The inset illustrates the growth of E. coli coincubated with each of the T. vaginalis transfectants and at each time point, as indicated. Undiluted mixed cultures were individually spotted on a LB agar plate. The values represent means ± SD of results from three independent experiments.

T. vaginalis was able to reduce slightly the population of bacteria in mixed cultures when parasites were in 10-fold excess relative to the levels of bacteria (Fig. 4). Next, we tested if overexpressing NlpC_A1 could enhance this phenotype (Fig. 6). With the parasites present in excess, NlpC_A1 T. vaginalis transfectants had virtually eliminated bacteria in the cocultures after just 1 h of coincubation (Fig. 6A). Therefore, the cell ratio was inverted to 10:1 (bacteria/protozoa). In addition, to test if bacterial clearance was dependent on the specific activity of NlpC_A1, we included the transfectant expressing the catalytically inert NlpC_A1 mutant (C179S) along with the empty-plasmid transfectant as a negative control. Bacterial growth and CFU numbers were determined for each time point by spotting and spreading cocultures on agar plates. The obtained data are reported as the relative CFU counts obtained from mixed cultures containing T. vaginalis transfected with either wild-type NlpC_A1 or the mutant NlpC_A1 versus the empty-plasmid T. vaginalis transfectant (Fig. 6B).

A significant drop in bacterial viability in the spot plate assay was observed in the presence of T. vaginalis expressing active NlpC_A1 but not in coculture with T. vaginalis expressing the inactive C179S version or harboring the empty plasmid (Fig. 6B, inset). Quantification of CFU confirmed the drastic loss in bacterial viability when T. vaginalis overexpressed the wild-type, active form of NlpC_A1 (Fig. 6B, line graph). The bacterial counts were reduced by 70% after 1 h, and the bacteria were virtually eliminated after 4 to 6 h. In sharp contrast, T. vaginalis transfected with the catalytically inactive NlpC_A1 (C179S mutant) or empty plasmid produced a constant bacterial CFU ratio of ∼1 throughout the course of the experiment.

**FIG 7:**
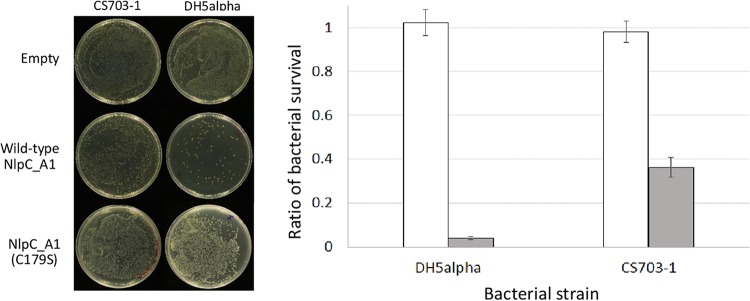
T. vaginalis overexpressing NlpC_A1 is partially impaired with respect to clearing up a pentapeptide-rich peptidoglycan bacterial strain from mixed cultures. T. vaginalis was stably transfected with plasmids expressing no NlpC_A1 protein (Empty), wild-type NlpC_A1, and the catalytically inert NlpC_A1 (C179S). E. coli strains DH5α and CS703-1 were incubated alone or in the presence of each of the transfected T. vaginalis strains at a ratio of four bacterial cells to one protozoan cell for 1 h. (Left) To illustrate the bacterial growth inhibitory effect, the undiluted mixed cultures of transfected T. vaginalis with these strains of E. coli were plated on LB agar. (Right) To measure the levels of bacterial survival, dilutions of the mixed cultures were plated on LB agar and CFU counts were obtained. For each E. coli strain (DH5α and CS703-1), CFU counts were used to calculate bacterial survival in the presence of the T. vaginalis empty strain (white bar) or the T. vaginalis strain expressing wild-type NlpC_A1 (gray bar).

To further demonstrate that the specific activity of NlpC_A1 is responsible for the observed reduction of bacterial viability, T. vaginalis transfectants were incubated with either E. coli strain CS703-1 (42) or strain DH5α, exhibiting a pentapeptide- or tetrapeptide-rich PG, respectively (Fig. 7). We showed previously that recombinant NlpC_A1 was incapable of digesting pentapeptides in PG (Fig. 3). A visual inspection of spread plates after 1 h of coincubation showed a significant reduction of the numbers of bacterial colonies regardless of the strain when T. vaginalis overexpressed the wild-type form of NlpC_A1 (Fig. 7, left). Quantification of CFU showed that T. vaginalis overexpressing the wild-type NlpC_A1 was partially prevented from clearing CS703-1 compared to DH5α by a factor of ∼10 (Fig. 7, right), which is consistent with the biochemical data showing that this enzyme has a preference for digesting tetrapeptides in PG.

## DISCUSSION

We demonstrated that TvNlpC/P60 genes encode dl-endopeptidases that cleave bacterial PG, a function that was preserved after the protozoan *Trichomonas* obtained these genes by LGT. Our phylogenetic analyses indicate that these genes were most likely acquired from bacteria and were duplicated during the evolution of the *Trichomonas* lineage. Although this analysis could not establish the bacterial donor lineage of these LGTs, results of both unconstrained and constrained phylogenetic analyses are in agreement with the idea of two distinct TvNlpC/P60 gene families that possibly originated from two independent LGT events. These analyses further support the hypothesis of independent LGT events in other eukaryotic lineages (22). Importantly, these genes were integrated in the biology of this parasite through amelioration (44). For example, expression of *nlpC_A1* and *nlpC_A2* genes seems to respond to environmental cues found in the vagina. These genes are upregulated under low-glucose conditions (16), and we showed here that they are also upregulated in the presence of bacteria. In addition, the corresponding proteins evolved with functional localization signals, with both NlpC_A1 and NlpC_A2 secreted by T. vaginalis allowing bacteria being targeted at a distance by these PG-degrading enzymes.

Our study showed that NlpC_A1 and NlpC_A2 are likely redundant in structure and function. In terms of structure, they both exhibit a classical papain-like NlpC/P60 domain in addition to two bacterial SH3 domains in a specific tridimensional arrangement. Both structures revealed the presence of an open and highly accessible active site characteristic of a cell-wall degrading toxin (45) and differing from housekeeping NlpC/P60 PG hydrolases of bacteria. In terms of function, they both cleave between d-isoGlu and *m*-DAP residues on monomeric and dimeric peptidoglycan subunits. Notably, neither of them can cleave this bond when the PG substrate is a pentapeptide or tetrapentapeptide. In many bacterial species, including E. coli, the majority of the pentapeptides in the nascent PG are trimmed to tetrapeptides by dd-carboxypeptidases during maturation, resulting in a tetrapeptide-rich peptidoglycan (46). Therefore, T. vaginalis NlpC_A1 and NlpC_A2 are apparently optimized to digest mature bacterial cell walls, consistent with functional enzymes targeting mature bacterial substrates in the native habitat of the parasite.

We observed a profound reduction in the levels of viable bacteria when an exogenous copy of NlpC_A1 gene was constitutively expressed under the control of a strong promoter in T. vaginalis. This enhanced ability to control bacterial population in cocultures, even under conditions of a 10-fold excess of bacteria, was dependent on the activity of this protein since this phenotype was not observed with T. vaginalis expressing catalytically inactive NlpC_A1 (C179S). In accordance with the activity of the recombinant enzyme (i.e., with its being unable to cleave a pentapeptide-rich PG), T. vaginalis was partially impaired with respect to clearing up bacteria that carried a pentapeptide-rich PG. Our study results, however, did not clarify how these enzymes reach the PG on the bacterial cell wall. Purified recombinant NlpC_A1 alone does not have lytic activity against E. coli in the presence of the outer membrane permeabilizer EDTA (data not shown). Therefore, the presence of other factors such as cationic antimicrobial peptides or lysozyme of microbial or human origin (47) might be necessary. Vaginal secretions, for instance, are known to have the highest level of lysozyme among mucosal sites (48).

The PG is a unique polymer that constitutes an essential component of the bacterial cell envelope. There are significant variations in the structure of the cell wall and in the chemical composition of PG among bacterial species. Therefore, further studies are necessary to understand the apparent “redundancy” of the members of the TvNlpC/P60 gene family. PG hydrolytic enzymes may appear redundant in bacteria, but their distinct biochemistries are necessary for proper PG biogenesis (49). We have not yet characterized all members of TvNlpC/P60 gene family and or examined their broad bacterial activity. Following this line, further studies might reveal the potential role of these enzymes in the composition of the vaginal microbiota and in T. vaginalis pathobiology. At this stage, we envisage that T. vaginalis might benefit from the presence of TvNlpC/P60 enzymes by using PG fragments and bacterial metabolites for nutrition and/or by reducing microbial competition in the vagina for its own benefit. Since PG fragments are known ligands of pattern recognition receptors (50, 51), these enzymes may also play a role in immune responses. This study revealed a novel aspect of the biology of T. vaginalis. It was the first to report that a group of genes acquired by LGT enables an extracellular eukaryotic pathogen of the mucosa to control bacterial populations. Further investigations might decipher the role(s) of these LGT-acquired enzymes in the interactions of T. vaginalis with the host and the vaginal microbiota.

## MATERIALS AND METHODS

### General sequence analyses and phylogeny.

The structural organization of the T. vaginalis NlpC/P60 proteins was investigated with InterProScan (52) complemented with SPOCTOPUS (53). In order to compile an alignment of homologues, BLASTP searches at the NCBI were performed against the nr database, RefSeq database, or Env_nr database, with one protein member of each of the two T. vaginalis NlpC/P60 clusters identified at TrichDB (29) (Table S1). A more sensitive DELTA-BLAST search against the RefSeq database was performed to identify potential homologues in other eukaryotes. To investigate the phylogenetic position of the nine T. vaginalis NlpC/P60 proteins, selections of top BLASTP hits were combined to maximize taxonomic representation. This alignment was trimmed with trimAl (option: gappyout) (54) to 104 residues, ensuring that a conservative selection of well-aligned residues was used for the phylogenetic inference. The alignment was analyzed with IQ-TREE (55, 56) in order to establish the best-fitting model based on single-amino-acid replacement matrices for the NlpC/P60 protein alignment, which was LG + G4 + I. Additional protein mixture evolutionary models were also considered (LG4X [57] and C20 model [58]) as they are more reliable in extracting phylogenetic signal from divergent sequences. Unconstrained phylogenetic analyses were performed with IQ-TREE with either unique exchange rate matrix-based models (LG + G4 + I or LG + G4 + I + F) or empirical protein mixture models (LG4X + R + F or C20 + R). Constrained analyses forcing specific relationships, including (i) all nine TvNlpC/P60 entries being monophyletic or TvNlpC/P60 being monophyletic with sequences of (ii) two fungal genera (*Aspergillus* and *Metarhizium*), (iii) the amoeba (*Acanthamoeba*), or (iv) the parasitic worm (Trichuris trichiura), were performed with IQ-TREE, as were approximate unbiased (AU) tree topology tests, currently the most appropriate tests for comparisons of multiple trees (59). The maximum likelihood tree (model LG + G4 + I) was edited using iTOL (60) to generate Fig. 1B. The alien index (AI) values for the TvNlpC/P60 genes were calculated as described previously by Rancurel et al. (30) (Table S1). The GC% profiles of TvNlpC/P60 genomic scaffolds and TvNlpC/P60 open reading frames (ORFs) were analyzed with the GC-profile server (61) and CAIcal server (62), respectively. All values are listed for each of the scaffolds/genes in Table S1.

### Microbial cultures and coincubation assay.

Escherichia coli strains BL21(DE3) and DH5α (Invitrogen) and MC1061 and CS703-1 (42) were grown in Luria-Bertani (LB) media with agitation at 37°C. Trichomonas vaginalis reference strain G3 was cultured in TYM medium (63) supplemented with 10% horse serum, 10 U/ml penicillin, and 10 μg/ml streptomycin (Invitrogen) at 37°C with no agitation. For the coincubation assay, T. vaginalis cells grown in the absence of antibiotics were counted under a hemocytometer and viability was assessed. The number and viability of E. coli cells were assessed by flow cytometry as previously described (64). Microbial cultures showing at least 95% viability were spun down, washed, and resuspended in antibiotic-free and keratinocyte serum-free media (K-SFM; Invitrogen). T. vaginalis (5 × 10^5^ cells/ml) was mixed with bacteria in a 12- or 24-well tissue culture plate, in a volume of 0.5 or 1.0 ml, respectively, and incubated at 37 °C. The cell ratio (bacterium?protozoan) and time of incubation are indicated for each experiment, performed in triplicate. As controls, incubations of T. vaginalis and bacteria alone were performed in parallel. At the end of the assay, undiluted and diluted cocultures in sterile water were respectively spotted or spread on LB agar plates, an arrangement that is selective for E. coli. Spread plates were photographed, and CFU counts were obtained.

### Reverse transcription and quantitative PCR (RT-qPCR).

RNA was obtained from the coincubation assay cultures containing either T. vaginalis with E. coli or T. vaginalis alone by the use of TRIzol reagent (Invitrogen). Total RNA was treated with DNase I (Ambion) and cleaned with a minikit and RNeasy MinElute (Qiagen). RNA (5 μg) was reverse transcribed with oligo(dT) primer and Superscript reverse transcriptase (RT) III (Invitrogen). To ensure that the RNA samples were free of DNA, reaction mixtures that omitted the reverse transcriptase (−RT) were included. Quantitative real-time PCR (qPCR) was carried out with the following primers after the specificity and efficiency data for each pair of primers were validated (per the 7900 HT- Realtime instructions; Applied Biosystems). Primers targeting T. vaginalis HSP70 gene TVAG_237140 (forward, ACACAGGCGAGAGACTCGTT; reverse, TCTTTGACCCAAGCATCTCC) were used for normalization. The primers targeting T. vaginalis NlpC_A1 (TVAG_119910) were TCACAATTCCAACCCAATCTG (forward) and CTCCGTCATTTGCACCATCT (reverse). The primers targeting T. vaginalis NlpC_A2 (TVAG_457240) were TAAGACCAAGCTTGGCTGC (forward) and TTCCGACATACATTCCGAC (reverse). qPCR reactions were carried out in triplicate with 10 ng of cDNA (or −RT as the control), a 200 nM concentration of each primer, and PowerUP SYBR green Master Mix (Thermo Fisher). The threshold cycle (*C_T_*) method was applied for relative gene expression analysis in which expression data were normalized, and the results were analyzed using the SDS 2.3 and RQ Manager 1.2 applications (Applied Biosystems). Subsequent statistical analyses were then carried out using the Relative Expression Software Tool (REST) (65).

### Plasmids, PCR, and DNA cloning.

For protein expression in T. vaginalis, the coding sequence of NlpC_A1 was PCR amplified and cloned in the MasterNeo plasmid via the use of NdeI and Asp718 restriction sites (66), providing a strong constitutive promoter and a double-hemagglutinin (double-HA) tag at the protein C terminus. Transfection of T. vaginalis was achieved using a GenePulser Xcell electroporator (Bio-Rad), as previously described (66). A MasterNeo empty plasmid, containing no exogenous gene other than the selectable markers, was transfected as a control. For protein expression in E. coli, coding sequences of NlpC_A1 and NlpC_A2 were (conventionally [via heat shock]) PCR amplified and cloned in the pET47b plasmid (Novagen) using a ligation-independent strategy (67). Site-direct mutagenesis of NlpC_A1 and NlpC_A2, by means of a single substitution of cysteine-179 to either serine or alanine, was achieved by inverse PCR using either the MasterNeo plasmid or the pET47b plasmid as a DNA template. PCRs were performed with high-fidelity Phusion polymerase (Thermo Fisher), and PCR-derived insertions were fully sequenced from their plasmids.

### Expression and purification of recombinant NlpC_A1 and NlpC_A2.

Overnight 5-ml cultures of E. coli BL21(DE3) transformed with NlpC_A1 or NlpC_A2 expression plasmid pET47b were inoculated into 750 ml of LB (25 μg/ml kanamycin) and incubated until an optical density at 600 nm (OD_600_) of 0.4 to 0.6 was reached. After cooling at 18°C for 30 min was performed, protein expression was induced by the use of 0.5 mM IPTG (isopropyl-β-d-thiogalactopyranoside) and expression proceeded at that temperature. Cells were harvested by centrifugation and resuspended in 20 mM Tris-HCl (pH 7.8) and frozen until required or in lysis buffer {20 mM Tris-HCl [pH 7.8], 300 mM NaCl, 0.5 mM TCEP [Tris(2-carboxyethyl)phosphine hydrochloride], 20 mM imidazole, 10% glycerol}. The lysate was cleared by centrifugation (14,000 × *g*, 30 min, 4°C) by the use of a Constant Systems cell disruptor. The His-tagged proteins were purified by immobilized-metal affinity chromatography and eluted using a step gradient in a reaction mixture containing 20 mM Tris-HCl (pH 7.8), 300 mM NaCl, 0.5 mM TCEP, and 300 mM imidazole. NlpC-containing fractions were pooled and dialyzed overnight against a reaction mixture containing 10 mM Tris-HCl, 25 mM NaCl, and 0.1 mM TCEP with 3C protease for tag removal. NlpC proteins were further purified by anion-exchange chromatography (6-ml Resource Q column) and eluted on a gradient of 0 to 0.5 M NaCl. Size exclusion chromatography (Superdex 75 16/60 equilibrated in a reaction mixture containing 10 mM Tris-HCl [pH 7.8], 150 mM NaCl, and 0.1 mM TCEP) was applied as a final purification step. Protein concentrations were determined by UV/visible (UV/Vis) spectroscopy using the following theoretical masses (extinction coefficients): NlpC_A1, 31,415 Da (ε_280nm_, 63,510 M^−1^cm^−1^); NlpC_A2, 31,394 Da (ε_280nm_, 66,155 M^−1^cm^−1^). For the selenomethionine (SeMet)-substituted NlpC_A1, expression was achieved in PASM-505 media using the inhibition method (68) and the protein was purified as described above.

### Protein structure determination of NlpC_A1 and NlpC_A2.

Crystallization of NlpC_A1 (16 mg/ml) and NlpC_A2 (14.5 mg/ml) was undertaken by sitting the drop vapor diffusion method using 96-well Intelli-Plate crystallization trays, the Morpheus screen (69), and our in-house robot screens (70). Crystals of NlpC_A1 were grown under Morpheus screen condition A1 (10% polyethylene glycol [PEG] 20000, 20% PEG methyl ether 550 [550 MME], 0.06 M divalents, 0.1 M imidazole–MES [morpholineethanesulfonic acid] [pH 6.5]). Crystals of NlpC_A2 grew in 0.2 M ammonium fluoride–20% PEG 3350. For data collection, NlpC_A1 crystals were individually frozen by being plunged into liquid nitrogen. NlpC_A2 crystals were transferred to a cryoprotectant consisting of reservoir solution supplemented with 20% glycerol and were frozen as described above. X-ray diffraction data were collected using our in-house X-ray generator consisting of a Rigaku Micromax-007HF rotating anode (CuKα) and a MAR345 detector. NlpC_A1 crystals were also sent to the Australian Synchrotron facility for collection of high-resolution data. The structure of NlpC_A1 was determined by single isomorphous replacement using Native and SeMet data collected from our in-house X-ray suite. Selenium sites were identified using the SHELXC/D/E pipeline (71) within the CCP4 program suite (72). Phases were then used to build an initial model in PHENIX (73) followed by iterative rounds of model building and refinement in Coot (74) and PHENIX (73). The structure of NlpC_A2 was determined by molecular replacement using PHASER (75) with NlpC_A1 as a search model.

### Enzymatic activity of recombinant NlpC_A1 and NlpC_A2.

Equal quantities (0.5 mg/ml) of purified PG from E. coli strains MC1061 (41) and CS703-1 (42) were mixed for the enzymatic assays. NlpC_A1 or NlpC_A2 was incubated at various concentrations (0.1, 1.0, and 10 μM) with the E. coli PG mixture in 20 mM Tris-HCl (pH 7.5)–150 mM NaCl for 4 h at 37°C using a Thermomixer at 750 rpm. A control sample received either no enzyme or one of the inactive mutants NlpC_A1(C179A), NlpC_A1(C179S), NlpC_A2(C179A), and NlpC_A2(C179S) at the highest concentration (10 μM). The reaction was stopped by the addition of a 1/4 volume of 80 mM sodium phosphate (pH 4.8) and incubation at 100°C for 5 min. The samples were incubated overnight with 10 μg of cellosyl (Hoechst, Frankfurt am Main, Germany) at 37°C on a Thermomixer at 750 rpm. Following this, samples were incubated at 100°C for 10 min and centrifuged at room temperature for 15 min at 16,000 × *g*. The muropeptides present in the supernatant were reduced with sodium borohydride and separated by HPLC as described previously (76, 77).

### Subcellular localization of NlpC_A1.

Subcellular localization of the HA-tagged NlpC_A1 was assessed in the transfected T. vaginalis strain by indirect immunofluorescence assay (IFA) and cell fractionation. For the IFA, the mouse anti-HA primary antibody (Covance) and the Alexa Fluor-conjugated secondary antibody (Invitrogen) were used at 1:1,000 and 1:5,000 dilutions, respectively, as described previously (78). Cells were spotted on slides with ProLong Gold antifade reagent containing DAPI (4′,6-diamidino-2-phenylindole) (Invitrogen) and imaged under a Nikon Ni-U microscope equipped with a Spot Pursuit Slider camera and corresponding software. Nontransfected T. vaginalis cells (negative control) did not produce any detectable signal above background. A cell fractionation protocol (79) was applied to T. vaginalis where ∼10^8^ T. vaginalis cells were burst by 5 to 10 passages through a 25-gauge (25G) needle syringe in the presence of protease inhibitor cocktail (complete mini; Roche). After several steps of differential-speed centrifugation, protein fractions corresponding to organelles (including nuclei), cytosol, and cell membrane were obtained (79). In addition, secretion fractions were prepared as previously described (43) as well as whole-cell protein extracts. Protein fractions brought up to equivalent volumes were analyzed by the use of SDS-PAGE gels stained with Coomassie blue or were blotted to polyvinylidene difluoride (PVDF) membranes. Western blots were probed with either the anti-HA antibody as described above or a rabbit polyclonal anti-ferredoxin antibody (kindly donated by Patricia Johnson, University of California, Los Angeles [UCLA]). Host-specific secondary antibodies conjugated to horseradish peroxidase were used for chemiluminescent detection (Thermo Fisher Scientific) with a Fuji LAS-4000 imager.
