# Supplementary material for: The Protozoan Trichomonas vaginalis Targets Bacteria with Laterally Acquired NlpC/P60 Peptidoglycan Hydrolases
Source: mBio. 2018 Dec 11;9(6):e01784-18. doi: 10.1128/mBio.01784-18 (PMC6299479; doi:10.1128/mBio.01784-18)
Supplement: FIG S1 [file mbo006184213sf1.pdf]

A

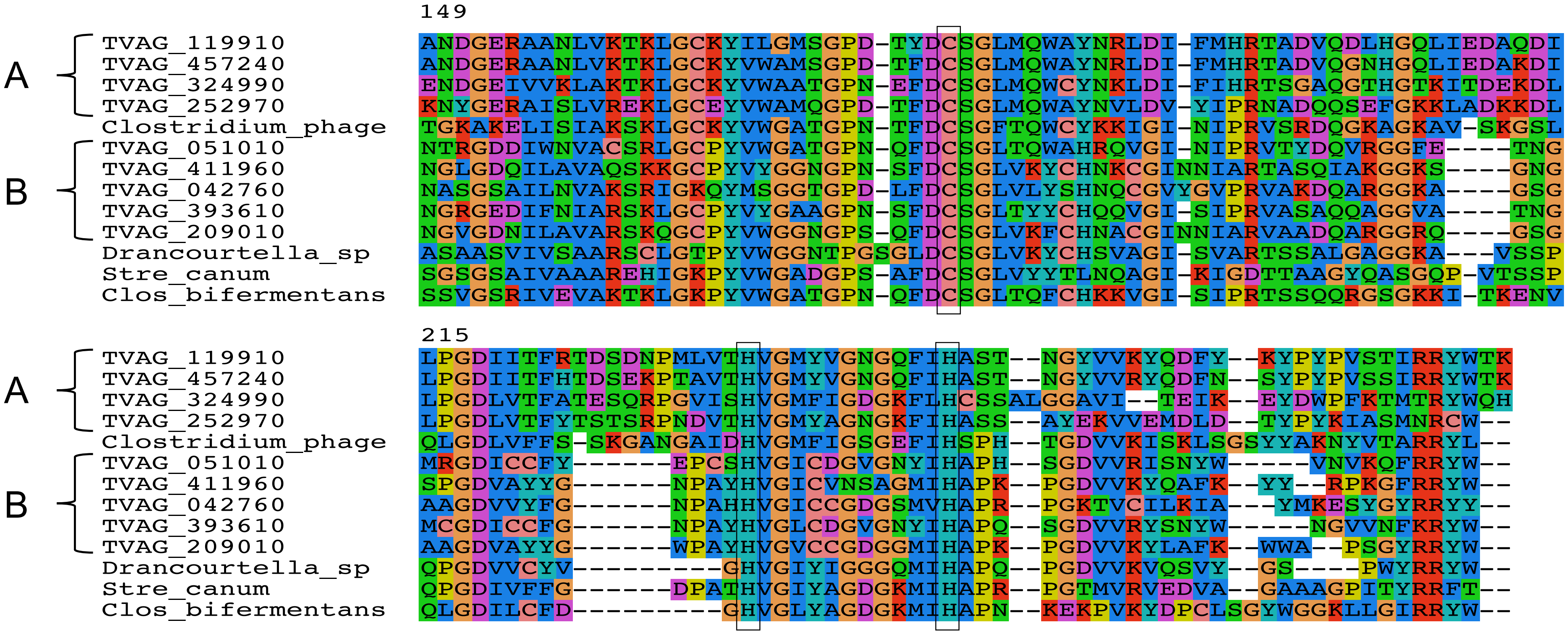

149
A
B
TVAG_119910 ANDGERAANLVKTKLGCKYILGMSGPD-TYDCSGLMQWAYNRLDI-FMHRTADVQDLHGQLIEDAQDI
TVAG_457240 ANDGERAANLVKTKLGCKYVWAMSGPD-TFDCSGLMQWAYNRLDI-FMHRTADVQGNHGQLIEDAKDI
TVAG_324990 ENDGEIVVKLAKTKLGCKYVWAATGPN-EFDCSGLMQWCYNKLDI-FIHRTSGAQGTHGTKITDEKDL
TVAG_252970 KNYGERAISLVREKLGCEYVWAMQGPD-TFDCSGLMQWAYNVLDV-YIPRNADQQSEFGKKLADKKDL
Clostridium_phage TGKAKELISIAKSKLGCKYVWGATGPN-TFDCSGFTQWCYKKIGI-NIPRVSRDQGKAGKAV-SKGSL
TVAG_051010 NTRGDDIWNVACSRLGCPYVWGATGPN-QFDCSGLTQWAHRQVGI-NIPRVTYDQVRGGFE----TNG
TVAG_411960 NGLGDQILAVAQSKKGCPYVYGGNGPN-SFDCSGLVKYCHNKCGINNIARTASQIAKGGKS----GNG
TVAG_042760 NASGSAILNVAKSRIGKQYMSGGTGPD-LFDCSGLVLYSHNQCGVYGVPRVAKDQARGGKA----GSG
TVAG_393610 NGRGEDIFNIARSKLGCPYVYGAAGPN-SFDCSGLTYYCHQQVGI-SIPRVASAQQAGGVA----TNG
TVAG_209010 NGVGDNILAVARSKQGCPYVWGGNGPS-QFDCSGLVKFCHNACGINNIARVAADQARGGRQ----GSG
Drancourtella_sp ASAASVIVSAARSCLGTPYVWGGNTPGSGLDCSGLVKYCHSVAGI-SVARTSSALGAGGKA---VSSP
Stre_canum SGSGSAIVAAAREHIGKPYVWGADGPS-AFDCSGLVYYTLNQAGI-KIGDTTAAGYQASGQP-VTSSP
Clos_bifermentans SSVGSRIVEVAKTKLGKPYVWGATGPN-QFDCSGLTQFCHKKVGI-SIPRTSSQQRGSGKKI-TKENV
215
A
B
TVAG_119910 LPGDIITFRTDSDNPMLVTHVGMYVGNGQFIHAST--NGYVVKYQDFY--KYPYPVSTIRRYWTK
TVAG_457240 LPGDIITFHTDSEKPTAVTHVGMYVGNGQFIHAST--NGYVVRYQDFN--SYPYPVSSIRRYWTK
TVAG_324990 LPGDLVTFATESQRPGVISHVGMFIGDGKFLHCSSALGGAVI--TEIK--EYDWPFKTMTRYWQH
TVAG_252970 LPGDLVTFYTSTSRPNDVTHVGMYAGNGKFIHASS--AYEKVVEMDLD--TYPYKIASMNRCW--
Clostridium_phage QLGDLVFFS-SKGANGAIDHVGMFIGSGEFIHSPH--TGDVVKISKLSGSYYAKNYVTARRYL--
TVAG_051010 MRGDICCFY------EPCSHVGICDGVGNYIHAPH--SGDVVRISNYW-----VNVKQFRRYW--
TVAG_411960 SPGDVAYYG------NPAYHVGICVNSAGMIHAPK--PGDVVKYQAFK--YY--RPKGFRRYW--
TVAG_042760 AAGDVVYFG------NPAHHVGICCGDGSMVHAPR--PGKTVCILKIA---YMKESYGYRRYY--
TVAG_393610 MCGDICCFG------NPAYHVGLCDGVGNYIHAPQ--SGDVVRYSNYW-----NGVVNFKRYW--
TVAG_209010 AAGDVAYYG------WPAYHVGVCCGDGGMIHAPK--PGDVVKYLAFK--WWA--PSGYRRYW--
Drancourtella_sp QPGDVVCYV---------GHVGIYIGGGQMIHAPQ--PGDVVKVQSVY--GS----PWYRRYW--
Stre_canum QPGDIVFFG------DPATHVGIYAGDGKMIHAPR--PGTMVRVEDVA--GAAAGPITYRRFT--
Clos_bifermentans QLGDILCFD---------GHVGLYAGDGKMIHAPN--KEKPVKYDPCLSGYWGGKLLGIRRYW--

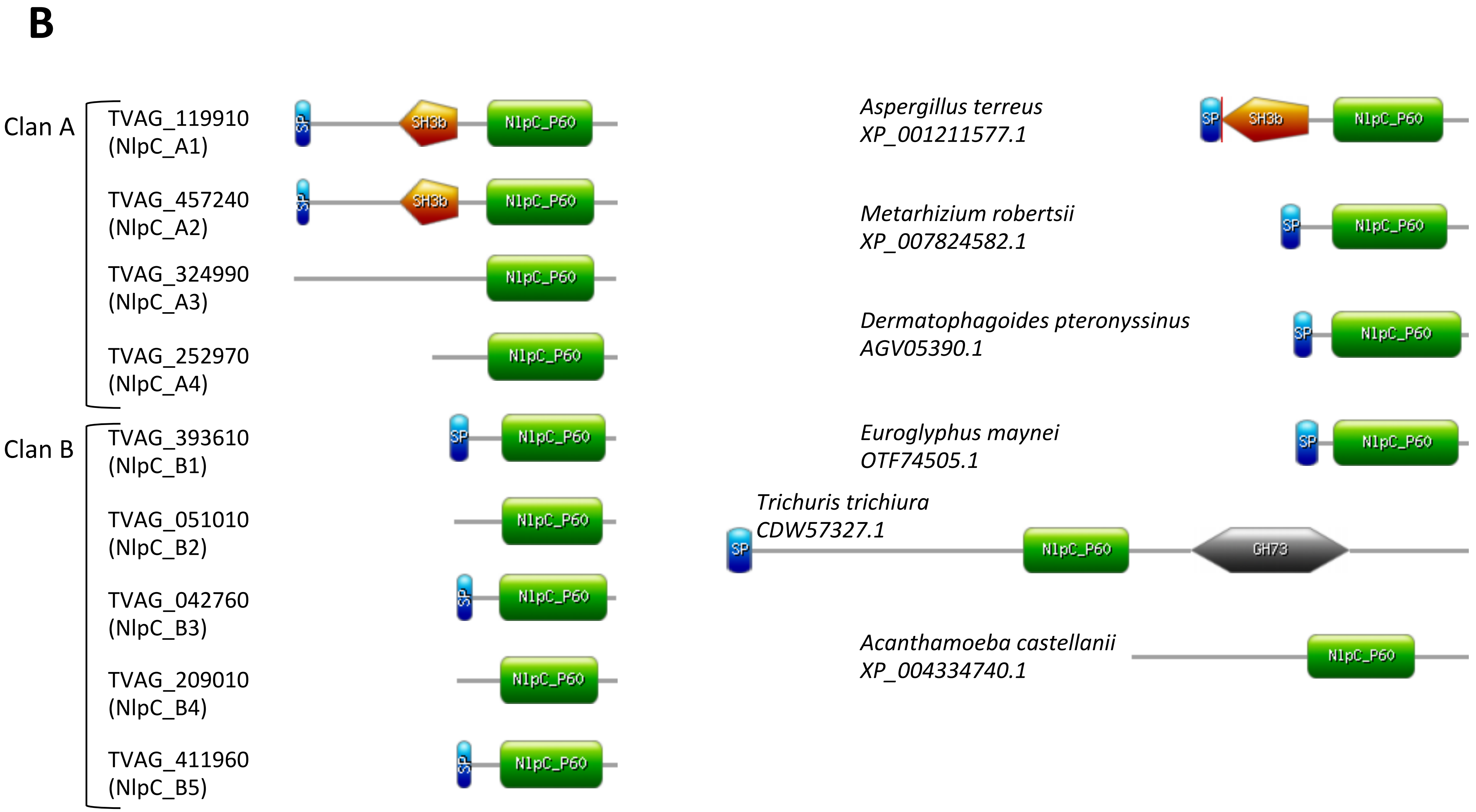

B
Clan A
TVAG_119910
(NlpC_A1)
TVAG_457240
(NlpC_A2)
TVAG_324990
(NlpC_A3)
TVAG_252970
(NlpC_A4)
Clan B
TVAG_393610
(NlpC_B1)
TVAG_051010
(NlpC_B2)
TVAG_042760
(NlpC_B3)
TVAG_209010
(NlpC_B4)
TVAG_411960
(NlpC_B5)
Aspergillus terreus
XP_001211577.1
Metarhizium robertsii
XP_007824582.1
Dermatophagoides pteronyssinus
AGV05390.1
Euroglyphus maynei
OTF74505.1
Trichuris trichiura
CDW57327.1
Acanthamoeba castellanii
XP_004334740.1
SP
SH3b
NlpC_P60
GH73
