# Supplementary material for: The Protozoan Trichomonas vaginalis Targets Bacteria with Laterally Acquired NlpC/P60 Peptidoglycan Hydrolases
Source: mBio. 2018 Dec 11;9(6):e01784-18. doi: 10.1128/mBio.01784-18 (PMC6299479; doi:10.1128/mBio.01784-18)
Supplement: TABLE S1 [file mbo006184213st1.pdf]

TABLE S1A An overview of *T. vaginalis* NlpC/P60 genes, expression data and protein features.

Gene and protein features																
**Number and Locus**	**RefSeq protein accession**	**Scaffold and Gene Location**	**Size of scaffold (bp)**	**Alien index**	**Segmentation point (bp)**	**Scaffold/segments GC%[1]***	**GC%, NlpC/P60 scaffold**	**GC%, NlpC/P60 ORF**	**GC% codons 1-2-3**	**CAI value**	**BlastP top hit with listed query**	**Protein length**	**SP prediction (SignalP/Phobius/Spoctopus)**	**Ortholog group TrichDB**	**Annotation**	**Domains - InterProScan output**
[SD]NlpC_A1 TVAG_119910	XP_001276902	DS113177:259,010..259,837(+)	584,929	24.9	568,529	31.9**; 36.1	31.9	38.5	46.6, 38.6, 29.5	0.544	QUERY	275	No/No/Yes	OG5_140290	Clan CA, family C40, NlpC/P60 superfamily cysteine peptidase	PF08239 Bacterial SH3 domain [90-139] - PF00877 NlpC/P60 family [165-264]
[SD]NlpC_A2 TVAG_457240	XP_001583075	DS113186:279,353..280,222(-)	405,446	22.6	28,559	34.3; 46.1; 31.5**	31.5	38.4	47.3, 39.4, 27.3	0.539	XP_001276902	275	No/No/Yes	OG5_140290	Clan CA, family C40, NlpC/P60 superfamily cysteine peptidase	PF08239 Bacterial SH3 domain[90-139] - PF00877 NlpC/P60 family [164-255]
NlpC_A3 TVAG_324990	XP_001330233	DS113569:37,077..37,913(+)	79,056	24.8	none	32	32	35.5	46.4, 35.2, 23.6	0.551	XP_001276902	277	No/No/No	OG5_140290	Clan CA, family C40, NlpC/P60 superfamily cysteine peptidase	PF00877 NlpC/P60 family [166-258] ; with divergent PF08239 Bacterial SH3 domain, see alignment
NlpC_A4 TVAG_252970	XP_001314869	DS113529:25,222..25,744(+)	84,867	13.8	none	32.6	32.6	40.6	47.9, 38.4, 34.9	0.550	XP_001276902	155	No/No/No	OG5_140290	Clan CA, family C40, NlpC/P60 superfamily cysteine peptidase	PF00877 NlpC/P60 family [47-143]
NlpC_B1 TVAG_393610	XP_001326856	DS113268:173,688..174,113(+)	177,036	29.6	148,091	31.4; 36.4**	36.4	44.1	56.2, 48.2, 28.5	0.533	QUERY	141	Yes/Yes/Yes	OG5_135136	Clan CA, family C40, NlpC/P60 superfamily cysteine peptidase	PF00877 NlpC/P60 family [45-131]
NlpC_B2 TVAG_051010	XP_001321089	DS113369:79,711..80,124(-)	122,255	24.1	118,327	31.9**; 43.4	31.9	49.5	56.2, 45.4, 46.9	0.493	XP_001326856	137	No/No/No	OG5_135136	Clan CA, family C40, NlpC/P60 superfamily cysteine peptidase	PF00877 NlpC/P60 family [41-125]
NlpC_B3 TVAG_042760	XP_001305907	DS113937:20,813..21,226(+)	43,359	19.8	847; 31,157; 31,817; 32,689; 33,103	38.5; 30.1**; 34.6; 48.6; 22.5; 36.21	30.1	50.2	56.4, 51.9, 45.1	0.487	XP_001326856	137	No/Yes/No	OG5_135136	Clan CA, family C40, NlpC/P60 superfamily cysteine peptidase	PF00877 NlpC/P60 family [38-129]
NlpC_B4 TVAG_209010	XP_001327845	DS113253:94,021..94,471(+)	190,068	16.8	none	32	32	51.0	58.6, 53.1, 41.4	0.483	XP_001326856	135	No/No/No	OG5_135136	Clan CA, family C40, NlpC/P60 superfamily cysteine peptidase	PF00877 NlpC/P60 family [45-131]
NlpC_B5 TVAG_411960	XP_001310764	DS113699:60,805..61,212(-)	63,570	23.9	20,687; 34,859; 57,745; 61,289	30.3; 36.2; 31.8; 40.9**; 25.6	40.9	45.1	51.5, 47.0, 37.9	0.523	XP_001326856	135	No/No/Yes	OG5_135136	Clan CA, family C40, NlpC/P60 superfamily cysteine peptidase	PF00877 NlpC/P60 family [37-122]

Gene expression data (transcriptomics) as previously reported (1, 2)																	
	Growth conditions from Gould et al. (2013)(1)[a]											Growth conditions from Huang et al. (2014)(2)[b]					
**Number and Locus tag**	**An0**	**AnOx5**	**AnOx30**	**AnOx120**	**Ad0**	**AdInf5**	**AdInf30**	**AdInf120**	**AdOxInf5**	**AnOxInf30**	**AdOxInf120**	**1%_12h**	**GR_12h**	**GR_24h**	**GR_36h**	**Mean expression by Gould et al (1)**	**Mean expression by Huang et al (2)**
[SD]NlpC_A1 TVAG_119910	150	101.76	81.86	90.92	992.58	79.99	149.47	145.13	348.49	196.09	247.92	33.60	55.10	64.90	77.80	234.93	57.80
[SD]NlpC_A2 TVAG_457240	3238	2789.82	2342.59	3536.98	9818.35	976.12	1143.72	2601.2	4275.92	4330.52	5786.78	53.80	49.20	71.90	94.30	3712.73	67.30
NlpC_A3 TVAG_324990	16	19.86	28.97	22.36	94.44	13.75	48.7	33.49	43.83	42.54	48.61	14.40	14.30	17.00	4.70	37.50	12.60
NlpC_A4 TVAG_252970	146	178.71	141.06	147.56	1134.24	116.23	172.99	188.55	562.28	480.36	474.45	0.10	0.20	0.00	0.00	340.22	0.10
NlpC_B1 TVAG_393610	8	18.62	8.82	10.43	61.11	8.75	11.76	9.92	28.86	19.71	40.83	0.30	0.00	0.00	0.00	20.62	0.10
NlpC_B2 TVAG_051010	14	17.37	10.08	31.3	80.55	18.75	13.44	38.45	14.97	19.71	17.5	0.10	0.40	0.00	2.00	25.10	0.60

NlpC_B3<br>TVAG_042760	1	0	1.26	5.96	3.7	8.75	33.59	22.33	1.07	3.11	1.94	0.10	0.40	1.10	22.50	7.52	6.00
NlpC_B4<br>TVAG_209010	8319	6660.58	7740.63	5787.65	893.5	11051.05	11492.67	8658.27	6354.01	5563.07	3048.95	115.90	694.00	724.00	1005.30	6869.94	634.80
NlpC_B5<br>TVAG_411960	8	1.24	5.04	5.96	2.78	5	6.72	4.96	4.28	4.15	1.94	0.40	2.00	1.00	4.90	4.55	2.10

[1]Two or more values indicate the presence of a segmentation points as estabished by GC-profile previously (3).

*The overal mean GC% for the T. vaginalis G3 genome sequence is 32.7% and for protein coding genes 35.5%.

**In the presence of segmentation points the segment that encodes the TvNlpC_P60 gene is indicated.

[SD]Structural Data available in this study.

[a]An0: anaerobically grown parasites
An0x (5, 30, 120 min): parasite exposed to oxygen stress
AdOxInf (5, 30, 120 min): parasites exposed to oxygen stress and to vaginal epithelial cells
Ad0: trophozoites adapted to 15% CO_2
AdInf (5, 30, 120 min): parasites adapted to 15% CO_2 exposed to vaginal epithelial cells

[b]1% Glucose 12 hrs; Glucose restriction (GR) for 12 hrs, 24 hrs and 36 hrs

References

(1). Gould SB, Woehle C, Kusdian G, Landan G, Tachezy J, Zimorski V, Martin WF. 2013. Deep sequencing of Trichomonas vaginalis during the early infection of vaginal epithelial cells and amoeboid transition. Int J Parasitol 43:707–719.
(2). Huang K, Chen Y-YM, Fang Y, Cheng W-H, Cheng C, Chen Y-C, Wu TE, Ku F, Chen S, Lin R, Tang P. 2014. Adaptive responses to glucose restriction enhance cell survival, antioxidant capability, and autophagy of the protozoan parasite Trichomonas vaginalis. Biochim Biophys Acta - Gen
(3). Gao F, Zhang CT. 2006. GC-Profile: A web-based tool for visualizing and analyzing the variation of GC content in genomic sequences. Nucleic Acids Res 34:686–691.

S1B Table. Alien index calculations

Locus Tag	Annotation	RefSeq accession	E-score best EUK hit*	E-score best PROK hit**	Alien Index (AI) value	References*
TVAG_119910	NlpC/P60 --- NlpC_A1	XP_001276902	2.00E-14	3.00E-25	24.9	This study and (1,2)
TVAG_457240	NlpC/P60 --- NlpC_A2	XP_001583075	2.00E-17	3.00E-27	22.6	This study and (1,2)
TVAG_324990	NlpC/P60 --- NlpC_A3	XP_001330233	4.00E-15	7.00E-26	24.8	This study and (1,2)
TVAG_252970	NlpC/P60 --- NlpC_A4	XP_001314869	2.00E-21	2.00E-27	13.8	This study and (1,2)
TVAG_393610	NlpC/P60 --- NlpC_B1	XP_001326856	5.00E-16	7.00E-29	29.6	This study and (1,2)
TVAG_051010	NlpC/P60 --- NlpC_B2	XP_001321089	3.00E-21	1.00E-31	24.1	This study and (1,2)
TVAG_042760	NlpC/P60 --- NlpC_B3	XP_001305907	2.00E-15	5.00E-24	19.8	This study and (1,2)
TVAG_209010	NlpC/P60 --- NlpC_B4	XP_001327845	2.00E-15	1.00E-22	16.8	This study and (1,2)
TVAG_411960	NlpC/P60 --- NlpC_B5	XP_001310764	5.00E-14	2.00E-24	23.9	This study and (1,2)
TVAG_203590	Threonine synthase family protein	XP_001316278	1.00E-41	2.00E-127	197.3	(1,2)
TVAG_243650	Transposase family protein	XP_001307942	4.00E-21	0.00E+00	413.5	(3,4)
TVAG_047800	small Rab GTPase Rab1b	XP_001311109	2.00E-80	1.00E+00	-183.5	(1,5)
TVAG_452120	GTP-binding protein alpha subunit 1	XP_001580284	8.00E-56	1.00E+00	-126.9	(1,6)

*Excluding hits on self: Parabasalia (the higher taxonomic rank containing *Trichomonas vaginalis),* identified with restricting the BlastP search against eukaryotes (NCBI nr database)

**Identified using default BlastP searches (NCBI nr database), invariably hits from Bacteria for listed exemples of LGT

A value of 1 is used in case there are no hits for a given BlastP search

***References**

(1) Carlton et al. (2007). Science, 315: 207-212.

(2) Alsmark et al. (2013). Genome Biology, 14: R19.

(3) Strese et al. (2014) BMC Evolutionary Biology, 14:100

(4) Hirt et al. (2015). Current Opinion in Microbiology, 23: 155-162.

(5) Lal et al. (2005). Molecular and Biochemical Parasitology, 143: 226–235.

(6) Hirt et al. (2003). Molecular and Biochemical Parasitology, 129: 179–189.
