# Supplementary material for: The Protozoan Trichomonas vaginalis Targets Bacteria with Laterally Acquired NlpC/P60 Peptidoglycan Hydrolases
Source: mBio. 2018 Dec 11;9(6):e01784-18. doi: 10.1128/mBio.01784-18 (PMC6299479; doi:10.1128/mBio.01784-18)
Supplement: TABLE S2 [file mbo006184213st2.pdf]

Tax BLAST report

Tax BLAST report

**RID**	P7SZB6FE014 (Expires on 07-12 04:30 am)
**Query ID**	lcl|Query_260864
**Description**	None
**Molecule type**	amino acid
**Query Length**	275
**Database Name**	nr
**Description**	All non-redundant GenBank CDS translations+PDB+SwissProt+ excluding environmental samples from WGS projects
**Program**	BLASTP 2.6.1+

Lineage Report

Organism Report Taxonomy Report

Organism	Blast Name	Score	Number of Hits	Description
cellular organisms			158	
.Trichomonas vaginalis G3	trichomonads	574	8	Trichomonas vaginalis G3 hits
.Tomitella biformata	high GC Gram+	111	1	Tomitella biformata hits
.Papillibacter cinnamivorans	firmicutes	102	1	Papillibacter cinnamivorans hits
.Papillibacter cinnamivorans DSM 12816	firmicutes	102	1	Papillibacter cinnamivorans DSM 12816 hits
.Streptomyces sp. TverLS-915	high GC Gram+	96.7	1	Streptomyces sp. TverLS-915 hits
.Pseudonocardia	high GC Gram+	100	2	Pseudonocardia hits
.Pseudonocardia spinosispora	high GC Gram+	98.2	1	Pseudonocardia spinosispora hits
.Streptomyces sp. LcepLS	high GC Gram+	95.9	1	Streptomyces sp. LcepLS hits
.[Clostridium] josui	firmicutes	99.0	1	[Clostridium] josui hits
.Massiliomicrobiota sp. An142	firmicutes	95.1	2	Massiliomicrobiota sp. An142 hits
.Rhodococcus phenolicus	high GC Gram+	97.1	1	Rhodococcus phenolicus hits
.Rhodococcus zopfii	high GC Gram+	96.7	1	Rhodococcus zopfii hits
.Rhodococcus opacus	high GC Gram+	95.9	3	Rhodococcus opacus hits
.Rhodococcus opacus B4	high GC Gram+	96.3	1	Rhodococcus opacus B4 hits
.Rhodococcus kyotonensis	high GC Gram+	96.3	2	Rhodococcus kyotonensis hits

.Lawsonella clevelandensis	high GC Gram+	99.4	1	**Lawsonella clevelandensis hits**
.Rhodococcus koreensis	high GC Gram+	95.9	2	**Rhodococcus koreensis hits**
.Pseudonocardia sp. Ae707_Ps1	high GC Gram+	100	1	**Pseudonocardia sp. Ae707_Ps1 hits**
.Rhodococcus sp. NCIMB 12038	high GC Gram+	95.5	2	**Rhodococcus sp. NCIMB 12038 hits**
.Rhodococcus rhodochrous	high GC Gram+	92.4	1	**Rhodococcus rhodochrous hits**
.Rhodococcus	high GC Gram+	94.7	8	**Rhodococcus hits**
.Rhodococcus sp. EsD8	high GC Gram+	94.7	1	**Rhodococcus sp. EsD8 hits**
.Rhodococcus rhodochrous ATCC 21198	high GC Gram+	94.7	1	**Rhodococcus rhodochrous ATCC 21198 hits**
.Rhodococcus aetherivorans	high GC Gram+	94.7	2	**Rhodococcus aetherivorans hits**
.Rhodococcus sp. WB1	high GC Gram+	94.7	1	**Rhodococcus sp. WB1 hits**
.Pseudonocardia sp. Ae150A_Ps1	high GC Gram+	98.6	1	**Pseudonocardia sp. Ae150A_Ps1 hits**
.Pseudonocardia sp. Ae168_Ps1	high GC Gram+	98.6	1	**Pseudonocardia sp. Ae168_Ps1 hits**
.Pseudonocardia sp. Ae263_Ps1	high GC Gram+	98.6	1	**Pseudonocardia sp. Ae263_Ps1 hits**
.Pseudonocardia sp. Ae356_Ps1	high GC Gram+	98.6	1	**Pseudonocardia sp. Ae356_Ps1 hits**
.Smaragdicoccus niigatensis	high GC Gram+	94.0	1	**Smaragdicoccus niigatensis hits**
.Rhodococcus jostii	high GC Gram+	94.0	2	**Rhodococcus jostii hits**
.Rhodococcus opacus PD630	high GC Gram+	94.0	2	**Rhodococcus opacus PD630 hits**
.Rhodococcus sp. SC4	high GC Gram+	94.0	1	**Rhodococcus sp. SC4 hits**
.Rhodococcus sp. LB1	high GC Gram+	94.0	1	**Rhodococcus sp. LB1 hits**
.Rhodococcus sp. M8	high GC Gram+	94.0	2	**Rhodococcus sp. M8 hits**
.Pseudonocardia sp. HH130630-07	high GC Gram+	94.4	2	**Pseudonocardia sp. HH130630-07 hits**
.Streptomyces sp. NRRL F-5630	high GC Gram+	97.4	1	**Streptomyces sp. NRRL F-5630 hits**
.Rhodococcus yunnanensis	high GC Gram+	93.6	1	**Rhodococcus yunnanensis hits**
.Rhodococcus sp. WMMA185	high GC Gram+	92.8	2	**Rhodococcus sp. WMMA185 hits**
.Rhodococcus wratislaviensis	high GC			**Rhodococcus wratislaviensis NBRC**

NBRC 100605	Gram+	93.6	1	**100605 hits**
.Rhodococcus imtechensis RKJ300 = JCM 13270	high GC Gram+	93.2	1	**Rhodococcus imtechensis RKJ300 = JCM 13270 hits**
.Rhodococcus opacus M213	high GC Gram+	93.2	1	**Rhodococcus opacus M213 hits**
.Rhodococcus wratislaviensis IFP 2016	high GC Gram+	93.2	1	**Rhodococcus wratislaviensis IFP 2016 hits**
.Rhodococcus triatomae	high GC Gram+	92.8	2	**Rhodococcus triatomae hits**
.Rhodococcus wratislaviensis	high GC Gram+	93.2	1	**Rhodococcus wratislaviensis hits**
.Rhodococcus sp. CUA-806	high GC Gram+	93.2	2	**Rhodococcus sp. CUA-806 hits**
.Rhodococcus sp. JVH1	high GC Gram+	93.2	2	**Rhodococcus sp. JVH1 hits**
.Flavonifractor sp. An4	firmicutes	95.9	2	**Flavonifractor sp. An4 hits**
.Rhodococcus sp. AW25M09	high GC Gram+	92.0	2	**Rhodococcus sp. AW25M09 hits**
.Rhodococcus sp. B7740	high GC Gram+	92.4	1	**Rhodococcus sp. B7740 hits**
.Clostridioides difficile	firmicutes	94.0	3	**Clostridioides difficile hits**
.Streptomyces sp. Ncost-T6T-2b	high GC Gram+	92.8	1	**Streptomyces sp. Ncost-T6T-2b hits**
.Streptomyces aidingensis	high GC Gram+	96.3	1	**Streptomyces aidingensis hits**
.Rhodococcus sp. 29MFTsu3.1	high GC Gram+	92.4	1	**Rhodococcus sp. 29MFTsu3.1 hits**
.Streptomyces fulvissimus	high GC Gram+	95.9	1	**Streptomyces fulvissimus hits**
.Streptomyces fulvissimus DSM 40593	high GC Gram+	95.9	1	**Streptomyces fulvissimus DSM 40593 hits**
.Coprobacillus	firmicutes	93.6	1	**Coprobacillus hits**
.Coprobacillus sp. 29_1	firmicutes	93.6	1	**Coprobacillus sp. 29_1 hits**
.Streptomyces luridiscabiei	high GC Gram+	95.9	1	**Streptomyces luridiscabiei hits**
.Millisia brevis	high GC Gram+	95.9	1	**Millisia brevis hits**
.Flavonifractor plautii	firmicutes	95.1	7	**Flavonifractor plautii hits**
.Rhodococcus coprophilus	high GC Gram+	92.4	1	**Rhodococcus coprophilus hits**
.Dietzia timorensis	high GC Gram+	92.8	2	**Dietzia timorensis hits**
.Streptomyces scopuliridis	high GC Gram+	95.5	1	**Streptomyces scopuliridis hits**
.Flavonifractor plautii ATCC 29863	firmicutes	94.7	1	**Flavonifractor plautii ATCC 29863 hits**
.Pseudonocardia acaciae	high GC Gram+	90.5	1	**Pseudonocardia acaciae hits**

.Streptomyces sp. NRRL F-5193	high GC Gram+	95.5	1	**Streptomyces sp. NRRL F-5193 hits**
.Clostridiales	firmicutes	94.7	1	**Clostridiales hits**
.Lachnospiraceae bacterium 7_1_58FAA	firmicutes	94.7	1	**Lachnospiraceae bacterium 7_1_58FAA hits**
.Clostridium sp. ATCC BAA-442	firmicutes	94.7	1	**Clostridium sp. ATCC BAA-442 hits**
.Clostridium orbiscindens 1_3_50AFAA	firmicutes	94.7	1	**Clostridium orbiscindens 1_3_50AFAA hits**
.Nocardia transvalensis	high GC Gram+	92.0	1	**Nocardia transvalensis hits**
.Streptomyces griseoplanus	high GC Gram+	95.1	1	**Streptomyces griseoplanus hits**
.Flavonifractor sp. An112	firmicutes	94.4	2	**Flavonifractor sp. An112 hits**
.Rhodococcus fascians	high GC Gram+	91.3	1	**Rhodococcus fascians hits**
.[Clostridium] dakarense	firmicutes	95.1	1	**[Clostridium] dakarense hits**
.Rhodococcus ruber	high GC Gram+	91.7	1	**Rhodococcus ruber hits**
.Rhodococcus ruber Chol-4	high GC Gram+	91.7	1	**Rhodococcus ruber Chol-4 hits**
.uncultured Flavonifractor sp.	firmicutes	94.0	1	**uncultured Flavonifractor sp. hits**
.Nocardia pseudobrasiliensis	high GC Gram+	94.4	1	**Nocardia pseudobrasiliensis hits**
.Rhodococcus sp. 164Chir2E	high GC Gram+	90.9	1	**Rhodococcus sp. 164Chir2E hits**
.Pseudonocardia autotrophica	high GC Gram+	92.8	1	**Pseudonocardia autotrophica hits**
.Rhodococcus tukisamuensis	high GC Gram+	94.4	2	**Rhodococcus tukisamuensis hits**
.Streptomyces glaucescens	high GC Gram+	94.4	1	**Streptomyces glaucescens hits**
.Pseudonocardia sp. HH130629-09	high GC Gram+	90.5	1	**Pseudonocardia sp. HH130629-09 hits**
.Streptomyces pharetrae	high GC Gram+	94.0	1	**Streptomyces pharetrae hits**
.Streptomyces pharetrae CZA14	high GC Gram+	94.0	1	**Streptomyces pharetrae CZA14 hits**
.Rhodococcus ruber BKS 20-38	high GC Gram+	90.9	1	**Rhodococcus ruber BKS 20-38 hits**
.Rhodococcus rhodochrous KG-21	high GC Gram+	90.9	1	**Rhodococcus rhodochrous KG-21 hits**
.Flavonifractor sp. An91	firmicutes	93.6	2	**Flavonifractor sp. An91 hits**
.Intestinimonas butyriciproducens	firmicutes	93.2	3	**Intestinimonas butyriciproducens hits**
.Rhodococcus erythropolis PR4	high GC Gram+	90.5	1	**Rhodococcus erythropolis PR4 hits**
.Rhodococcus erythropolis CCM2595	high GC Gram+	90.5	1	**Rhodococcus erythropolis CCM2595 hits**

.Rhodococcus erythropolis DN1	high GC Gram+	90.5	1	Rhodococcus erythropolis DN1 hits
.Rhodococcus sp. P27	high GC Gram+	90.5	1	Rhodococcus sp. P27 hits
.Rhodococcus erythropolis	high GC Gram+	90.5	3	Rhodococcus erythropolis hits
.Rhodococcus erythropolis R138	high GC Gram+	90.5	1	Rhodococcus erythropolis R138 hits
.Rhodococcus sp. 66b	high GC Gram+	90.5	1	Rhodococcus sp. 66b hits
.Rhodococcus sp. 1159	high GC Gram+	90.5	1	Rhodococcus sp. 1159 hits
.Flavonifractor sp. An52	firmicutes	93.2	2	Flavonifractor sp. An52 hits
.Hoyosella altamirensis	high GC Gram+	94.0	1	Hoyosella altamirensis hits
.Hoyosella subflava DQS3-9A1	high GC Gram+	94.0	1	Hoyosella subflava DQS3-9A1 hits
.Streptomyces sp. NRRL WC-3618	high GC Gram+	93.6	1	Streptomyces sp. NRRL WC-3618 hits
.Hoyosella subflava	high GC Gram+	93.6	1	Hoyosella subflava hits
.uncultured Clostridium sp.	firmicutes	94.7	2	uncultured Clostridium sp. hits
.Flavonifractor	firmicutes	92.8	1	Flavonifractor hits
.Flavonifractor sp. An92	firmicutes	92.8	1	Flavonifractor sp. An92 hits
.Flavonifractor sp. An135	firmicutes	92.8	1	Flavonifractor sp. An135 hits

Organism Report

Lineage Report Taxonomy Report

Description	Score	E value	Accession
Trichomonas vaginalis G3 [trichomonads] Next Previous First			
Clan CA, family C40, NlpC/P60 superfamily cysteine peptidase [Trichomonas vaginalis G3]	574	0.0	XP_001276902
Clan CA, family C40, NlpC/P60 superfamily cysteine peptidase [Trichomonas vaginalis G3]	574	0.0	EAY23654
Clan CA, family C40, NlpC/P60 superfamily cysteine peptidase [Trichomonas vaginalis G3]	526	0.0	XP_001583075
Clan CA, family C40, NlpC/P60 superfamily cysteine peptidase [Trichomonas vaginalis G3]	526	0.0	EAY22089
Clan CA, family C40, NlpC/P60 superfamily cysteine peptidase [Trichomonas vaginalis G3]	267	5e-86	XP_001330233

Clan CA, family C40, NlpC/P60 superfamily cysteine peptidase [Trichomonas vaginalis G3]	267	5e-86	EAY01385
Clan CA, family C40, NlpC/P60 superfamily cysteine peptidase [Trichomonas vaginalis G3]	155	9e-44	XP_001314869
Clan CA, family C40, NlpC/P60 superfamily cysteine peptidase [Trichomonas vaginalis G3]	155	9e-44	EAY02646
Tomitella biformata [high GC Gram+] Next Previous First			
hypothetical protein [Tomitella biformata]	111	3e-25	WP_024793165
Papillibacter cinnamivorans [firmicutes] Next Previous First			
hypothetical protein [Papillibacter cinnamivorans]	102	5e-22	WP_084233577
Papillibacter cinnamivorans DSM 12816 [firmicutes] Next Previous First			
SH3 domain-containing protein [Papillibacter cinnamivorans DSM 12816]	102	5e-22	SMC45656
Streptomyces sp. TverLS-915 [high GC Gram+] Next Previous First			
NlpC/P60 family protein, partial [Streptomyces sp. TverLS-915]	96.7	7e-22	SCD97746
Pseudonocardia [high GC Gram+] Next Previous First			
MULTISPECIES: hypothetical protein [Pseudonocardia]	100	2e-21	WP_082399349
MULTISPECIES: hypothetical protein [Pseudonocardia]	98.6	6e-21	WP_083680407
Pseudonocardia spinosispora [high GC Gram+] Next Previous First			
hypothetical protein [Pseudonocardia spinosispora]	98.2	2e-21	WP_084216722
Streptomyces sp. LcepLS			

[
high GC Gram+
]
Next
Previous
First

NlpC/P60 family protein, partial [Streptomyces sp. LcepLS]	95.9	3e-21	SCE67218

Clostridium josui
[
firmicutes
]
Next
Previous
First

glycoside hydrolase [[Clostridium] josui]	99.0	4e-21	WP_024831455

Massiliomicrobiota sp. An142
[
firmicutes
]
Next
Previous
First

hypothetical protein [Massiliomicrobiota sp. An142]	95.1	4e-21	WP_087244740
hypothetical protein B5E87_09510 [Massiliomicrobiota sp. An142]	95.1	4e-21	OUQ12552

Rhodococcus phenolicus
[
high GC Gram+
]
Next
Previous
First

hydrolase [Rhodococcus phenolicus]	97.1	6e-21	WP_068165089

Rhodococcus zopfii
[
high GC Gram+
]
Next
Previous
First

hydrolase [Rhodococcus zopfii]	96.7	8e-21	WP_072813788

Rhodococcus opacus
[
high GC Gram+
]
Next
Previous
First

hydrolase [Rhodococcus opacus]	95.9	9e-21	WP_080512436
hydrolase [Rhodococcus opacus]	93.6	1e-19	AII10010
NlpC/P60 family protein [Rhodococcus opacus]	93.2	1e-19	ANS29340

Rhodococcus opacus B4
[
high GC Gram+
]
Next
Previous
First

NlpC/P60 family protein [Rhodococcus opacus B4]	96.3	9e-21	BAH49100

Rhodococcus kyotonensis
[
high GC Gram+
]
Next
Previous
First

hydrolase [Rhodococcus kyotonensis]	96.3	1e-20	WP_084423498
hydrolase [Rhodococcus kyotonensis]	96.3	1e-20	OAK57553

Corynebacteriales bacterium X1036
[
high GC Gram+
]
Next
Previous
First

hypothetical protein [Lawsonella clevelandensis]	99.4	1e-20	WP_053962500

Rhodococcus koreensis
[
high GC Gram+
]
Next
Previous
First

Cell wall-associated hydrolase, NlpC family [Rhodococcus koreensis]	95.9	1e-20	SED45794
hydrolase [Rhodococcus koreensis]	95.5	1e-20	WP_083395829

Pseudonocardia sp. Ae707_Ps1
[
high GC Gram+
]
Next
Previous
First

putative secreted protein [Pseudonocardia sp. Ae707_Ps1]	100	1e-20	OLM19556

Rhodococcus sp. NCIMB 12038
[
high GC Gram+
]
Next
Previous
First

hydrolase [Rhodococcus sp. NCIMB 12038]	95.5	1e-20	WP_087556217
hydrolase [Rhodococcus sp. NCIMB 12038]	95.5	1e-20	OUS96538

Rhodococcus rhodochrous
[
high GC Gram+
]
Next
Previous
First

hydrolase, partial [Rhodococcus rhodochrous]	92.4	4e-20	OOL27418

Rhodococcus
[
high GC Gram+
]
Next
Previous
First

MULTISPECIES: hypothetical protein [Rhodococcus]	94.7	4e-20	WP_006932087
MULTISPECIES: hydrolase [Rhodococcus]	93.6	8e-20	WP_080578264
MULTISPECIES: hydrolase [Rhodococcus]	93.2	1e-19	WP_005260127
MULTISPECIES: hydrolase [Rhodococcus]	92.4	2e-19	WP_082073676
MULTISPECIES: hypothetical protein [Rhodococcus]	92.4	2e-19	WP_020111964
MULTISPECIES: hydrolase [Rhodococcus]	91.7	7e-19	WP_010594353
MULTISPECIES: peptidase [Rhodococcus]	90.9	1e-18	WP_003934126
MULTISPECIES: hydrolase [Rhodococcus]	90.5	1e-18	WP_019748904

Rhodococcus sp. EsD8
[
high GC Gram+
]
Next
Previous
First

possible secreted protein [Rhodococcus sp. EsD8]	94.7	4e-20	CCW10127

Rhodococcus rhodochrous ATCC 21198
[
high GC Gram+
]
Next
Previous
First

NLP/P60 protein [Rhodococcus rhodochrous ATCC 21198]	94.7	4e-20	ETT25912

Rhodococcus aetherivorans
[
high GC Gram+
]
Next
Previous
First

hydrolase [Rhodococcus aetherivorans]	94.7	4e-20	KDE12839
hydrolase [Rhodococcus aetherivorans]	94.7	4e-20	AKE90416

Rhodococcus sp. WB1
[
high GC Gram+
]
Next
Previous
First

hydrolase [Rhodococcus sp. WB1]	94.7	4e-20	ANZ24854

Pseudonocardia sp. Ae150A_Ps1
[
high GC Gram+
]
Next
Previous
First

putative secreted protein [Pseudonocardia sp. Ae150A_Ps1]	98.6	5e-20	OLL73013

Pseudonocardia sp. Ae168_Ps1
[
high GC Gram+
]
Next
Previous
First

putative secreted protein [Pseudonocardia sp. Ae168_Ps1]	98.6	5e-20	OLL78989
Pseudonocardia sp. Ae263_Ps1 [high GC Gram+] Next Previous First			
putative secreted protein [Pseudonocardia sp. Ae263_Ps1]	98.6	5e-20	OLL86873
Pseudonocardia sp. Ae356_Ps1 [high GC Gram+] Next Previous First			
putative secreted protein [Pseudonocardia sp. Ae356_Ps1]	98.6	5e-20	OLL93082
Smaragdicoccus niigatensis [high GC Gram+] Next Previous First			
hypothetical protein [Smaragdicoccus niigatensis]	94.0	6e-20	WP_018161020
Rhodococcus jostii [high GC Gram+] Next Previous First			
Cell wall-associated hydrolase, NlpC family [Rhodococcus jostii]	94.0	7e-20	SEC74907
hydrolase [Rhodococcus jostii]	93.6	8e-20	WP_083400453
Rhodococcus opacus PD630 [high GC Gram+] Next Previous First			
secreted protein [Rhodococcus opacus PD630]	94.0	8e-20	EHI42419
Uncharacterized protein Pd630_LPD05221 [Rhodococcus opacus PD630]	94.0	8e-20	AHK32427
Rhodococcus sp. SC4 [high GC Gram+] Next Previous First			
hydrolase [Rhodococcus sp. SC4]	94.0	8e-20	KXF51439
Rhodococcus sp. LB1 [high GC Gram+] Next			

Previous
First

hydrolase [Rhodococcus sp. LB1]	94.0	8e-20	KXX60370

Rhodococcus sp. M8
[
high GC Gram+
]
Next
Previous
First

hydrolase [Rhodococcus sp. M8]	94.0	8e-20	WP_072635440
hydrolase [Rhodococcus sp. M8]	94.0	8e-20	OLL16379

Pseudonocardia sp. HH130630-07
[
high GC Gram+
]
Next
Previous
First

hypothetical protein [Pseudonocardia sp. HH130630-07]	94.4	8e-20	WP_083276114
hypothetical protein AFB00_22380 [Pseudonocardia sp. HH130630-07]	95.1	7e-19	ANY08560

Streptomyces sp. NRRL F-5630
[
high GC Gram+
]
Next
Previous
First

hypothetical protein [Streptomyces sp. NRRL F-5630]	97.4	9e-20	WP_030994515

Rhodococcus yunnanensis
[
high GC Gram+
]
Next
Previous
First

hydrolase [Rhodococcus yunnanensis]	93.6	1e-19	WP_084727750

Rhodococcus sp. SCSIO 00026
[
high GC Gram+
]
Next
Previous
First

hydrolase [Rhodococcus sp. WMMA185]	92.8	1e-19	AOW94731
hydrolase [Rhodococcus sp. WMMA185]	93.2	1e-19	WP_070380786

Rhodococcus wratislaviensis NBRC 100605
[
high GC Gram+
]
Next
Previous
First

NlpC/P60 family protein [Rhodococcus wratislaviensis NBRC 100605]	93.6	1e-19	GAF42584

Rhodococcus imtechensis RKJ300 = JCM 13270
[

high GC Gram+
]
Next
Previous
First

NlpC/P60 family protein [Rhodococcus imtechensis RKJ300 = JCM 13270]	93.2	1e-19	EID79696

Rhodococcus opacus M213
[
high GC Gram+
]
Next
Previous
First

NlpC/P60 family protein [Rhodococcus opacus M213]	93.2	1e-19	EKT80169

Rhodococcus wratislaviensis IFP 2016
[
high GC Gram+
]
Next
Previous
First

NlpC/P60 family protein [Rhodococcus wratislaviensis IFP 2016]	93.2	1e-19	ELB87078

Rhodococcus triatomae
[
high GC Gram+
]
Next
Previous
First

hydrolase [Rhodococcus triatomae]	92.8	1e-19	WP_083342904
Cell wall-associated hydrolase, NlpC family [Rhodococcus triatomae]	93.2	1e-19	SDH53046

Rhodococcus wratislaviensis
[
high GC Gram+
]
Next
Previous
First

hydrolase [Rhodococcus wratislaviensis]	93.2	1e-19	WP_081792183

Rhodococcus sp. CUA-806
[
high GC Gram+
]
Next
Previous
First

hydrolase [Rhodococcus sp. CUA-806]	93.2	1e-19	WP_083668028
hydrolase [Rhodococcus sp. CUA-806]	93.2	1e-19	OLT32268

Rhodococcus sp. JVH1
[
high GC Gram+
]
Next
Previous
First

hydrolase [Rhodococcus sp. JVH1]	93.2	1e-19	WP_043778150

nlpC/P60 family protein [Rhodococcus sp. JVH1]	92.8	1e-19	EJJ00936

Flavonifractor sp. An4
[
firmicutes
]
Next
Previous
First

hypothetical protein [Flavonifractor sp. An4]	95.9	2e-19	WP_087328846
hypothetical protein B5F94_06455 [Flavonifractor sp. An4]	95.9	2e-19	OUO15867

Rhodococcus sp. AW25M09
[
high GC Gram+
]
Next
Previous
First

Gamma-DL-glutamyl hydrolase [Rhodococcus sp. AW25M09]	92.0	2e-19	CCQ16141
hydrolase [Rhodococcus sp. AW25M09]	91.7	4e-19	WP_081606004

Rhodococcus sp. B7740
[
high GC Gram+
]
Next
Previous
First

secreted protein [Rhodococcus sp. B7740]	92.4	2e-19	AJW38695

[Clostridium] difficile
[
firmicutes
]
Next
Previous
First

Murein DD-endopeptidase MepH precursor [Clostridioides difficile]	94.0	2e-19	CZS06512
phage cell wall hydrolase [Clostridioides difficile]	95.1	1e-18	SJW17689
cell wall hydrolase [Clostridioides difficile]	95.1	2e-18	WP_074074660

Streptomyces sp. Ncost-T6T-2b
[
high GC Gram+
]
Next
Previous
First

NlpC/P60 family protein, partial [Streptomyces sp. Ncost-T6T-2b]	92.8	2e-19	SCF62354

Streptomyces sp. TRM46012
[
high GC Gram+
]
Next
Previous
First

NlpC/P60 family protein [Streptomyces aidingensis]	96.3	2e-19	SFC72198

Rhodococcus sp. 29MFTsu3.1
[
high GC Gram+

] Next Previous First			
hypothetical protein [Rhodococcus sp. 29MFTsu3.1]<br><br>Streptomyces fulvissimus [high GC Gram+] Next Previous First	92.4	2e-19	WP_019665961
NPL/P60-family secreted protein [Streptomyces fulvissimus]<br><br>Streptomyces fulvissimus DSM 40593 [high GC Gram+] Next Previous First	95.9	3e-19	WP_015608048
NPL/P60-family secreted protein [Streptomyces fulvissimus DSM 40593]<br><br>Coprobacillus [firmicutes] Next Previous First	95.9	3e-19	AGK76677
MULTISPECIES: NLP/P60 protein [Coprobacillus]<br><br>Coprobacillus sp. 29_1 [firmicutes] Next Previous First	93.6	3e-19	WP_008790283
NLP/P60 protein [Coprobacillus sp. 29_1]<br><br>Streptomyces luridiscabiei [high GC Gram+] Next Previous First	93.6	3e-19	EFW03510
hypothetical protein [Streptomyces luridiscabiei]<br><br>Millisia brevis [high GC Gram+] Next Previous First	95.9	3e-19	WP_055558940
protein p60 precursor [Millisia brevis]<br><br>Flavonifractor plautii [firmicutes] Next Previous	95.9	3e-19	WP_066907071

First

hypothetical protein [Flavonifractor plautii]	95.1	3e-19	WP_065535124
hypothetical protein A4U99_12990 [Flavonifractor plautii]	95.1	3e-19	ANU41923
hypothetical protein [Flavonifractor plautii]	94.7	4e-19	WP_007495864
Probable endopeptidase p60 precursor [Flavonifractor plautii]	94.7	4e-19	CUP73593
Probable endopeptidase p60 precursor [Flavonifractor plautii]	94.7	4e-19	CUP19434
peptidase PgdS [Flavonifractor plautii]	94.7	4e-19	CUP11954
hypothetical protein B5F52_10970 [Flavonifractor plautii]	94.7	4e-19	OUO82082

Rhodococcus coprophilus
[
high GC Gram+
]
Next
Previous
First

hydrolase [Rhodococcus coprophilus]	92.4	3e-19	WP_072699190

Dietzia timorensis
[
high GC Gram+
]
Next
Previous
First

hydrolase [Dietzia timorensis]	92.8	3e-19	WP_067473211
putative endopeptidase [Dietzia timorensis]	92.8	3e-19	ANI92137

Streptomyces scopuliridis
[
high GC Gram+
]
Next
Previous
First

hypothetical protein [Streptomyces scopuliridis]	95.5	4e-19	WP_030353966

Flavonifractor plautii ATCC 29863
[
firmicutes
]
Next
Previous
First

NlpC/P60 family protein [Flavonifractor plautii ATCC 29863]	94.7	4e-19	EHM37251

Pseudonocardia acaciae
[
high GC Gram+
]
Next
Previous
First

hypothetical protein [Pseudonocardia acaciae]	90.5	4e-19	WP_084211398

Streptomyces sp. NRRL F-5193
[
high GC Gram+
]
Next
Previous

First			
hypothetical protein [Streptomyces sp. NRRL F-5193]<br><br>Clostridiales<br>[<br>firmicutes<br>]<br>Next<br>Previous<br>First	95.5	4e-19	WP_043228583
MULTISPECIES: hypothetical protein [Clostridiales]<br><br>Lachnospiraceae bacterium 7_1_58FAA<br>[<br>firmicutes<br>]<br>Next<br>Previous<br>First	94.7	4e-19	WP_009259268
hypothetical protein HMPREF0995_03110 [Lachnospiraceae bacterium 7_1_58FAA]<br><br>Clostridium sp. ATCC BAA-442<br>[<br>firmicutes<br>]<br>Next<br>Previous<br>First	94.7	4e-19	EHO32821
NlpC/P60 family protein [Clostridium sp. ATCC BAA-442]<br><br>Clostridium orbiscindens 1_3_50AFAA<br>[<br>firmicutes<br>]<br>Next<br>Previous<br>First	94.7	4e-19	ERI64028
hypothetical protein HMPREF9460_02718 [Clostridium orbiscindens 1_3_50AFAA]<br><br>Nocardia transvalensis<br>[<br>high GC Gram+<br>]<br>Next<br>Previous<br>First	94.7	4e-19	KGF54559
hydrolase [Nocardia transvalensis]<br><br>Streptomyces griseoplanus<br>[<br>high GC Gram+<br>]<br>Next<br>Previous<br>First	92.0	5e-19	WP_051161256
hypothetical protein [Streptomyces griseoplanus]<br><br>Flavonifractor sp. An112<br>[<br>firmicutes<br>]<br>Next<br>Previous<br>First	95.1	5e-19	WP_055586037

hypothetical protein [Flavonifractor sp. An112]	94.4	6e-19	WP_087388289
hypothetical protein B5E56_02340 [Flavonifractor sp. An112]<br>Rhodococcus fascians<br>[<br>high GC Gram+<br>]<br>Next<br>Previous<br>First	94.4	6e-19	OUQ61558
hydrolase [Rhodococcus fascians]<br>Clostridium sp. 01<br>[<br>firmicutes<br>]<br>Next<br>Previous<br>First	91.3	6e-19	WP_082323735
cell wall hydrolase [[Clostridium] dakarense]<br>Rhodococcus ruber<br>[<br>high GC Gram+<br>]<br>Next<br>Previous<br>First	95.1	6e-19	WP_042275565
putative enzyme [Rhodococcus ruber]<br>Rhodococcus ruber Chol-4<br>[<br>high GC Gram+<br>]<br>Next<br>Previous<br>First	91.7	7e-19	CDZ92109
hydrolase [Rhodococcus ruber Chol-4]<br>uncultured Flavonifractor sp.<br>[<br>firmicutes<br>]<br>Next<br>Previous<br>First	91.7	7e-19	KXF87178
Probable endopeptidase p60 precursor [uncultured Flavonifractor sp.]<br>Nocardia pseudobrasiliensis<br>[<br>high GC Gram+<br>]<br>Next<br>Previous<br>First	94.0	7e-19	SCI88609
protein p60 precursor [Nocardia pseudobrasiliensis]<br>Rhodococcus sp. 164Chir2E<br>[<br>high GC Gram+<br>]<br>Next<br>Previous<br>First	94.4	9e-19	WP_068006432
Cell wall-associated hydrolase, NlpC family [Rhodococcus sp. 164Chir2E]	90.9	9e-19	SCY12554

Pseudonocardia autotrophica
[
high GC Gram+
]
Next
Previous
First

hypothetical protein [Pseudonocardia autotrophica]	92.8	1e-18	WP_085914689

Rhodococcus tukisamuensis
[
high GC Gram+
]
Next
Previous
First

protein p60 precursor [Rhodococcus tukisamuensis]	94.4	1e-18	WP_072847402
Cell wall-associated hydrolase, NlpC family [Rhodococcus tukisamuensis]	94.4	1e-18	SDE27564

Streptomyces glaucescens
[
high GC Gram+
]
Next
Previous
First

hypothetical protein [Streptomyces glaucescens]	94.4	1e-18	WP_086732478

Pseudonocardia sp. HH130629-09
[
high GC Gram+
]
Next
Previous
First

hypothetical protein [Pseudonocardia sp. HH130629-09]	90.5	1e-18	WP_082375780

Streptomyces pharetrae
[
high GC Gram+
]
Next
Previous
First

hypothetical protein [Streptomyces pharetrae]	94.0	1e-18	WP_086169026

Streptomyces pharetrae CZA14
[
high GC Gram+
]
Next
Previous
First

hypothetical protein OQI_10225 [Streptomyces pharetrae CZA14]	94.0	1e-18	OSZ60535

Rhodococcus ruber BKS 20-38
[
high GC Gram+
]
Next
Previous
First

peptidase [Rhodococcus ruber BKS 20-38]	90.9	1e-18	EME67404

Rhodococcus rhodochrous KG-21
[
high GC Gram+
]
Next
Previous
First

hydrolase [Rhodococcus rhodochrous KG-21]	90.9	1e-18	KOS53142

Flavonifractor sp. An91
[
firmicutes
]
Next
Previous
First

hypothetical protein [Flavonifractor sp. An91]	93.6	1e-18	WP_087266797
hypothetical protein B5G42_03895 [Flavonifractor sp. An91]	93.6	1e-18	OUN13807

Intestinimonas butyriciproducens
[
firmicutes
]
Next
Previous
First

hypothetical protein [Intestinimonas butyriciproducens]	93.2	1e-18	WP_058118067
NLP/P60 family protein [Intestinimonas butyriciproducens]	93.2	1e-18	ALP94646
hypothetical protein [Intestinimonas butyriciproducens]	93.2	1e-18	WP_033118156

Rhodococcus erythropolis PR4
[
high GC Gram+
]
Next
Previous
First

conserved hypothetical protein [Rhodococcus erythropolis PR4]	90.5	1e-18	BAH34298

Rhodococcus erythropolis CCM2595
[
high GC Gram+
]
Next
Previous
First

hypothetical protein O5Y_16450 [Rhodococcus erythropolis CCM2595]	90.5	1e-18	AGT93138

Rhodococcus erythropolis DN1
[
high GC Gram+
]
Next
Previous
First

hydrolase [Rhodococcus erythropolis DN1]	90.5	1e-18	EQM30760

Rhodococcus sp. P27
[
high GC Gram+
]
Next
Previous
First

hydrolase [Rhodococcus sp. P27]	90.5	1e-18	ERB52190

Rhodococcus erythropolis
[
high GC Gram+
]
Next
Previous
First

hydrolase [Rhodococcus erythropolis]	90.5	1e-18	AKD98170
putative endopeptidase precursor [Rhodococcus erythropolis]	90.5	1e-18	OFV74113
hydrolase [Rhodococcus erythropolis]	90.5	1e-18	OHF26329

Rhodococcus erythropolis R138
[
high GC Gram+
]
Next
Previous
First

hydrolase [Rhodococcus erythropolis R138]	90.5	1e-18	ALU70554

Rhodococcus sp. 66b
[
high GC Gram+
]
Next
Previous
First

putative endopeptidase [Rhodococcus sp. 66b]	90.5	1e-18	OQM80713

Rhodococcus sp. 1159
[
high GC Gram+
]
Next
Previous
First

hydrolase [Rhodococcus sp. 1159]	90.5	1e-18	ORI17378

Flavonifractor sp. An52
[
firmicutes
]
Next
Previous
First

hypothetical protein [Flavonifractor sp. An52]	93.2	1e-18	WP_087187734
hypothetical protein B5G06_03625 [Flavonifractor sp. An52]	93.2	1e-18	OUN85101

Hoyosella altamirensis
[
high GC Gram+
]
Next
Previous
First

hypothetical protein [Hoyosella altamirensis]	94.0	1e-18	WP_083962166

Amycolicicoccus subflavus DQS3-9A1
[
high GC Gram+
]
Next

Previous<br>First			
NlpC/P60 family protein [Hoyosella subflava DQS3-9A1]	94.0	1e-18	AEF39741
Streptomyces sp. NRRL WC-3618<br>[<br>high GC Gram+<br>]<br>Next<br>Previous<br>First			
hypothetical protein [Streptomyces sp. NRRL WC-3618]	93.6	2e-18	WP_053743879
Amycolicicoccus subflavus<br>[<br>high GC Gram+<br>]<br>Next<br>Previous<br>First			
hypothetical protein [Hoyosella subflava]	93.6	2e-18	WP_041450910
uncultured Clostridium sp.<br>[<br>firmicutes<br>]<br>Next<br>Previous<br>First			
Probable endopeptidase Spr precursor [uncultured Clostridium sp.]	94.7	2e-18	SCJ22669
Probable endopeptidase Spr precursor [uncultured Clostridium sp.]	94.7	2e-18	SCJ22520
Flavonifractor<br>[<br>firmicutes<br>]<br>Next<br>Previous<br>First			
MULTISPECIES: hypothetical protein [Flavonifractor]	92.8	2e-18	WP_087261361
Flavonifractor sp. An92<br>[<br>firmicutes<br>]<br>Next<br>Previous<br>First			
hypothetical protein B5G43_11190 [Flavonifractor sp. An92]	92.8	2e-18	OUN05825
Flavonifractor sp. An135<br>[<br>firmicutes<br>]<br>Next<br>Previous<br>First			
hypothetical protein B5E80_12650 [Flavonifractor sp. An135]	92.8	2e-18	OUQ22742

Taxonomy Report

Organism Report Lineage Report

Taxonomy	Number of hits	Number of Organisms	Description
cellular organisms	158	108	
. Trichomonas vaginalis G3	8	1	Trichomonas vaginalis G3 hits
. Terrabacteria group	150	107	
.. Actinobacteria	110	83	
... Corynebacteriales	82	57	
.... unclassified Corynebacteriales	2	2	
..... Tomitella biformata	1	1	Tomitella biformata hits
..... Lawsonella clevelandensis	1	1	Lawsonella clevelandensis hits
.... Nocardiaceae	74	50	
..... Rhodococcus	8	47	Rhodococcus hits
...... Rhodococcus phenolicus	1	1	Rhodococcus phenolicus hits
...... Rhodococcus zopfii	1	1	Rhodococcus zopfii hits
...... Rhodococcus opacus	3	4	Rhodococcus opacus hits
.... Rhodococcus opacus B4	1	1	Rhodococcus opacus B4 hits
.... Rhodococcus opacus PD630	2	1	Rhodococcus opacus PD630 hits
.... Rhodococcus opacus M213	1	1	Rhodococcus opacus M213 hits
...... Rhodococcus kyotonensis	2	1	Rhodococcus kyotonensis hits
...... Rhodococcus koreensis	2	1	Rhodococcus koreensis hits
...... Rhodococcus sp. NCIMB 12038	2	1	Rhodococcus sp. NCIMB 12038 hits
...... Rhodococcus rhodochrous	1	3	Rhodococcus rhodochrous hits
.... Rhodococcus rhodochrous ATCC 21198	1	1	Rhodococcus rhodochrous ATCC 21198 hits
.... Rhodococcus rhodochrous KG-21	1	1	Rhodococcus rhodochrous KG-21 hits
...... Rhodococcus sp. EsD8	1	1	Rhodococcus sp. EsD8 hits
...... Rhodococcus aetherivorans	2	1	Rhodococcus aetherivorans hits
...... Rhodococcus sp. WB1	1	1	Rhodococcus sp. WB1 hits
...... Rhodococcus jostii	2	1	Rhodococcus jostii hits
...... Rhodococcus sp. SC4	1	1	Rhodococcus sp. SC4 hits
...... Rhodococcus sp. LB1	1	1	Rhodococcus sp. LB1 hits
...... Rhodococcus sp. M8	2	1	Rhodococcus sp. M8 hits
...... Rhodococcus yunnanensis	1	1	Rhodococcus yunnanensis hits
...... Rhodococcus sp. WMMA185	2	1	Rhodococcus sp. WMMA185 hits
...... Rhodococcus wratislaviensis	1	3	Rhodococcus wratislaviensis hits
.... Rhodococcus wratislaviensis NBRC 100605	1	1	Rhodococcus wratislaviensis NBRC 100605 hits
.... Rhodococcus wratislaviensis IFP 2016	1	1	Rhodococcus wratislaviensis IFP 2016 hits
...... Rhodococcus imtechensis RKJ300 = JCM 13270	1	1	Rhodococcus imtechensis RKJ300 = JCM 13270 hits
...... Rhodococcus triatomae	2	1	Rhodococcus triatomae hits

...... Rhodococcus sp. CUA-806	2	1	Rhodococcus sp. CUA-806 hits
...... Rhodococcus sp. JVH1	2	1	Rhodococcus sp. JVH1 hits
...... Rhodococcus sp. AW25M09	2	1	Rhodococcus sp. AW25M09 hits
...... Rhodococcus sp. B7740	1	1	Rhodococcus sp. B7740 hits
...... Rhodococcus sp. 29MFTsu3.1	1	1	Rhodococcus sp. 29MFTsu3.1 hits
...... Rhodococcus coprophilus	1	1	Rhodococcus coprophilus hits
...... Rhodococcus fascians	1	1	Rhodococcus fascians hits
...... Rhodococcus ruber	1	3	Rhodococcus ruber hits
.... Rhodococcus ruber Chol-4	1	1	Rhodococcus ruber Chol-4 hits
.... Rhodococcus ruber BKS 20-38	1	1	Rhodococcus ruber BKS 20-38 hits
...... Rhodococcus sp. 164Chir2E	1	1	Rhodococcus sp. 164Chir2E hits
...... Rhodococcus tukisamuensis	2	1	Rhodococcus tukisamuensis hits
...... Rhodococcus erythropolis	3	5	Rhodococcus erythropolis hits
.... Rhodococcus erythropolis PR4	1	1	Rhodococcus erythropolis PR4 hits
.... Rhodococcus erythropolis CCM2595	1	1	Rhodococcus erythropolis CCM2595 hits
.... Rhodococcus erythropolis DN1	1	1	Rhodococcus erythropolis DN1 hits
.... Rhodococcus erythropolis R138	1	1	Rhodococcus erythropolis R138 hits
...... Rhodococcus sp. P27	1	1	Rhodococcus sp. P27 hits
...... Rhodococcus sp. 66b	1	1	Rhodococcus sp. 66b hits
...... Rhodococcus sp. 1159	1	1	Rhodococcus sp. 1159 hits
..... Smaragdicoccus niigatensis	1	1	Smaragdicoccus niigatensis hits
..... Nocardia	2	2	
...... Nocardia transvalensis	1	1	Nocardia transvalensis hits
...... Nocardia pseudobrasiliensis	1	1	Nocardia pseudobrasiliensis hits
.... Millisia brevis	1	1	Millisia brevis hits
.... Dietzia timorensis	2	1	Dietzia timorensis hits
.... Hoyosella	3	3	
..... Hoyosella altamirensis	1	1	Hoyosella altamirensis hits
..... Hoyosella subflava	1	2	Hoyosella subflava hits
...... Hoyosella subflava DQS3-9A1	1	1	Hoyosella subflava DQS3-9A1 hits
... Streptomyces	15	15	
.... Streptomyces sp. TverLS-915	1	1	Streptomyces sp. TverLS-915 hits
.... Streptomyces sp. LcepLS	1	1	Streptomyces sp. LcepLS hits
.... Streptomyces sp. NRRL F-5630	1	1	Streptomyces sp. NRRL F-5630 hits
.... Streptomyces sp. Ncost-T6T-2b	1	1	Streptomyces sp. Ncost-T6T-2b hits
.... Streptomyces aidingensis	1	1	Streptomyces aidingensis hits
.... Streptomyces fulvissimus	1	2	Streptomyces fulvissimus hits
..... Streptomyces fulvissimus DSM 40593	1	1	Streptomyces fulvissimus DSM 40593 hits
.... Streptomyces luridiscabiei	1	1	Streptomyces luridiscabiei hits
.... Streptomyces scopuliridis	1	1	Streptomyces scopuliridis hits

.... Streptomyces sp. NRRL F-5193	1	1	Streptomyces sp. NRRL F-5193 hits
.... Streptomyces griseoplanus	1	1	Streptomyces griseoplanus hits
.... Streptomyces glaucescens	1	1	Streptomyces glaucescens hits
.... Streptomyces pharetrae	1	2	Streptomyces pharetrae hits
..... Streptomyces pharetrae CZA14	1	1	Streptomyces pharetrae CZA14 hits
.... Streptomyces sp. NRRL WC-3618	1	1	Streptomyces sp. NRRL WC-3618 hits
... Pseudonocardiaceae	13	11	
.... Pseudonocardia	2	11	Pseudonocardia hits
..... Pseudonocardia spinosispora	1	1	Pseudonocardia spinosispora hits
..... Pseudonocardia sp. Ae707_Ps1	1	1	Pseudonocardia sp. Ae707_Ps1 hits
..... Pseudonocardia sp. Ae150A_Ps1	1	1	Pseudonocardia sp. Ae150A_Ps1 hits
..... Pseudonocardia sp. Ae168_Ps1	1	1	Pseudonocardia sp. Ae168_Ps1 hits
..... Pseudonocardia sp. Ae263_Ps1	1	1	Pseudonocardia sp. Ae263_Ps1 hits
..... Pseudonocardia sp. Ae356_Ps1	1	1	Pseudonocardia sp. Ae356_Ps1 hits
..... Pseudonocardia sp. HH130630-07	2	1	Pseudonocardia sp. HH130630-07 hits
..... Pseudonocardia acaciae	1	1	Pseudonocardia acaciae hits
..... Pseudonocardia autotrophica	1	1	Pseudonocardia autotrophica hits
..... Pseudonocardia sp. HH130629-09	1	1	Pseudonocardia sp. HH130629-09 hits
.. Firmicutes	40	24	
... Clostridia	36	21	
.... Clostridiales	1	21	Clostridiales hits
..... Ruminococcaceae	3	3	
...... Papillibacter	2	2	
.... Papillibacter cinnamivorans	1	2	Papillibacter cinnamivorans hits
.... Papillibacter cinnamivorans DSM 12816	1	1	Papillibacter cinnamivorans DSM 12816 hits
...... [Clostridium] josui	1	1	[Clostridium] josui hits
..... unclassified Clostridiales	24	12	
...... Flavonifractor	1	11	Flavonifractor hits
.... Flavonifractor sp. An4	2	1	Flavonifractor sp. An4 hits
.... Flavonifractor plautii	7	3	Flavonifractor plautii hits
.... Flavonifractor plautii ATCC 29863	1	1	Flavonifractor plautii ATCC 29863 hits
.... Clostridium orbiscindens 1_3_50AFAA	1	1	Clostridium orbiscindens 1_3_50AFAA hits
.... Flavonifractor sp. An112	2	1	Flavonifractor sp. An112 hits
.... uncultured Flavonifractor sp.	1	1	uncultured Flavonifractor sp. hits
.... Flavonifractor sp. An91	2	1	Flavonifractor sp. An91 hits
.... Flavonifractor sp. An52	2	1	Flavonifractor sp. An52 hits
.... Flavonifractor sp. An92	1	1	Flavonifractor sp. An92 hits

.... Flavonifractor sp. An135	1	1	Flavonifractor sp. An135 hits
...... Intestinimonas butyriciproducens	3	1	Intestinimonas butyriciproducens hits
..... Peptostreptococcaceae	4	2	
...... Clostridioides difficile	3	1	Clostridioides difficile hits
...... [Clostridium] dakarense	1	1	[Clostridium] dakarense hits
..... Lachnospiraceae bacterium 7_1_58FAA	1	1	Lachnospiraceae bacterium 7_1_58FAA hits
..... Clostridium	3	2	
...... Clostridium sp. ATCC BAA-442	1	1	Clostridium sp. ATCC BAA-442 hits
...... uncultured Clostridium sp.	2	1	uncultured Clostridium sp. hits
... Erysipelotrichaceae	4	3	
.... Massiliomicrobiota sp. An142	2	1	Massiliomicrobiota sp. An142 hits
.... Coprobacillus	1	2	Coprobacillus hits
..... Coprobacillus sp. 29_1	1	1	Coprobacillus sp. 29_1 hits

BLAST is a registered trademark of the National Library of Medicine

Support center Mailing list YouTube

- NATIONAL LIBRARY OF MEDICINE National Library Of Medicine
- National Institutes Of Health
- U.S. Department of Health & Human Services
- USA.gov

NCBI

National Center for Biotechnology Information, U.S. National Library of Medicine 8600 Rockville Pike, Bethesda MD, 20894 USA

Policies and Guidelines | Contact
