# Supplementary material for: The Protozoan Trichomonas vaginalis Targets Bacteria with Laterally Acquired NlpC/P60 Peptidoglycan Hydrolases
Source: mBio. 2018 Dec 11;9(6):e01784-18. doi: 10.1128/mBio.01784-18 (PMC6299479; doi:10.1128/mBio.01784-18)
Supplement: TABLE S3 [file mbo006184213st3.pdf]

Tax BLAST report

Tax BLAST report

**RID**	P7T8ZH6H014 (Expires on 07-12 04:35 am)
**Query ID**	XP_001326856.1
**Description**	Clan CA, family C40, NlpC/P60 superfamily cysteine peptidase [Trichomonas vaginalis G3]
**Molecule type**	amino acid
**Query Length**	141
**Database Name**	nr
**Description**	All non-redundant GenBank CDS translations+PDB+SwissProt+ excluding environmental samples from WGS projects
**Program**	BLASTP 2.6.1+

Lineage Report

Organism Report Taxonomy Report

Organism	Blast Name	Score	Number of Hits	Description
root			225	
.cellular organisms			214	
..Trichomonas vaginalis G3	trichomonads	292	8	**Trichomonas vaginalis G3 hits**
..Corynebacterium sp. HMSC074C03	high GC Gram+	113	2	**Corynebacterium sp. HMSC074C03 hits**
..Corynebacterium sp. HMSC073B01	high GC Gram+	112	2	**Corynebacterium sp. HMSC073B01 hits**
..Corynebacterium sp. HMSC064E10	high GC Gram+	112	2	**Corynebacterium sp. HMSC064E10 hits**
..Corynebacterium sp. HMSC061H03	high GC Gram+	112	2	**Corynebacterium sp. HMSC061H03 hits**
..Corynebacterium	high GC Gram+	112	2	**Corynebacterium hits**
..Corynebacterium sp. HFH0082	high GC Gram+	112	1	**Corynebacterium sp. HFH0082 hits**
..Corynebacterium sp. ATCC 6931	high GC Gram+	112	1	**Corynebacterium sp. ATCC 6931 hits**
..Corynebacterium sp. HMSC063A05	high GC Gram+	112	1	**Corynebacterium sp. HMSC063A05 hits**
..Corynebacterium sp. HMSC074C11	high GC Gram+	112	1	**Corynebacterium sp. HMSC074C11 hits**
..Corynebacterium sp. HMSC11H10	high GC Gram+	112	1	**Corynebacterium sp. HMSC11H10 hits**
..Corynebacterium sp. HMSC074C04	high GC Gram+	112	1	**Corynebacterium sp. HMSC074C04 hits**
..Corynebacterium amycolatum	high GC Gram+	112	3	**Corynebacterium amycolatum hits**
..Corynebacterium sp.	high GC			**Corynebacterium sp. HMSC076C10**

HMSC076C10	Gram+	112	1	hits
..Corynebacterium sp. HMSC064E08	high GC Gram+	112	1	**Corynebacterium sp. HMSC064E08 hits**
..Corynebacterium sp. HMSC063F04	high GC Gram+	112	1	**Corynebacterium sp. HMSC063F04 hits**
..Corynebacterium sp. HMSC063G05	high GC Gram+	112	1	**Corynebacterium sp. HMSC063G05 hits**
..Corynebacterium sp. HMSC077C02	high GC Gram+	112	1	**Corynebacterium sp. HMSC077C02 hits**
..Corynebacterium sp. HMSC077G01	high GC Gram+	112	1	**Corynebacterium sp. HMSC077G01 hits**
..Corynebacterium sp. HMSC064H12	high GC Gram+	112	1	**Corynebacterium sp. HMSC064H12 hits**
..Corynebacterium sp. HMSC077G07	high GC Gram+	112	1	**Corynebacterium sp. HMSC077G07 hits**
..Corynebacterium sp. HMSC055G02	high GC Gram+	112	1	**Corynebacterium sp. HMSC055G02 hits**
..Corynebacterium sp. HMSC064E07	high GC Gram+	112	1	**Corynebacterium sp. HMSC064E07 hits**
..Corynebacterium sp. HMSC070B05	high GC Gram+	112	1	**Corynebacterium sp. HMSC070B05 hits**
..Corynebacterium sp. HMSC065H09	high GC Gram+	112	1	**Corynebacterium sp. HMSC065H09 hits**
..Corynebacterium sp. HMSC14B06	high GC Gram+	112	1	**Corynebacterium sp. HMSC14B06 hits**
..Corynebacterium sp. HMSC14H10	high GC Gram+	112	1	**Corynebacterium sp. HMSC14H10 hits**
..Corynebacterium sp. HMSC072B08	high GC Gram+	112	1	**Corynebacterium sp. HMSC072B08 hits**
..Corynebacterium sp. HMSC072B09	high GC Gram+	112	1	**Corynebacterium sp. HMSC072B09 hits**
..Corynebacterium sp. HMSC072D01	high GC Gram+	112	1	**Corynebacterium sp. HMSC072D01 hits**
..Corynebacterium sp. HMSC075F02	high GC Gram+	112	1	**Corynebacterium sp. HMSC075F02 hits**
..Corynebacterium jeikeium	high GC Gram+	112	1	**Corynebacterium jeikeium hits**
..Corynebacterium sp. HMSC074C05	high GC Gram+	112	2	**Corynebacterium sp. HMSC074C05 hits**
..Corynebacterium sp. HMSC073H12	high GC Gram+	110	2	**Corynebacterium sp. HMSC073H12 hits**
..Corynebacterium amycolatum SK46	high GC Gram+	110	1	**Corynebacterium amycolatum SK46 hits**
..Corynebacterium vitaeruminis	high GC Gram+	110	1	**Corynebacterium vitaeruminis hits**
..Paraclostridium bifermentans ATCC 638	firmicutes	113	1	**Paraclostridium bifermentans ATCC 638 hits**
..Paraclostridium bifermentans	firmicutes	108	1	**Paraclostridium bifermentans hits**

..Amycolatopsis taiwanensis	high GC Gram+	108	1	Amycolatopsis taiwanensis hits
..Bacillus massiliogorillae	firmicutes	108	1	Bacillus massiliogorillae hits
..Streptomyces cellostaticus	high GC Gram+	107	2	Streptomyces cellostaticus hits
..Streptomyces	high GC Gram+	107	6	Streptomyces hits
..Streptomyces sp. PBH53	high GC Gram+	107	1	Streptomyces sp. PBH53 hits
..Clostridioides difficile	firmicutes	107	79	Clostridioides difficile hits
..Clostridioides difficile CD69	firmicutes	107	1	Clostridioides difficile CD69 hits
..Thermocrispum agreste	high GC Gram+	106	1	Thermocrispum agreste hits
..Streptomyces reticuli	high GC Gram+	105	1	Streptomyces reticuli hits
..Streptomyces regensis	high GC Gram+	103	2	Streptomyces regensis hits
..Corynebacterium sphenisci	high GC Gram+	101	1	Corynebacterium sphenisci hits
..Corynebacterium sphenisci DSM 44792	high GC Gram+	101	1	Corynebacterium sphenisci DSM 44792 hits
..Amycolatopsis thermoflava	high GC Gram+	105	1	Amycolatopsis thermoflava hits
..Amycolatopsis sp. ATCC 39116	high GC Gram+	104	1	Amycolatopsis sp. ATCC 39116 hits
..Streptomyces sp. NRRL WC-3744	high GC Gram+	104	1	Streptomyces sp. NRRL WC-3744 hits
..Streptomyces antibioticus	high GC Gram+	104	1	Streptomyces antibioticus hits
..Streptomyces sp. NRRL WC-3725	high GC Gram+	104	1	Streptomyces sp. NRRL WC-3725 hits
..Streptomyces collinus Tu 365	high GC Gram+	103	1	Streptomyces collinus Tu 365 hits
..Thermocrispum municipale	high GC Gram+	103	1	Thermocrispum municipale hits
..Prauserella rugosa	high GC Gram+	103	1	Prauserella rugosa hits
..Saccharomonospora sp. CUA-673	high GC Gram+	104	2	Saccharomonospora sp. CUA-673 hits
..Streptomyces collinus	high GC Gram+	103	1	Streptomyces collinus hits
..Prauserella sp. Am3	high GC Gram+	103	1	Prauserella sp. Am3 hits
..Streptomyces griseochromogenes	high GC Gram+	103	4	Streptomyces griseochromogenes hits
..Streptomyces achromogenes	high GC Gram+	103	1	Streptomyces achromogenes hits
..Clostridium tyrobutyricum	firmicutes	102	4	Clostridium tyrobutyricum hits
..Clostridium tyrobutyricum DIVETGP	firmicutes	102	1	Clostridium tyrobutyricum DIVETGP hits

..Amycolatopsis methanolica	high GC Gram+	103	1	Amycolatopsis methanolica hits
..Amycolatopsis methanolica 239	high GC Gram+	103	1	Amycolatopsis methanolica 239 hits
..Bacillus drentensis	firmicutes	104	1	Bacillus drentensis hits
..Clostridioides difficile 824	firmicutes	105	1	Clostridioides difficile 824 hits
..Clostridium sp. HMSC19A11	firmicutes	105	2	Clostridium sp. HMSC19A11 hits
..Streptomyces lavenduligriseus	high GC Gram+	103	1	Streptomyces lavenduligriseus hits
..Clostridioides difficile Y381	firmicutes	104	1	Clostridioides difficile Y381 hits
..Clostridioides difficile F548	firmicutes	104	1	Clostridioides difficile F548 hits
..Bacillus sp. OV166	firmicutes	103	2	Bacillus sp. OV166 hits
..Clostridioides difficile E9	firmicutes	103	1	Clostridioides difficile E9 hits
..Corynebacterium lactis	high GC Gram+	99.0	1	Corynebacterium lactis hits
..Corynebacterium lactis RW2-5	high GC Gram+	99.0	1	Corynebacterium lactis RW2-5 hits
..Streptomyces sp. 142MFCol3.1	high GC Gram+	102	1	Streptomyces sp. 142MFCol3.1 hits
..Bacillus massilioanorexius	firmicutes	101	1	Bacillus massilioanorexius hits
..Clostridioides difficile T22	firmicutes	96.7	1	Clostridioides difficile T22 hits
..Clostridioides difficile T15	firmicutes	96.7	1	Clostridioides difficile T15 hits
..Streptomyces davawensis	high GC Gram+	102	1	Streptomyces davawensis hits
..Streptomyces davawensis JCM 4913	high GC Gram+	102	1	Streptomyces davawensis JCM 4913 hits
..Streptomyces sp. SolWspMP-5a-2	high GC Gram+	101	1	Streptomyces sp. SolWspMP-5a-2 hits
..Clostridioides difficile P73	firmicutes	103	1	Clostridioides difficile P73 hits
..Intrasporangium calvum	high GC Gram+	100	1	Intrasporangium calvum hits
..Intrasporangium calvum DSM 43043	high GC Gram+	100	1	Intrasporangium calvum DSM 43043 hits
..Streptomyces emeiensis	high GC Gram+	101	1	Streptomyces emeiensis hits
..Clostridioides difficile P7	firmicutes	103	1	Clostridioides difficile P7 hits
..Clostridioides difficile P70	firmicutes	103	1	Clostridioides difficile P70 hits
..Clostridium scatologenes	firmicutes	100	2	Clostridium scatologenes hits
..Actinoalloteichus spitiensis	high GC Gram+	100	1	Actinoalloteichus spitiensis hits
..Streptomyces sp. H-KF8	high GC Gram+	100	2	Streptomyces sp. H-KF8 hits
..Streptomyces bambergiensis	high GC Gram+	100	1	Streptomyces bambergiensis hits
..Lactobacillus reuteri	firmicutes	102	6	Lactobacillus reuteri hits
..Streptomyces sp. CC71	high GC Gram+	100	1	Streptomyces sp. CC71 hits

..Clostridioides difficile Y343	firmicutes	102	1	Clostridioides difficile Y343 hits
.Clostridium phage phiCDHM11	viruses	105	2	Clostridium phage phiCDHM11 hits
.Clostridium phage phiCDHM14	viruses	105	1	Clostridium phage phiCDHM14 hits
.Clostridium phage phiCDHM13	viruses	105	2	Clostridium phage phiCDHM13 hits
.Clostridium phage phiCD506	viruses	104	2	Clostridium phage phiCD506 hits
.Clostridium phage phiCDHM19	viruses	104	2	Clostridium phage phiCDHM19 hits
.Clostridium phage phiCD481-1	viruses	104	2	Clostridium phage phiCD481-1 hits

Organism Report

Lineage Report Taxonomy Report

Description	Score	E value	Accession
Trichomonas vaginalis G3 [trichomonads] Next Previous First			
Clan CA, family C40, NlpC/P60 superfamily cysteine peptidase [Trichomonas vaginalis G3]	292	2e-100	XP_001326856
Clan CA, family C40, NlpC/P60 superfamily cysteine peptidase [Trichomonas vaginalis G3]	292	2e-100	EAY14633
Clan CA, family C40, NlpC/P60 superfamily cysteine peptidase [Trichomonas vaginalis G3]	186	2e-58	XP_001321089
Clan CA, family C40, NlpC/P60 superfamily cysteine peptidase [Trichomonas vaginalis G3]	186	2e-58	EAY08866
Clan CA, family C40, NlpC/P60 superfamily cysteine peptidase [Trichomonas vaginalis G3]	125	2e-34	XP_001310764
Clan CA, family C40, NlpC/P60 superfamily cysteine peptidase [Trichomonas vaginalis G3]	125	2e-34	EAX97834
Clan CA, family C40, NlpC/P60 superfamily cysteine peptidase [Trichomonas vaginalis G3]	123	1e-33	XP_001327845
Clan CA, family C40, NlpC/P60 superfamily cysteine peptidase [Trichomonas vaginalis G3]	123	1e-33	EAY15622
Corynebacterium sp. HMSC074C03 [high GC Gram+] Next Previous First			
peptidoglycan endopeptidase [Corynebacterium sp. HMSC074C03]	113	6e-29	WP_070851381

hypothetical protein HMPREF2847_02230 [Corynebacterium sp. HMSC074C03]	113	6e-29	OHR35939

Corynebacterium sp. HMSC073B01
[
high GC Gram+
]
Next
Previous
First

peptidoglycan endopeptidase [Corynebacterium sp. HMSC073B01]	112	2e-28	WP_070862925
hypothetical protein HMPREF2849_09675 [Corynebacterium sp. HMSC073B01]	112	2e-28	OHR30011

Corynebacterium sp. HMSC064E10
[
high GC Gram+
]
Next
Previous
First

peptidoglycan endopeptidase [Corynebacterium sp. HMSC064E10]	112	2e-28	WP_070628870
hypothetical protein HMPREF2860_09950 [Corynebacterium sp. HMSC064E10]	112	2e-28	OFR94376

Corynebacterium sp. HMSC061H03
[
high GC Gram+
]
Next
Previous
First

peptidoglycan endopeptidase [Corynebacterium sp. HMSC061H03]	112	2e-28	WP_070858913
hypothetical protein HMPREF2787_07510 [Corynebacterium sp. HMSC061H03]	112	2e-28	OHR21753

Corynebacterium
[
high GC Gram+
]
Next
Previous
First

MULTISPECIES: peptidoglycan endopeptidase [Corynebacterium]	112	2e-28	WP_016423023
MULTISPECIES: peptidoglycan endopeptidase [Corynebacterium]	112	2e-28	WP_048731217

Corynebacterium sp. HFH0082
[
high GC Gram+
]
Next
Previous
First

hypothetical protein HMPREF1206_01852 [Corynebacterium sp. HFH0082]	112	2e-28	EPD45662

Corynebacterium sp. ATCC 6931
[
high GC Gram+

]
Next
Previous
First

hypothetical protein DR71_317 [Corynebacterium sp. ATCC 6931]	112	2e-28	AIN81259

Corynebacterium sp. HMSC063A05
[
high GC Gram+
]
Next
Previous
First

hypothetical protein HMPREF2651_09340 [Corynebacterium sp. HMSC063A05]	112	2e-28	OFM83597

Corynebacterium sp. HMSC074C11
[
high GC Gram+
]
Next
Previous
First

hypothetical protein HMPREF2614_06680 [Corynebacterium sp. HMSC074C11]	112	2e-28	OFN07583

Corynebacterium sp. HMSC11H10
[
high GC Gram+
]
Next
Previous
First

hypothetical protein HMPREF3122_07480 [Corynebacterium sp. HMSC11H10]	112	2e-28	OFU55251

Corynebacterium sp. HMSC074C04
[
high GC Gram+
]
Next
Previous
First

hypothetical protein HMPREF3011_00910 [Corynebacterium sp. HMSC074C04]	112	2e-28	OHR36972

Corynebacterium amycolatum
[
high GC Gram+
]
Next
Previous
First

hypothetical protein BXT90_01320 [Corynebacterium amycolatum]	112	2e-28	OMQ10706
hypothetical protein BGC22_02615 [Corynebacterium amycolatum]	112	2e-28	ODQ42712
peptidoglycan endopeptidase [Corynebacterium amycolatum]	110	1e-27	WP_005510412

Corynebacterium sp. HMSC076C10
[
high GC Gram+
]
Next

Previous<br>First			
hypothetical protein HMPREF2857_01660 [Corynebacterium sp. HMSC076C10]<br>Corynebacterium sp. HMSC064E08<br>[<br>high GC Gram+<br>]<br>Next<br>Previous<br>First	112	2e-28	OFJ57652
hypothetical protein HMPREF2820_04855 [Corynebacterium sp. HMSC064E08]<br>Corynebacterium sp. HMSC063F04<br>[<br>high GC Gram+<br>]<br>Next<br>Previous<br>First	112	2e-28	OFK32867
hypothetical protein HMPREF2788_00715 [Corynebacterium sp. HMSC063F04]<br>Corynebacterium sp. HMSC063G05<br>[<br>high GC Gram+<br>]<br>Next<br>Previous<br>First	112	2e-28	OFL11991
hypothetical protein HMPREF2751_05245 [Corynebacterium sp. HMSC063G05]<br>Corynebacterium sp. HMSC077C02<br>[<br>high GC Gram+<br>]<br>Next<br>Previous<br>First	112	2e-28	OFL71165
hypothetical protein HMPREF2752_04815 [Corynebacterium sp. HMSC077C02]<br>Corynebacterium sp. HMSC077G01<br>[<br>high GC Gram+<br>]<br>Next<br>Previous<br>First	112	2e-28	OFL75652
hypothetical protein HMPREF2714_00635 [Corynebacterium sp. HMSC077G01]<br>Corynebacterium sp. HMSC064H12<br>[<br>high GC Gram+<br>]<br>Next<br>Previous<br>First	112	2e-28	OFM15842
hypothetical protein HMPREF2681_00540 [Corynebacterium sp. HMSC064H12]<br>Corynebacterium sp. HMSC077G07<br>[	112	2e-28	OFM49007

high GC Gram+
]
Next
Previous
First

hypothetical protein HMPREF2562_07265 [Corynebacterium sp. HMSC077G07]<br>Corynebacterium sp. HMSC055G02<br>[<br>high GC Gram+<br>]<br>Next<br>Previous<br>First	112	2e-28	OFN35599
hypothetical protein HMPREF2547_09370 [Corynebacterium sp. HMSC055G02]<br>Corynebacterium sp. HMSC064E07<br>[<br>high GC Gram+<br>]<br>Next<br>Previous<br>First	112	2e-28	OFN54571
hypothetical protein HMPREF3053_03600 [Corynebacterium sp. HMSC064E07]<br>Corynebacterium sp. HMSC070B05<br>[<br>high GC Gram+<br>]<br>Next<br>Previous<br>First	112	2e-28	OFO25264
hypothetical protein HMPREF2960_09805 [Corynebacterium sp. HMSC070B05]<br>Corynebacterium sp. HMSC065H09<br>[<br>high GC Gram+<br>]<br>Next<br>Previous<br>First	112	2e-28	OFQ03582
hypothetical protein HMPREF2878_02050 [Corynebacterium sp. HMSC065H09]<br>Corynebacterium sp. HMSC14B06<br>[<br>high GC Gram+<br>]<br>Next<br>Previous<br>First	112	2e-28	OFR58803
hypothetical protein HMPREF3130_06130 [Corynebacterium sp. HMSC14B06]<br>Corynebacterium sp. HMSC14H10<br>[<br>high GC Gram+<br>]<br>Next<br>Previous<br>First	112	2e-28	OFT70769
hypothetical protein HMPREF3135_05485 [Corynebacterium sp.	112	2e-28	OFU61323

HMSC14H10]			
Corynebacterium sp. HMSC072B08 [high GC Gram+] Next Previous First			
hypothetical protein HMPREF2657_00330 [Corynebacterium sp. HMSC072B08]	112	2e-28	OHQ65603
Corynebacterium sp. HMSC072B09 [high GC Gram+] Next Previous First			
hypothetical protein HMPREF2985_09710 [Corynebacterium sp. HMSC072B09]	112	2e-28	OHR26555
Corynebacterium sp. HMSC072D01 [high GC Gram+] Next Previous First			
hypothetical protein HMPREF2899_08345 [Corynebacterium sp. HMSC072D01]	112	2e-28	OHR28358
Corynebacterium sp. HMSC075F02 [high GC Gram+] Next Previous First			
hypothetical protein HMPREF2920_05960 [Corynebacterium sp. HMSC075F02]	112	2e-28	OHR37838
Corynebacterium jeikeium [high GC Gram+] Next Previous First			
peptidoglycan endopeptidase [Corynebacterium jeikeium]	112	2e-28	ASE56217
Corynebacterium sp. HMSC074C05 [high GC Gram+] Next Previous First			
peptidoglycan endopeptidase [Corynebacterium sp. HMSC074C05]	112	2e-28	WP_070856422
hypothetical protein HMPREF3042_05520 [Corynebacterium sp. HMSC074C05]	112	2e-28	OHR32824
Corynebacterium sp. HMSC073H12 [high GC Gram+]			

Next
Previous
First

peptidoglycan endopeptidase [Corynebacterium sp. HMSC073H12]	110	1e-27	WP_070833969
hypothetical protein HMPREF2708_08920 [Corynebacterium sp. HMSC073H12]	110	1e-27	OHQ78704

Corynebacterium amycolatum SK46
[
high GC Gram+
]
Next
Previous
First

NlpC/P60 family protein [Corynebacterium amycolatum SK46]	110	1e-27	EEB63538

Corynebacterium vitaeruminis
[
high GC Gram+
]
Next
Previous
First

peptidoglycan endopeptidase [Corynebacterium vitaeruminis]	110	1e-27	WP_048759003

Clostridium bifermentans ATCC 638
[
firmicutes
]
Next
Previous
First

nlpC/P60 family protein [[Clostridium] bifermentans ATCC 638]	113	2e-26	EQK42778

[Clostridium] bifermentans
[
firmicutes
]
Next
Previous
First

hypothetical protein [Paraclostridium bifermentans]	108	2e-26	WP_082435333

Amycolatopsis taiwanensis
[
high GC Gram+
]
Next
Previous
First

hydrolase Nlp/P60 [Amycolatopsis taiwanensis]	108	9e-26	WP_027943279

Bacillus sp. G2(2012b)
[
firmicutes
]
Next
Previous
First

hypothetical protein [Bacillus massiliogorillae]	108	1e-25	WP_042345496

Streptomyces cellostaticus
[
high GC Gram+
]
Next
Previous
First

hypothetical protein [Streptomyces cellostaticus]	107	3e-25	WP_066992824
hypothetical protein AQI88_05725 [Streptomyces cellostaticus]	107	3e-25	KUM98011

Streptomyces
[
high GC Gram+
]
Next
Previous
First

MULTISPECIES: hypothetical protein [Streptomyces]	107	4e-25	WP_030348430
MULTISPECIES: hypothetical protein [Streptomyces]	105	1e-24	WP_059248878
MULTISPECIES: hypothetical protein [Streptomyces]	104	4e-24	WP_030641925
MULTISPECIES: hypothetical protein [Streptomyces]	101	6e-23	WP_055573823
MULTISPECIES: hypothetical protein [Streptomyces]	101	8e-23	WP_055596073
MULTISPECIES: hypothetical protein [Streptomyces]	100	1e-22	WP_029393871

Streptomyces sp. PBH53
[
high GC Gram+
]
Next
Previous
First

hypothetical protein QR97_29170 [Streptomyces sp. PBH53]	107	5e-25	AKN73289

[Clostridium] difficile
[
firmicutes
]
Next
Previous
First

cell wall hydrolase [Clostridioides difficile]	107	1e-24	WP_074115320
cell wall hydrolase [Clostridioides difficile]	107	1e-24	WP_065987067
phage cell wall hydrolase [Clostridioides difficile]	107	1e-24	SJV37295
phage cell wall hydrolase [Clostridioides difficile]	107	1e-24	SJV79580
phage cell wall hydrolase [Clostridioides difficile]	107	1e-24	SJV30551
phage cell wall hydrolase [Clostridioides difficile]	107	1e-24	SJW06095
nlpC/P60 family protein [Clostridioides difficile]	107	1e-24	WP_021374754
Gamma-D-glutamyl-L-lysine endopeptidase [Clostridioides difficile]	105	8e-24	SJR46286
Gamma-D-glutamyl-L-lysine endopeptidase [Clostridioides difficile]	105	8e-24	SJT29967
Gamma-D-glutamyl-L-lysine endopeptidase [Clostridioides difficile]	105	8e-24	SJS43855
Gamma-D-glutamyl-L-lysine endopeptidase [Clostridioides difficile]	105	8e-24	SJQ97483

Gamma-D-glutamyl-L-lysine endopeptidase [Clostridioides difficile]	105	8e-24	SJP63004
Gamma-D-glutamyl-L-lysine endopeptidase [Clostridioides difficile]	105	8e-24	SJT05040
Gamma-D-glutamyl-L-lysine endopeptidase [Clostridioides difficile]	105	8e-24	SJO64387
cell wall hydrolase [Clostridioides difficile]	105	1e-23	WP_042741807
bacteriophage peptidoglycan hydrolase [Clostridioides difficile]	105	1e-23	WP_021391021
putative phage cell wall hydrolase [Clostridioides difficile]	105	1e-23	CDS92633
Gamma-D-glutamyl-L-lysine endopeptidase [Clostridioides difficile]	105	1e-23	SJO71262
Gamma-D-glutamyl-L-lysine endopeptidase [Clostridioides difficile]	105	1e-23	SJO83757
Gamma-D-glutamyl-L-lysine endopeptidase [Clostridioides difficile]	105	1e-23	SJR41359
Gamma-D-glutamyl-L-lysine endopeptidase [Clostridioides difficile]	105	1e-23	SJT09439
Gamma-D-glutamyl-L-lysine endopeptidase [Clostridioides difficile]	105	1e-23	SKA47063
cell wall hydrolase [Clostridioides difficile]	105	1e-23	WP_074037494
cell wall hydrolase [Clostridioides difficile]	104	1e-23	WP_074145892
cell wall hydrolase [Clostridioides difficile]	104	1e-23	WP_085065370
cell wall hydrolase [Clostridioides difficile]	104	1e-23	WP_074183854
Gamma-D-glutamyl-L-lysine endopeptidase [Clostridioides difficile]	104	1e-23	CZR81472
cell wall hydrolase [Clostridioides difficile]	104	1e-23	WP_077745822
Gamma-D-glutamyl-L-lysine endopeptidase [Clostridioides difficile]	104	1e-23	SJO77624
nlpC/P60 family protein [Clostridioides difficile]	104	1e-23	WP_021414877
cell wall hydrolase [Clostridioides difficile]	104	1e-23	WP_074097922
Gamma-D-glutamyl-L-lysine endopeptidase [Clostridioides difficile]	104	1e-23	SJU01698
Gamma-D-glutamyl-L-lysine endopeptidase [Clostridioides difficile]	104	1e-23	SJT30418
hypothetical protein BW28_13685, partial [Clostridioides difficile]	97.8	1e-23	OFA20416
cell wall hydrolase [Clostridioides difficile]	104	1e-23	WP_077715712
Gamma-D-glutamyl-L-lysine endopeptidase [Clostridioides difficile]	104	1e-23	SJU35907
bacteriophage peptidoglycan hydrolase [Clostridioides difficile]	104	1e-23	WP_021435021
putative phage cell wall hydrolase [Clostridioides difficile]	104	1e-23	CDS82794
cell wall hydrolase [Clostridioides difficile]	104	1e-23	WP_059027011
cell wall hydrolase [Clostridioides difficile]	104	1e-23	WP_074074700
NlpC/P60 family protein [Clostridioides difficile]	104	1e-23	CDS82749
Gamma-D-glutamyl-L-lysine endopeptidase [Clostridioides	104	1e-23	SJP13975

difficile]			
Murein DD-endopeptidase MepH precursor [Clostridioides difficile]	104	1e-23	ALP03616
Murein DD-endopeptidase MepH precursor [Clostridioides difficile]	104	1e-23	ALP03669
cell wall hydrolase [Clostridioides difficile]	104	2e-23	WP_075042982
cell wall hydrolase [Clostridioides difficile]	104	2e-23	WP_077727483
Gamma-D-glutamyl-L-lysine endopeptidase [Clostridioides difficile]	104	2e-23	SJQ73073
Gamma-D-glutamyl-L-lysine endopeptidase [Clostridioides difficile]	104	2e-23	SJP22131
Gamma-D-glutamyl-L-lysine endopeptidase [Clostridioides difficile]	104	2e-23	SJR65090
Gamma-D-glutamyl-L-lysine endopeptidase [Clostridioides difficile]	104	2e-23	SJP12015
Gamma-D-glutamyl-L-lysine endopeptidase [Clostridioides difficile]	104	2e-23	SJS99629
Gamma-D-glutamyl-L-lysine endopeptidase [Clostridioides difficile]	104	2e-23	SJQ73887
Gamma-D-glutamyl-L-lysine endopeptidase [Clostridioides difficile]	104	2e-23	SJS50083
Gamma-D-glutamyl-L-lysine endopeptidase [Clostridioides difficile]	104	2e-23	SJR83465
Gamma-D-glutamyl-L-lysine endopeptidase [Clostridioides difficile]	104	2e-23	SJO68158
Gamma-D-glutamyl-L-lysine endopeptidase [Clostridioides difficile]	104	2e-23	SJP45747
Gamma-D-glutamyl-L-lysine endopeptidase [Clostridioides difficile]	104	2e-23	SJS14477
Gamma-D-glutamyl-L-lysine endopeptidase [Clostridioides difficile]	104	2e-23	SKA46498
cell wall hydrolase [Clostridioides difficile]	104	2e-23	WP_074074660
cell wall hydrolase [Clostridioides difficile]	104	2e-23	WP_077738065
Gamma-D-glutamyl-L-lysine endopeptidase [Clostridioides difficile]	104	2e-23	SJP67482
cell wall hydrolase [Clostridioides difficile]	103	2e-23	WP_074136133
Gamma-D-glutamyl-L-lysine endopeptidase [Clostridioides difficile]	101	3e-23	SJR62350
phage cell wall hydrolase [Clostridioides difficile]	103	3e-23	SJW17689
mannosyl-glycoendo-beta-N-acetylglucosaminidase, partial [Clostridioides difficile]	103	4e-23	WP_021427575
putative endopeptidase precursor [Clostridioides difficile]	99.0	5e-23	ALP05131
cell wall hydrolase [Clostridioides difficile]	103	6e-23	WP_074048434
mannosyl-glycoendo-beta-N-acetylglucosaminidase [Clostridioides difficile]	103	6e-23	WP_021417117
phage cell wall hydrolase [Clostridioides difficile]	103	6e-23	SJU86995
phage cell wall hydrolase [Clostridioides difficile]	103	6e-23	SJU75590

phage cell wall hydrolase [Clostridioides difficile]	103	6e-23	SJV03632
Murein DD-endopeptidase MepH precursor [Clostridioides difficile]	98.6	8e-23	CZS06512
hypothetical protein BG47_19135, partial [Clostridioides difficile]	99.0	9e-23	EZR26962
cell wall hydrolase [Clostridioides difficile]	102	1e-22	WP_077724957
Probable endopeptidase cgR_2070 precursor [Clostridioides difficile]	102	1e-22	SJR58485
cell wall hydrolase, partial [Clostridioides difficile]	102	1e-22	WP_074075764
mannosyl-glycoendo-beta-N-acetylglucosaminidase, partial [Clostridioides difficile]	102	1e-22	WP_021414299
cell wall hydrolase [Clostridioides difficile]	102	1e-22	WP_074439410
cell wall hydrolase [Clostridioides difficile]	102	1e-22	WP_074097967
Clostridium difficile CD69 [firmicutes] Next Previous First			
nlpC/P60 family protein [Clostridioides difficile CD69]	107	1e-24	EQE87805
Thermocrispum agreste [high GC Gram+] Next Previous First			
hypothetical protein [Thermocrispum agreste]	106	1e-24	WP_084609252
Streptomyces reticuli [high GC Gram+] Next Previous First			
putative endopeptidase precursor [Streptomyces reticuli]	105	1e-24	CUW27937
Streptomyces regensis [high GC Gram+] Next Previous First			
hydrolase Nlp/P60, partial [Streptomyces regensis]	103	2e-24	KMS84375
hypothetical protein ACZ91_31230 [Streptomyces regensis]	104	4e-24	KMS87393
Corynebacterium sphenisci [high GC Gram+] Next Previous First			
hypothetical protein [Corynebacterium sphenisci]	101	2e-24	WP_075692269
Corynebacterium sphenisci DSM 44792 [			

high GC Gram+<br>]<br>Next<br>Previous<br>First			
**hypothetical protein CSPHI_07980 [Corynebacterium sphenisci DSM 44792]**<br><br>Amycolatopsis thermoflava<br>[<br>high GC Gram+<br>]<br>Next<br>Previous<br>First	101	2e-24	**APT90983**
**hydrolase Nlp/P60 [Amycolatopsis thermoflava]**<br><br>Amycolatopsis sp. ATCC 39116<br>[<br>high GC Gram+<br>]<br>Next<br>Previous<br>First	105	2e-24	**WP_027934794**
**hydrolase Nlp/P60 [Amycolatopsis sp. ATCC 39116]**<br><br>Streptomyces sp. NRRL WC-3744<br>[<br>high GC Gram+<br>]<br>Next<br>Previous<br>First	104	3e-24	**WP_020421858**
**hypothetical protein [Streptomyces sp. NRRL WC-3744]**<br><br>Streptomyces antibioticus<br>[<br>high GC Gram+<br>]<br>Next<br>Previous<br>First	104	4e-24	**WP_030992645**
**hypothetical protein ADK77_24965 [Streptomyces antibioticus]**<br><br>Streptomyces sp. NRRL WC-3725<br>[<br>high GC Gram+<br>]<br>Next<br>Previous<br>First	104	4e-24	**KOG63209**
**hypothetical protein [Streptomyces sp. NRRL WC-3725]**<br><br>Streptomyces collinus Tu 365<br>[<br>high GC Gram+<br>]<br>Next<br>Previous<br>First	104	4e-24	**WP_031024246**
**hypothetical protein B446_32150 [Streptomyces collinus Tu 365]**<br><br>Thermocrispum municipale<br>[<br>high GC Gram+	103	5e-24	**AGS73234**

]
Next
Previous
First

hypothetical protein [Thermocrispum municipale]	103	6e-24	WP_084612270

Prauserella rugosa
[
high GC Gram+
]
Next
Previous
First

hydrolase NlpP60 [Prauserella rugosa]	103	6e-24	WP_084705694

Saccharomonospora sp. CUA-673
[
high GC Gram+
]
Next
Previous
First

hydrolase NlpP60 [Saccharomonospora sp. CUA-673]	104	7e-24	WP_075847257
hydrolase NlpP60 [Saccharomonospora sp. CUA-673]	104	7e-24	OLT45146

Streptomyces collinus
[
high GC Gram+
]
Next
Previous
First

hypothetical protein [Streptomyces collinus]	103	7e-24	WP_043476872

Prauserella sp. Am3
[
high GC Gram+
]
Next
Previous
First

cell wall-associated hydrolase, invasion-associated protein [Prauserella sp. Am3]	103	7e-24	KID30498

Streptomyces griseochromogenes
[
high GC Gram+
]
Next
Previous
First

hypothetical protein [Streptomyces griseochromogenes]	103	7e-24	WP_067313633
hypothetical protein AVL59_37710 [Streptomyces griseochromogenes]	103	7e-24	ANP54567
hypothetical protein [Streptomyces griseochromogenes]	100	1e-22	WP_067310171
hypothetical protein AVL59_29010 [Streptomyces griseochromogenes]	100	1e-22	ANP53044

Streptomyces achromogenes
[
high GC Gram+
]
Next
Previous
First

hypothetical protein [Streptomyces achromogenes]	103	7e-24	WP_030605617

Clostridium tyrobutyricum
[
firmicutes
]
Next
Previous
First

hypothetical protein [Clostridium tyrobutyricum]	102	8e-24	WP_017895682
hypothetical protein CTK_C10380 [Clostridium tyrobutyricum]	102	8e-24	AND84299
hypothetical protein CTK_C11220 [Clostridium tyrobutyricum]	102	8e-24	AND84383
hypothetical protein BA182_04775 [Clostridium tyrobutyricum]	102	8e-24	ANP69010

Clostridium tyrobutyricum DIVETGP
[
firmicutes
]
Next
Previous
First

putative cell wall-associated hydrolase [Clostridium tyrobutyricum DIVETGP]	102	8e-24	CDL91451

Clostridium phage phiCDHM11
[
viruses
]
Next
Previous
First

putative cell wall hydrolase protein [Clostridium phage phiCDHM11]	105	8e-24	YP_009217650
putative cell wall hydrolase protein [Clostridium phage phiCDHM11]	105	8e-24	CDL68832

Clostridium phage phiCDHM14
[
viruses
]
Next
Previous
First

putative cell wall hydrolase protein [Clostridium phage phiCDHM14]	105	8e-24	CDU85302

Amycolatopsis methanolica
[
high GC Gram+
]
Next
Previous
First

hydrolase Nlp/P60 [Amycolatopsis methanolica]	103	9e-24	WP_017984048

Amycolatopsis methanolica 239
[
high GC Gram+
]
Next
Previous

First

NLP/P60-family protein [Amycolatopsis methanolica 239]	103	9e-24	AIJ25209

Clostridium phage phiCDHM13
[
viruses
]
Next
Previous
First

putative cell wall hydrolase protein [Clostridium phage phiCDHM13]	105	1e-23	YP_009226587
putative cell wall hydrolase protein [Clostridium phage phiCDHM13]	105	1e-23	CDL65298

Bacillus drentensis
[
firmicutes
]
Next
Previous
First

hypothetical protein [Bacillus drentensis]	104	1e-23	WP_082797558

Clostridium difficile 824
[
firmicutes
]
Next
Previous
First

bacteriophage peptidoglycan hydrolase family protein [Clostridioides difficile 824]	105	1e-23	EQF92136

Clostridium sp. HMSC19A11
[
firmicutes
]
Next
Previous
First

cell wall hydrolase [Clostridium sp. HMSC19A11]	105	1e-23	WP_070470111
cell wall hydrolase [Clostridium sp. HMSC19A11]	105	1e-23	OFU49302

Streptomyces lavenduligriseus
[
high GC Gram+
]
Next
Previous
First

hypothetical protein [Streptomyces lavenduligriseus]	103	1e-23	WP_030783298

Clostridium difficile Y381
[
firmicutes
]
Next
Previous
First

nlpC/P60 family protein [Clostridioides difficile Y381]	104	1e-23	EQI71708

Clostridium phage phiCD506
[
viruses
]

Next
Previous
First

NlpC/P60 family protein [Clostridium phage phiCD506]	104	1e-23	YP_009202003
NlpC/P60 family protein [Clostridium phage phiCD506]	104	1e-23	CEK40717

Clostridium difficile F548
[
firmicutes
]
Next
Previous
First

bacteriophage peptidoglycan hydrolase family protein [Clostridioides difficile F548]	104	1e-23	EQK59165

Clostridium phage phiCDHM19
[
viruses
]
Next
Previous
First

putative cell wall hydrolase protein [Clostridium phage phiCDHM19]	104	1e-23	YP_009216876
putative cell wall hydrolase protein [Clostridium phage phiCDHM19]	104	1e-23	CDW17207

Clostridium phage phiCD481-1
[
viruses
]
Next
Previous
First

NlpC/P60 family protein [Clostridium phage phiCD481-1]	104	2e-23	YP_009213089
NlpC/P60 family protein [Clostridium phage phiCD481-1]	104	2e-23	CEK40587

Bacillus sp. OV166
[
firmicutes
]
Next
Previous
First

hypothetical protein [Bacillus sp. OV166]	103	2e-23	WP_088089720
Cell wall-associated hydrolase, NlpC family [Bacillus sp. OV166]	103	2e-23	SMQ84493

Clostridium difficile E9
[
firmicutes
]
Next
Previous
First

putative phage cell wall hydrolase [Clostridioides difficile E9]	103	2e-23	CCL62929

Corynebacterium lactis
[
high GC Gram+
]
Next
Previous

First			
hypothetical protein [Corynebacterium lactis]<br>Corynebacterium lactis RW2-5<br>[<br>high GC Gram+<br>]<br>Next<br>Previous<br>First	99.0	2e-23	WP_053412434
endopeptidase [Corynebacterium lactis RW2-5]<br>Streptomyces sp. 142MFCol3.1<br>[<br>high GC Gram+<br>]<br>Next<br>Previous<br>First	99.0	2e-23	ALA67665
hypothetical protein [Streptomyces sp. 142MFCol3.1]<br>Bacillus massilioanorexius<br>[<br>firmicutes<br>]<br>Next<br>Previous<br>First	102	3e-23	WP_028803249
hydrolase [Bacillus massilioanorexius]<br>Clostridium difficile T22<br>[<br>firmicutes<br>]<br>Next<br>Previous<br>First	101	3e-23	WP_087960631
Putative phage cell wall XkdQ-like hydrolase (fragment) [Clostridioides difficile T22]<br>Clostridium difficile T15<br>[<br>firmicutes<br>]<br>Next<br>Previous<br>First	96.7	3e-23	CCL16340
Putative phage cell wall XkdQ-like hydrolase (fragment) [Clostridioides difficile T15]<br>Streptomyces davawensis<br>[<br>high GC Gram+<br>]<br>Next<br>Previous<br>First	96.7	3e-23	CCL24583
secreted protein [Streptomyces davawensis]<br>Streptomyces davawensis JCM 4913<br>[<br>high GC Gram+<br>]<br>Next<br>Previous<br>First	102	3e-23	WP_015661010
secreted protein [Streptomyces davawensis JCM 4913]	102	3e-23	CCK30674

Streptomyces sp. SolWspMP-5a-2
[
high GC Gram+
]
Next
Previous
First

Cell wall-associated hydrolase, NlpC family [Streptomyces sp. SolWspMP-5a-2]	101	4e-23	SCD59993

Clostridium difficile P73
[
firmicutes
]
Next
Previous
First

mannosyl-glycoendo-beta-N-acetylglucosaminidase family protein, partial [Clostridioides difficile P73]	103	4e-23	EQK26406

Intrasporangium calvum
[
high GC Gram+
]
Next
Previous
First

NLP/P60 protein [Intrasporangium calvum]	100	5e-23	WP_013491620

Intrasporangium calvum DSM 43043
[
high GC Gram+
]
Next
Previous
First

NLP/P60 protein [Intrasporangium calvum DSM 43043]	100	5e-23	ADU47300

Streptomyces emeiensis
[
high GC Gram+
]
Next
Previous
First

Cell wall-associated hydrolase, NlpC family [Streptomyces emeiensis]	101	6e-23	SDC22697

Clostridium difficile P7
[
firmicutes
]
Next
Previous
First

mannosyl-glycoendo-beta-N-acetylglucosaminidase family protein [Clostridioides difficile P7]	103	6e-23	EQJ10742

Clostridium difficile P70
[
firmicutes
]
Next
Previous
First

mannosyl-glycoendo-beta-N-acetylglucosaminidase family protein [Clostridioides difficile P70]	103	6e-23	EQK15981
Clostridium scatologenes<br>[<br>firmicutes<br>]<br>Next<br>Previous<br>First			
hypothetical protein [Clostridium scatologenes]	100	7e-23	WP_082085138
transmembrane NLP/P60 family protein [Clostridium scatologenes]	100	7e-23	AKA71921
Actinoalloteichus spitiensis<br>[<br>high GC Gram+<br>]<br>Next<br>Previous<br>First			
hypothetical protein [Actinoalloteichus spitiensis]	100	9e-23	WP_016696682
Streptomyces sp. H-KF8<br>[<br>high GC Gram+<br>]<br>Next<br>Previous<br>First			
hypothetical protein [Streptomyces sp. H-KF8]	100	9e-23	WP_065004620
hypothetical protein A4U61_21375 [Streptomyces sp. H-KF8]	100	9e-23	OBQ49801
Streptomyces bambergiensis<br>[<br>high GC Gram+<br>]<br>Next<br>Previous<br>First			
hypothetical protein [Streptomyces bambergiensis]	100	1e-22	WP_055607712
Lactobacillus reuteri<br>[<br>firmicutes<br>]<br>Next<br>Previous<br>First			
hypothetical protein [Lactobacillus reuteri]	102	1e-22	WP_065867929
hypothetical protein BBP12_09490 [Lactobacillus reuteri]	102	1e-22	OCW61925
hypothetical protein BBP11_08570 [Lactobacillus reuteri]	102	1e-22	OCW62891
hypothetical protein BBP10_06065 [Lactobacillus reuteri]	102	1e-22	OCW63600
hypothetical protein BBP13_06145 [Lactobacillus reuteri]	102	1e-22	OCW69922
hypothetical protein BBP14_01915 [Lactobacillus reuteri]	102	1e-22	OCW69991
Streptomyces sp. CC71<br>[<br>high GC Gram+<br>]<br>Next<br>Previous<br>First			

hypothetical protein AUW26_07895 [Streptomyces sp. CC71]	100	1e-22	KYK10684
Clostridium difficile Y343 [firmicutes] Next Previous First			
mannosyl-glycoendo-beta-N-acetylglucosaminidase family protein, partial [Clostridioides difficile Y343]	102	1e-22	EQI68369

Taxonomy Report

Organism Report Lineage Report

Taxonomy	Number of hits	Number of Organisms	Description
root	225	103	
. cellular organisms	214	97	
.. Trichomonas vaginalis G3	8	1	Trichomonas vaginalis G3 hits
.. Terrabacteria group	206	96	
... Actinobacteria	94	73	
.... Corynebacteriaceae	48	39	
..... Corynebacterium	2	39	Corynebacterium hits
...... Corynebacterium sp. HMSC074C03	2	1	Corynebacterium sp. HMSC074C03 hits
...... Corynebacterium sp. HMSC073B01	2	1	Corynebacterium sp. HMSC073B01 hits
...... Corynebacterium sp. HMSC064E10	2	1	Corynebacterium sp. HMSC064E10 hits
...... Corynebacterium sp. HMSC061H03	2	1	Corynebacterium sp. HMSC061H03 hits
...... Corynebacterium sp. HFH0082	1	1	Corynebacterium sp. HFH0082 hits
...... Corynebacterium sp. ATCC 6931	1	1	Corynebacterium sp. ATCC 6931 hits
...... Corynebacterium sp. HMSC063A05	1	1	Corynebacterium sp. HMSC063A05 hits
...... Corynebacterium sp. HMSC074C11	1	1	Corynebacterium sp. HMSC074C11 hits
...... Corynebacterium sp. HMSC11H10	1	1	Corynebacterium sp. HMSC11H10 hits
...... Corynebacterium sp. HMSC074C04	1	1	Corynebacterium sp. HMSC074C04 hits
...... Corynebacterium amycolatum	3	2	Corynebacterium amycolatum hits
.... Corynebacterium amycolatum SK46	1	1	Corynebacterium amycolatum SK46 hits
...... Corynebacterium sp. HMSC076C10	1	1	Corynebacterium sp. HMSC076C10 hits

...... Corynebacterium sp. HMSC064E08	1	1	**Corynebacterium sp. HMSC064E08 hits**
...... Corynebacterium sp. HMSC063F04	1	1	**Corynebacterium sp. HMSC063F04 hits**
...... Corynebacterium sp. HMSC063G05	1	1	**Corynebacterium sp. HMSC063G05 hits**
...... Corynebacterium sp. HMSC077C02	1	1	**Corynebacterium sp. HMSC077C02 hits**
...... Corynebacterium sp. HMSC077G01	1	1	**Corynebacterium sp. HMSC077G01 hits**
...... Corynebacterium sp. HMSC064H12	1	1	**Corynebacterium sp. HMSC064H12 hits**
...... Corynebacterium sp. HMSC077G07	1	1	**Corynebacterium sp. HMSC077G07 hits**
...... Corynebacterium sp. HMSC055G02	1	1	**Corynebacterium sp. HMSC055G02 hits**
...... Corynebacterium sp. HMSC064E07	1	1	**Corynebacterium sp. HMSC064E07 hits**
...... Corynebacterium sp. HMSC070B05	1	1	**Corynebacterium sp. HMSC070B05 hits**
...... Corynebacterium sp. HMSC065H09	1	1	**Corynebacterium sp. HMSC065H09 hits**
...... Corynebacterium sp. HMSC14B06	1	1	**Corynebacterium sp. HMSC14B06 hits**
...... Corynebacterium sp. HMSC14H10	1	1	**Corynebacterium sp. HMSC14H10 hits**
...... Corynebacterium sp. HMSC072B08	1	1	**Corynebacterium sp. HMSC072B08 hits**
...... Corynebacterium sp. HMSC072B09	1	1	**Corynebacterium sp. HMSC072B09 hits**
...... Corynebacterium sp. HMSC072D01	1	1	**Corynebacterium sp. HMSC072D01 hits**
...... Corynebacterium sp. HMSC075F02	1	1	**Corynebacterium sp. HMSC075F02 hits**
...... Corynebacterium jeikeium	1	1	**Corynebacterium jeikeium hits**
...... Corynebacterium sp. HMSC074C05	2	1	**Corynebacterium sp. HMSC074C05 hits**
...... Corynebacterium sp. HMSC073H12	2	1	**Corynebacterium sp. HMSC073H12 hits**
...... Corynebacterium vitaeruminis	1	1	**Corynebacterium vitaeruminis hits**
...... Corynebacterium sphenisci	1	2	**Corynebacterium sphenisci hits**
.... Corynebacterium sphenisci DSM 44792	1	1	**Corynebacterium sphenisci DSM 44792 hits**
...... Corynebacterium lactis	1	2	**Corynebacterium lactis hits**
.... Corynebacterium lactis RW2-5	1	1	**Corynebacterium lactis RW2-5 hits**
.... Pseudonocardiaceae	12	11	
..... Amycolatopsis	5	5	

...... Amycolatopsis taiwanensis	1	1	Amycolatopsis taiwanensis hits
...... Amycolatopsis thermoflava	1	1	Amycolatopsis thermoflava hits
...... Amycolatopsis sp. ATCC 39116	1	1	Amycolatopsis sp. ATCC 39116 hits
...... Amycolatopsis methanolica	1	2	Amycolatopsis methanolica hits
.... Amycolatopsis methanolica 239	1	1	Amycolatopsis methanolica 239 hits
..... Thermocrispum	2	2	
...... Thermocrispum agreste	1	1	Thermocrispum agreste hits
...... Thermocrispum municipale	1	1	Thermocrispum municipale hits
..... Prauserella	2	2	
...... Prauserella rugosa	1	1	Prauserella rugosa hits
...... Prauserella sp. Am3	1	1	Prauserella sp. Am3 hits
..... Saccharomonospora sp. CUA-673	2	1	Saccharomonospora sp. CUA-673 hits
..... Actinoalloteichus spitiensis	1	1	Actinoalloteichus spitiensis hits
.... Streptomycetaceae	32	21	
..... Streptomyces	6	21	Streptomyces hits
...... Streptomyces cellostaticus	2	1	Streptomyces cellostaticus hits
...... Streptomyces sp. PBH53	1	1	Streptomyces sp. PBH53 hits
...... Streptomyces reticuli	1	1	Streptomyces reticuli hits
...... Streptomyces regensis	2	1	Streptomyces regensis hits
...... Streptomyces sp. NRRL WC-3744	1	1	Streptomyces sp. NRRL WC-3744 hits
...... Streptomyces antibioticus	1	1	Streptomyces antibioticus hits
...... Streptomyces sp. NRRL WC-3725	1	1	Streptomyces sp. NRRL WC-3725 hits
...... Streptomyces collinus	1	2	Streptomyces collinus hits
.... Streptomyces collinus Tu 365	1	1	Streptomyces collinus Tu 365 hits
...... Streptomyces griseochromogenes	4	1	Streptomyces griseochromogenes hits
...... Streptomyces achromogenes	1	1	Streptomyces achromogenes hits
...... Streptomyces lavenduligriseus	1	1	Streptomyces lavenduligriseus hits
...... Streptomyces sp. 142MFCol3.1	1	1	Streptomyces sp. 142MFCol3.1 hits
...... Streptomyces davawensis	1	2	Streptomyces davawensis hits
.... Streptomyces davawensis JCM 4913	1	1	Streptomyces davawensis JCM 4913 hits
...... Streptomyces sp. SolWspMP-5a-2	1	1	Streptomyces sp. SolWspMP-5a-2 hits
...... Streptomyces emeiensis	1	1	Streptomyces emeiensis hits
...... Streptomyces sp. H-KF8	2	1	Streptomyces sp. H-KF8 hits
...... Streptomyces bambergiensis	1	1	Streptomyces bambergiensis hits
...... Streptomyces sp. CC71	1	1	Streptomyces sp. CC71 hits
.... Intrasporangium	2	2	
..... Intrasporangium calvum	1	2	Intrasporangium calvum hits

...... Intrasporangium calvum DSM 43043	1	1	**Intrasporangium calvum DSM 43043 hits**
... Firmicutes	112	23	
.... Clostridiales	101	18	
..... Peptostreptococcaceae	92	14	
...... Paraclostridium	2	2	
.... Paraclostridium bifermentans	1	2	**Paraclostridium bifermentans hits**
.... Paraclostridium bifermentans ATCC 638	1	1	**Paraclostridium bifermentans ATCC 638 hits**
...... Clostridioides	90	12	
.... Clostridioides difficile	79	12	**Clostridioides difficile hits**
.... Clostridioides difficile CD69	1	1	**Clostridioides difficile CD69 hits**
.... Clostridioides difficile 824	1	1	**Clostridioides difficile 824 hits**
.... Clostridioides difficile Y381	1	1	**Clostridioides difficile Y381 hits**
.... Clostridioides difficile F548	1	1	**Clostridioides difficile F548 hits**
.... Clostridioides difficile E9	1	1	**Clostridioides difficile E9 hits**
.... Clostridioides difficile T22	1	1	**Clostridioides difficile T22 hits**
.... Clostridioides difficile T15	1	1	**Clostridioides difficile T15 hits**
.... Clostridioides difficile P73	1	1	**Clostridioides difficile P73 hits**
.... Clostridioides difficile P7	1	1	**Clostridioides difficile P7 hits**
.... Clostridioides difficile P70	1	1	**Clostridioides difficile P70 hits**
.... Clostridioides difficile Y343	1	1	**Clostridioides difficile Y343 hits**
..... Clostridium	9	4	
...... Clostridium tyrobutyricum	4	2	**Clostridium tyrobutyricum hits**
.... Clostridium tyrobutyricum DIVETGP	1	1	**Clostridium tyrobutyricum DIVETGP hits**
...... Clostridium sp. HMSC19A11	2	1	**Clostridium sp. HMSC19A11 hits**
...... Clostridium scatologenes	2	1	**Clostridium scatologenes hits**
.... Bacilli	11	5	
..... Bacillus	5	4	
...... Bacillus massiliogorillae	1	1	**Bacillus massiliogorillae hits**
...... Bacillus drentensis	1	1	**Bacillus drentensis hits**
...... Bacillus sp. OV166	2	1	**Bacillus sp. OV166 hits**
...... Bacillus massilioanorexius	1	1	**Bacillus massilioanorexius hits**
..... Lactobacillus reuteri	6	1	**Lactobacillus reuteri hits**
. Myoviridae	11	6	
.. Clostridium phage phiCDHM11	2	1	**Clostridium phage phiCDHM11 hits**
.. unclassified Myoviridae	9	5	
... Clostridium phage phiCDHM14	1	1	**Clostridium phage phiCDHM14 hits**
... Clostridium phage phiCDHM13	2	1	**Clostridium phage phiCDHM13 hits**
... Clostridium phage phiCD506	2	1	**Clostridium phage phiCD506 hits**
... Clostridium phage phiCDHM19	2	1	**Clostridium phage phiCDHM19 hits**

... Clostridium phage phiCD481-1	2	1	Clostridium phage phiCD481-1 hits

BLAST is a registered trademark of the National Library of Medicine

Support center Mailing list YouTube

- NATIONAL LIBRARY OF MEDICINE National Library Of Medicine
- NIH National Institutes Of Health
- U.S. Department of Health & Human Services
- USA.gov Government Made Easy USA.gov

NCBI

National Center for Biotechnology Information, U.S. National Library of Medicine 8600 Rockville Pike, Bethesda MD, 20894 USA

Policies and Guidelines | Contact
