# Supplementary material for: The Protozoan Trichomonas vaginalis Targets Bacteria with Laterally Acquired NlpC/P60 Peptidoglycan Hydrolases
Source: mBio. 2018 Dec 11;9(6):e01784-18. doi: 10.1128/mBio.01784-18 (PMC6299479; doi:10.1128/mBio.01784-18)
Supplement: TABLE S4 [file mbo006184213st4.pdf]

Tax BLAST report

DELTA-BLAST (Domain Enhanced Lookup Time Accelerated BLAST)

Tax BLAST report

**RID**	P7WNCUUV015 (Expires on 07-12 05:33 am)		
**Query ID**	XP_001276902.1	**Database Name**	nr
**Description**	Clan CA, family C40, NlpC/P60 superfamily cysteine peptidase [Trichomonas vaginalis G3]	**Description**	All non-redundant GenBank CDS translations+PDB+SwissProt+ excluding environmental samples from WGS projects
**Molecule type**	amino acid		
**Query Length**	275	**Program**	BLASTP 2.6.1+

Lineage Report

Organism Report Taxonomy Report

Organism	Blast Name	Score	Number of Hits	Description
Eukaryota	eukaryotes		28	
.Trichomonas vaginalis G3	trichomonads	562	16	**Trichomonas vaginalis G3 hits**
.Ascochyta rabiei	ascomycetes	76.6	1	**Ascochyta rabiei hits**
.Alternaria alternata	ascomycetes	72.0	2	**Alternaria alternata hits**
.Anaeromyces robustus	fungi	66.6	2	**Anaeromyces robustus hits**
.Dorcoceras hygrometricum	eudicots	58.2	1	**Dorcoceras hygrometricum hits**
.Monomorium pharaonis	ants	56.6	1	**Monomorium pharaonis hits**
.Penicillium coprophilum	ascomycetes	58.5	1	**Penicillium coprophilum hits**
.Oidiodendron maius Zn	ascomycetes	58.2	1	**Oidiodendron maius Zn hits**
.Oryza sativa Indica Group	monocots	56.6	1	**Oryza sativa Indica Group hits**
.Hyalella azteca	amphipods	56.6	1	**Hyalella azteca hits**
.Penicillium griseofulvum	ascomycetes	56.2	1	**Penicillium griseofulvum hits**

Organism Report

Lineage Report Taxonomy Report

Description	Score	E value	Accession
Trichomonas vaginalis G3 [trichomonads] Next Previous			

First

Clan CA, family C40, NlpC/P60 superfamily cysteine peptidase [Trichomonas vaginalis G3]	562	0.0	XP_001276902
Clan CA, family C40, NlpC/P60 superfamily cysteine peptidase [Trichomonas vaginalis G3]	562	0.0	EAY23654
Clan CA, family C40, NlpC/P60 superfamily cysteine peptidase [Trichomonas vaginalis G3]	518	0.0	XP_001583075
Clan CA, family C40, NlpC/P60 superfamily cysteine peptidase [Trichomonas vaginalis G3]	518	0.0	EAY22089
Clan CA, family C40, NlpC/P60 superfamily cysteine peptidase [Trichomonas vaginalis G3]	265	1e-85	XP_001330233
Clan CA, family C40, NlpC/P60 superfamily cysteine peptidase [Trichomonas vaginalis G3]	265	1e-85	EAY01385
Clan CA, family C40, NlpC/P60 superfamily cysteine peptidase [Trichomonas vaginalis G3]	150	1e-42	XP_001314869
Clan CA, family C40, NlpC/P60 superfamily cysteine peptidase [Trichomonas vaginalis G3]	150	1e-42	EAY02646
Clan CA, family C40, NlpC/P60 superfamily cysteine peptidase [Trichomonas vaginalis G3]	76.6	3e-14	XP_001326856
Clan CA, family C40, NlpC/P60 superfamily cysteine peptidase [Trichomonas vaginalis G3]	76.6	3e-14	EAY14633
Clan CA, family C40, NlpC/P60 superfamily cysteine peptidase [Trichomonas vaginalis G3]	67.0	7e-11	XP_001321089
Clan CA, family C40, NlpC/P60 superfamily cysteine peptidase [Trichomonas vaginalis G3]	67.0	7e-11	EAY08866
Clan CA, family C40, NlpC/P60 superfamily cysteine peptidase [Trichomonas vaginalis G3]	65.9	2e-10	XP_001310764
Clan CA, family C40, NlpC/P60 superfamily cysteine peptidase [Trichomonas vaginalis G3]	65.9	2e-10	EAX97834
Clan CA, family C40, NlpC/P60 superfamily cysteine peptidase [Trichomonas vaginalis G3]	57.0	3e-07	XP_001327845
Clan CA, family C40, NlpC/P60 superfamily cysteine peptidase [Trichomonas vaginalis G3]	57.0	3e-07	EAY15622

Didymella rabiei
[
ascomycetes
]
Next
Previous
First

hypothetical protein ST47_g1245 [Ascochyta rabiei]	76.6	5e-14	KZM27653

Alternaria alternata
[
ascomycetes
]
Next
Previous
First

hypothetical protein CC77DRAFT_1015013, partial [Alternaria alternata]	72.0	6e-11	XP_018378630
hypothetical protein CC77DRAFT_1015013, partial [Alternaria alternata]	72.0	6e-11	OAG13209

Anaeromyces sp. KS-2015

[
fungi
]
Next
Previous
First

hypothetical protein BCR32DRAFT_330850 [Anaeromyces robustus]	66.6	1e-09	ORX63502
hypothetical protein BCR32DRAFT_273178 [Anaeromyces robustus]	65.5	3e-09	ORX64239

Boea hygrometrica
[
eudicots
]
Next
Previous
First

hypothetical protein F511_29954 [Dorcoceras hygrometricum]	58.2	2e-07	KZV15205

Monomorium pharaonis (pharaoh ant)
[
ants
]
Next
Previous
First

PREDICTED: uncharacterized protein LOC105829055 [Monomorium pharaonis]	56.6	8e-07	XP_012523160

Penicillium coprophilum
[
ascomycetes
]
Next
Previous
First

hypothetical protein PENCOP_c009G04721 [Penicillium coprophilum]	58.5	9e-07	OQE38039

Oidiodendron maius Zn
[
ascomycetes
]
Next
Previous
First

hypothetical protein OIDMADRAFT_142915 [Oidiodendron maius Zn]	58.2	1e-06	KIN05178

Oryza sativa Indica Group (long-grained rice)
[
monocots
]
Next
Previous
First

hypothetical protein OsI_36595 [Oryza sativa Indica Group]	56.6	1e-06	EEC68419

Hyalella azteca
[
amphipods
]
Next
Previous
First

PREDICTED: murein DD-endopeptidase MepS/Murein LD-carboxypeptidase-like [Hyalella azteca]	56.6	2e-06	XP_018006135
Penicillium griseofulvum<br>[<br>ascomycetes<br>]<br>Next<br>Previous<br>First			
hypothetical protein PGRI_034460 [Penicillium griseofulvum]	56.2	5e-06	KXG45679

Taxonomy Report

Organism Report Lineage Report

Taxonomy	Number of hits	Number of Organisms	Description
Eukaryota	28	11	
. Trichomonas vaginalis G3	16	1	Trichomonas vaginalis G3 hits
. Opisthokonta	10	8	
.. Fungi	8	6	
... leotiomyceta	6	5	
.... Pleosporineae	3	2	
..... Ascochyta rabiei	1	1	Ascochyta rabiei hits
..... Alternaria alternata	2	1	Alternaria alternata hits
.... Penicillium	2	2	
..... Penicillium coprophilum	1	1	Penicillium coprophilum hits
..... Penicillium griseofulvum	1	1	Penicillium griseofulvum hits
.... Oidiodendron maius Zn	1	1	Oidiodendron maius Zn hits
... Anaeromyces robustus	2	1	Anaeromyces robustus hits
.. Pancrustacea	2	2	
... Monomorium pharaonis	1	1	Monomorium pharaonis hits
... Hyalella azteca	1	1	Hyalella azteca hits
. Mesangiospermae	2	2	
.. Dorcoceras hygrometricum	1	1	Dorcoceras hygrometricum hits
.. Oryza sativa Indica Group	1	1	Oryza sativa Indica Group hits

BLAST is a registered trademark of the National Library of Medicine

Support center Mailing list YouTube

- National Library Of Medicine

- National Institutes Of Health
- U.S. Department of Health & Human Services
- USA.gov

NCBI

National Center for Biotechnology Information, U.S. National Library of Medicine 8600 Rockville Pike, Bethesda MD, 20894 USA

Policies and Guidelines | Contact
