# Supplementary material for: The Protozoan Trichomonas vaginalis Targets Bacteria with Laterally Acquired NlpC/P60 Peptidoglycan Hydrolases
Source: mBio. 2018 Dec 11;9(6):e01784-18. doi: 10.1128/mBio.01784-18 (PMC6299479; doi:10.1128/mBio.01784-18)
Supplement: TABLE S5 [file mbo006184213st5.pdf]

Homogenous model: LG based model

Tree #	logL	deltaL	bp-RELL	p-AU	Tree description	
1	-7665.573	0.000	0.428	0.657	ML tree PhyML (LG+G4+I) illustrated in Figure 1	Unconstrained analyses
2	-7667.111	1.538	0.288	0.661	ML tree PhyML (LG+G4+I+F)	
3	-7674.212	8.639	0.101	0.300	ML tree IQ-TREE (LG4X+R+F)	
4	-7683.355	17.782	0.069	0.168	ML tree IQ-TREE (C20+R+F)	
5	-7675.471	9.899	0.112	0.284	All 9 TvNlpC_P60 monophyletic IQ-TREE ML LG+G4+I	Constrained analyses
6	-7720.044	54.472	0.000	0.003	All 9 TvNlpC_P60 & Acantamoeba monophyletic IQ-TREE ML LG+G4+I	
7	-7693.098	27.526	0.003	0.023	All 9 TvNlpC_P60 & 2 Fungi monophyletic IQ-TREE ML LG+G4+I	
8	-7715.600	50.028	0.000	0.000	All 9 TvNlpC_P60 & Trichuris monophyletic IQ-TREE ML LG+G4+I	

Command line: iqtree -s 50T_104S_ALN.phy -st AA -m LG+I+G4 -n 0 -z 50T_104S_8_trees.treefile -zb 10000 -zw -au

Protein mixture model: LG4X based model

Tree #	logL	deltaL	bp-RELL	p-AU	Tree description	
1	-7742.676	11.015	0.101	0.259	ML tree PhyML (LG+G4+I) illustrated in Figure 1	Unconstrained analyses
2	-7734.958	3.297	0.242	0.582	ML tree PhyML (LG+G4+I+F)	
3	-7731.661	0.000	0.502	0.770	ML tree IQ-TREE (LG4X+R+F)	
4	-7753.263	21.603	0.079	0.143	ML tree IQ-TREE (C20+R+F)	
5	-7743.809	12.148	0.074	0.219	All 9 TvNlpC_P60 monophyletic IQ-TREE ML LG+G4+I	Constrained analyses
6	-7784.656	52.995	0.000	0.002	All 9 TvNlpC_P60 & Acantamoeba monophyletic IQ-TREE ML LG+G4+I	
7	-7759.530	27.870	0.002	0.007	All 9 TvNlpC_P60 & 2 Fungi monophyletic IQ-TREE ML LG+G4+I	
8	-7773.514	41.853	0.001	0.006	All 9 TvNlpC_P60 & Trichuris monophyletic IQ-TREE ML LG+G4+I	

Command line: iqtree -s 50T_104S_ALN.phy -st AA -m LG4X+R4+F -n 0 -z 50T_104S_8_trees.treefile -zb 10000 -au

Protein mixture mode: C20 based model

Tree #	logL	deltaL	bp-RELL	p-AU	Tree description	
1	-7587.219	12.083	0.080	0.197	ML tree PhyML (LG+G4+I) illustrated in Figure 1	Unconstrained analyses
2	-7588.024	12.888	0.082	0.209	ML tree PhyML (LG+G4+I+F)	
3	-7594.232	19.096	0.020	0.062	ML tree IQ-TREE (LG4X+R+F)	
4	-7575.136	0.000	0.807	0.919	ML tree IQ-TREE (C20+R+F)	
5	-7600.048	24.912	0.008	0.045	All 9 TvNlpC_P60 monophyletic IQ-TREE ML LG+G4+I	Constrained analyses
6	-7635.147	60.011	0.000	0.000	All 9 TvNlpC_P60 & Acantamoeba monophyletic IQ-TREE ML LG+G4+I	
7	-7612.661	37.524	0.003	0.011	All 9 TvNlpC_P60 & 2 Fungi monophyletic IQ-TREE ML LG+G4+I	
8	-7626.159	51.022	0.000	0.001	All 9 TvNlpC_P60 & Trichuris monophyletic IQ-TREE ML LG+G4+I	

Command line: iqtree -s 50T_104S_ALN.phy -st AA -m C20+R4 -n 0 -z 50T_104S_8_trees.treefile -zb 10000 -au

Maximum likelihood (ML) tree for the given model (green)
deltaL : logL difference from the ML tree
bp-RELL: Bootstrap Resampling estimated log-likelihoods (RELL) posterior weights for the compared trees (sum = 1 for all trees)
p-AU: p-value for the Approximately Unbiased (AU) test among compared trees
p-value < 0.05 (orange)

A combination of (i) more draditional empirical models (LG based) and (ii) more recent mixture models (LG4X and C20 based) were used to explore the phylogenetic relationship of NlpC/P60 proteins.

The empirical models are related to models used in past publications to infer NlpC/P60 phylogenies (using distance or maximum likelihood methods) and provide a reference point (see references #18, 28, 32, 33 from the main text). Mixture models do explicitly encode the fact that biochemical constraints are site-specific features and were shown to provide a better fit than empirical matrices in several case,s including saturation (i.e. multiple substitutions), making these less susceptible to phylogenetic artifacts. The improved maximul likelihood values for the mixture models is consistent with this. The four unconstrained analyses infered slighlty different phylogenetic relationships. These were however all consistent in supporting two distinct LGT events for the TvNlpC/P60 genes.
Four constrained analyses performed with the empirical model LG+G4+I were used to further investigate the strength of the phylogenetic signal supporting the LGT events for the TvNlpC/P60 genes. Notably, the more complex mixture model (C20 based) rejected all four constained phylogenetic relationships, consistent with multiple independent LGT events among the samples eukaryotes, including two distinct LGT events for the two TvNLpC/P60 families A and B.
