# Supplementary material for: The Protozoan Trichomonas vaginalis Targets Bacteria with Laterally Acquired NlpC/P60 Peptidoglycan Hydrolases
Source: mBio. 2018 Dec 11;9(6):e01784-18. doi: 10.1128/mBio.01784-18 (PMC6299479; doi:10.1128/mBio.01784-18)
Supplement: TABLE S6 [file mbo006184213st6.pdf]

**Crystallisation and data collection**				
Name/PDB id	NlpC1		NlpC1MX	NlpC2
Space group	$P2_122_1$		$P2_122_1$	P1
a, b, c (Å)	53.1 70.0 79.9		53.1 70.1 80.0	36.8 99.0 102.8
α, β, □ (◦)	90.0 90.0 90.0		90.0 90.0 90.0	115.0 96.2 94.2
Beamline	Rotating anode		MX1	Rotating anode
Data	Native	SeMet		
Wavelength (Å)	1.5418	1.5418	0.9537	1.5418
Resolution range (Å)	44.33-1.86 (1.9-1.86)	29.24-1.54 (1.57-1.54)	21.4-1.2 (1.22-1.2)	50.9-2.3 (2.37-2.3)
No. of observations	183271(7185)	252839(8758)	663880(31317)	154350(10464)
No. of unique reflections	25504(1152)	43999(1744)	94028(4588)	53546(4164)
Completeness (%)	98.2(73.3)	98.8(81)	100(100)	93(89.3)
Multiplicity	7.2(6.2)	5.7(5.0)	7.1(6.8)	2.9(2.5)
Mean I/σ(I)	14.7(3.7)	14.1(4.1)	9.7(1.7)	6.9(2)
Rpim (%)	4.0(18.9)	3.0(11.2)	5.6(53.8)	7.3(32.7)
**Model and refinement statistics**				
PDB id		6BIM	6BIO	6BIQ
Resolution range (Å)		29.0-1.54	21.3-1.2	35.6-2.3
No. of reflections (total)		43961	93974	53533
No. of reflections (test)		4124	8843	4852
Rcryst (%)		15.39	16.3	19.8
Rfree (%)		17.53	19.1	24.1
**Stereochemical parameters**				
Rmsd bond lenghts (Å)		0.003	0.012	0.006
Rmsd bond angles (°)		0.676	1.205	0.803
MolProbity score				
All atom clash score		2.02	0.67	1.84
Ramachandran plot (%)		97.16	97.17	95.9
Rotamer outliners (%)		0.81	0	2.1
No. of non-H atoms				
Protein		2267 (275)	2267 (275)	8438 (1056)
Waters		516	422	571
Total		2783	2689	9009
Average B-value (Å^2)				
Protein		16.28	13.27	33.7
Water		28.94	25.6	25.8
No. of residues/chains in ASU		275/1	275/1	1056/4

NlpC1 is NlpC_A1 (TVAG 119910)
NlpC2 is NlpC_A2 (TVAG 457240)
