# Supplementary figures and images for: The Protozoan Trichomonas vaginalis Targets Bacteria with Laterally Acquired NlpC/P60 Peptidoglycan Hydrolases
Source: mBio. 2018 Dec 11;9(6):e01784-18. doi: 10.1128/mBio.01784-18 (PMC6299479; doi:10.1128/mBio.01784-18)

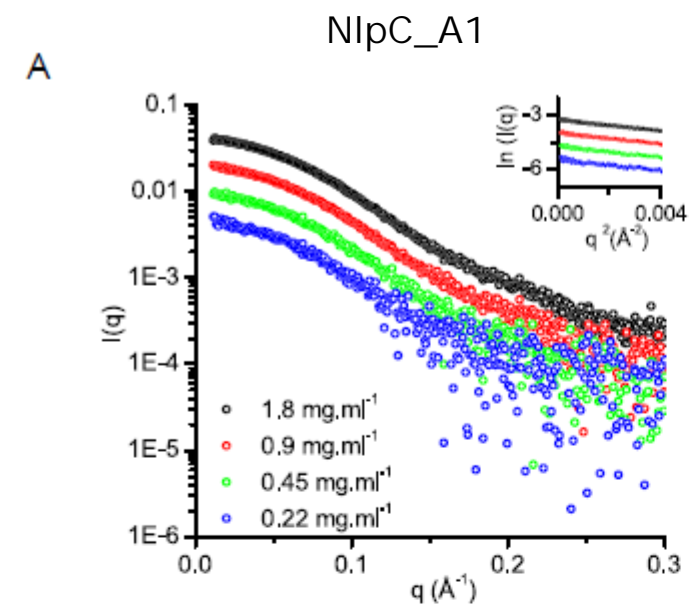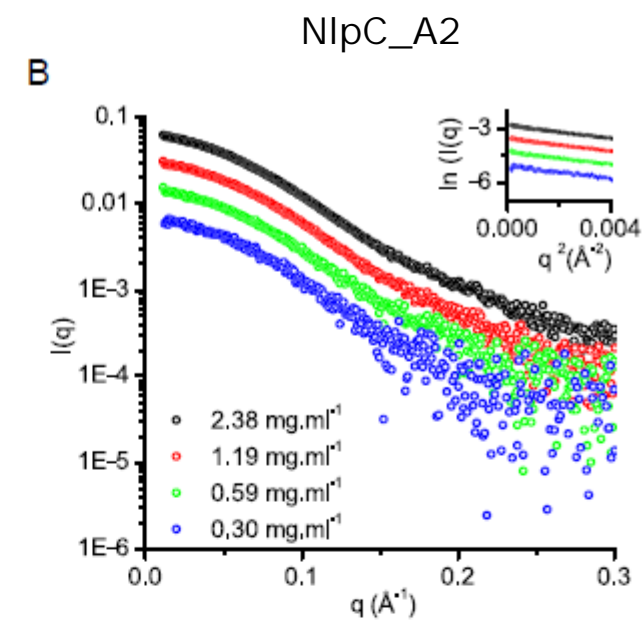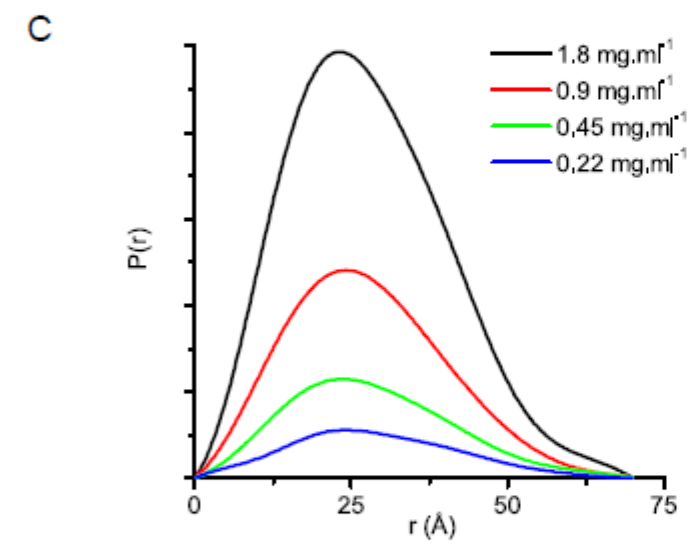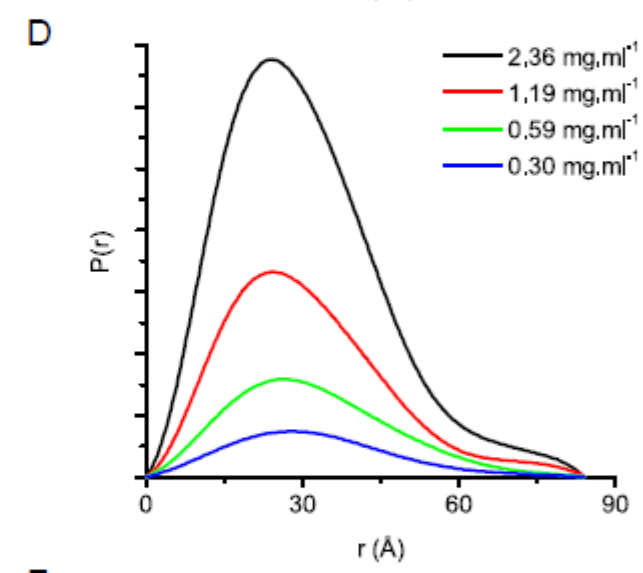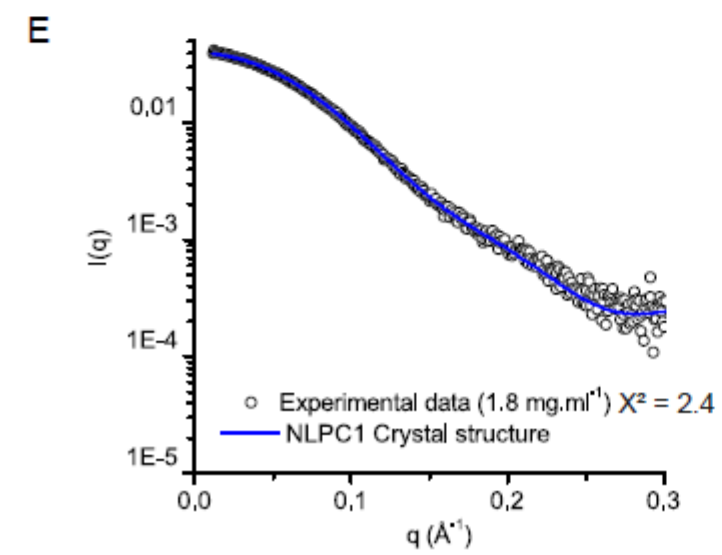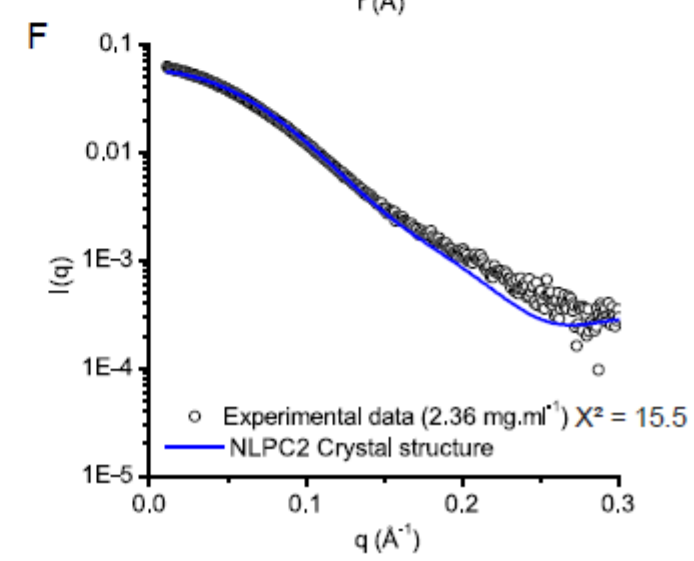

Supplement: FIG S2 [file mbo006184213sf2.pdf]

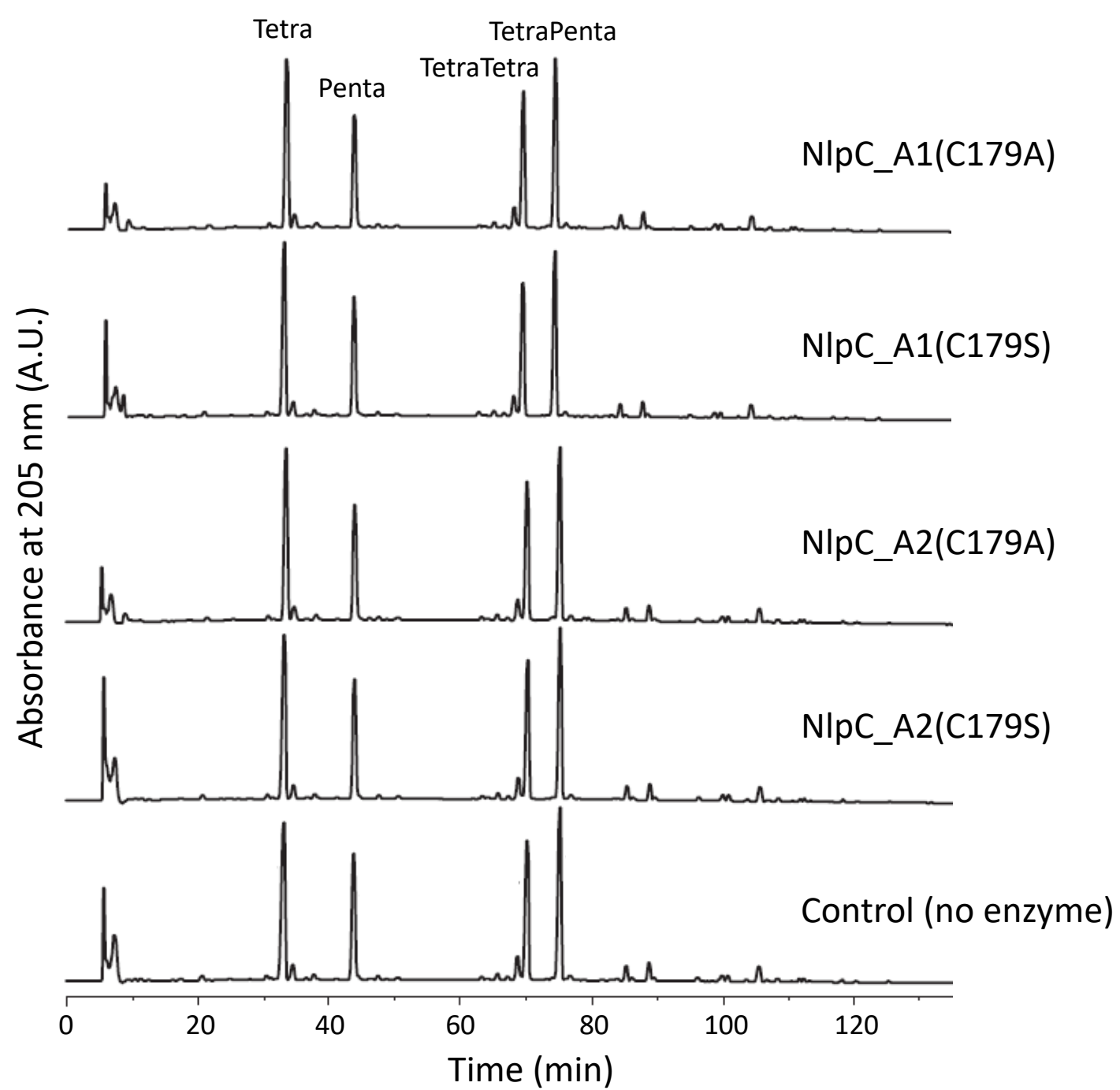

Supplement: FIG S3 [file mbo006184213sf3.pdf]
