# Supplementary material for: The Protozoan Trichomonas vaginalis Targets Bacteria with Laterally Acquired NlpC/P60 Peptidoglycan Hydrolases
Source: mBio. 2018 Dec 11;9(6):e01784-18. doi: 10.1128/mBio.01784-18 (PMC6299479; doi:10.1128/mBio.01784-18)
Supplement: TABLE S7 [file mbo006184213st7.pdf]

NlpC_A1 SAXS data

Conc. , mg/ml	R g , A (autoRg, guinier)	I(0)	D max, A	MM , kDa
1.8	21.46 (0.12)	0.04	70	29
0.9	21.27 (0.69	0.019	70	27
0.45	21.72 (0.93)	0.0094	70	27
0.22	21.47(1.66)	0.0045	68	26
0.11	21.81 (1.25)	0.0021	73	25

NlpC_A2 SAXS data

Conc. , mg/ml	R g , A (autoRg, guinier)	I(0)	D max, A	MM , kDa
2.36	23.69 (0.47)	0.061	84	33
1.18	23.68 (0.51)	0.03	83	33
0.59	23.13 (0.17)	0.014	78	30
0.3	23.12 (0.32)	0.0065	78	28
0.15	21.8 (0.65)	0.003	78	26
